# Three undescribed dihydrostilbene glycosides from leaves of *Camellia oleifera* Abel. And their anti-inflammatory activity

**DOI:** 10.1016/j.heliyon.2024.e30507

**Published:** 2024-04-30

**Authors:** Yi Xu, Si-Qi Tang, Zong-Wu Suo, Kai-Xin Wei, Walter Luyten, Hao Huang, Xiao-Jun Li

**Affiliations:** a National Engineering Research Center for Modernization of Traditional Chinese Medicine - Hakka Medical Resources Branch, School of Pharmacy, Gannan Medical University, Ganzhou, 341000, China; b First Affiliated Hospital of Gannan Medical University, Ganzhou, 341000, China; c Department of Biology, KU Leuven, Leuven, Belgium

**Keywords:** *Camellia oleifera* Abel., Dihydrostilbene glycosides, Anti-inflammatory, Oleiferaside A, Oleiferaside B, Oleiferaside C

## Abstract

Three previously unidentified dihydrostilbene glycosides, named oleiferaside A (**1**), oleiferaside B (**2**), and oleiferaside C (**3**), were discovered through a phytochemical exploration on *Camellia oleifera* Abel. leaves. Additionally, nine known secondary metabolites (**4–12**) were also identified. The undescribed secondary metabolites **1**–**3** were elucidated as 3,5-dimethoxydihydrostilbene 4′-*O*-*α*-l-arabinofuranosyl-(1 → 6)-*β*-d- glucopyranoside, 3,5-dimethoxydihydrostilbene 4′-*O*-*α*-l-arabinopyranosyl-(1 → 6)-*β*-d- glucopyranoside and 3,5-dimethoxydihydrostilbene 4′-*O*-*β*-d-apiofuranosyl-(1 → 6)-*β*-d- glucopyranoside, respectively. HR-MS and NMR spectroscopy were utilized for determining the structures of the isolates. The natural products were assessed for their anti-inflammatory effect using RAW264.7 macrophage stimulated by LPS. The findings demonstrated that compounds **1–4** exhibited inhibitory activities on NO and PGE_2_ production without causing cytotoxicity. These observations suggest that these compounds may have potential anti-inflammatory properties.

## Highlights

• The phytochemical investigation on the leaves of *C. oleifera* resulted in the discovery of twelve compounds.
• Three previously undescribed compounds, oleiferaside A-C (**1-3**), were isolated from *C. oleifera* leaves.
• The tested dihydrostilbene glycosides (**1-4**) showed potential anti-inflammatory activities with no cytotoxicity.

## 1 Introduction

**Fig. 1:**
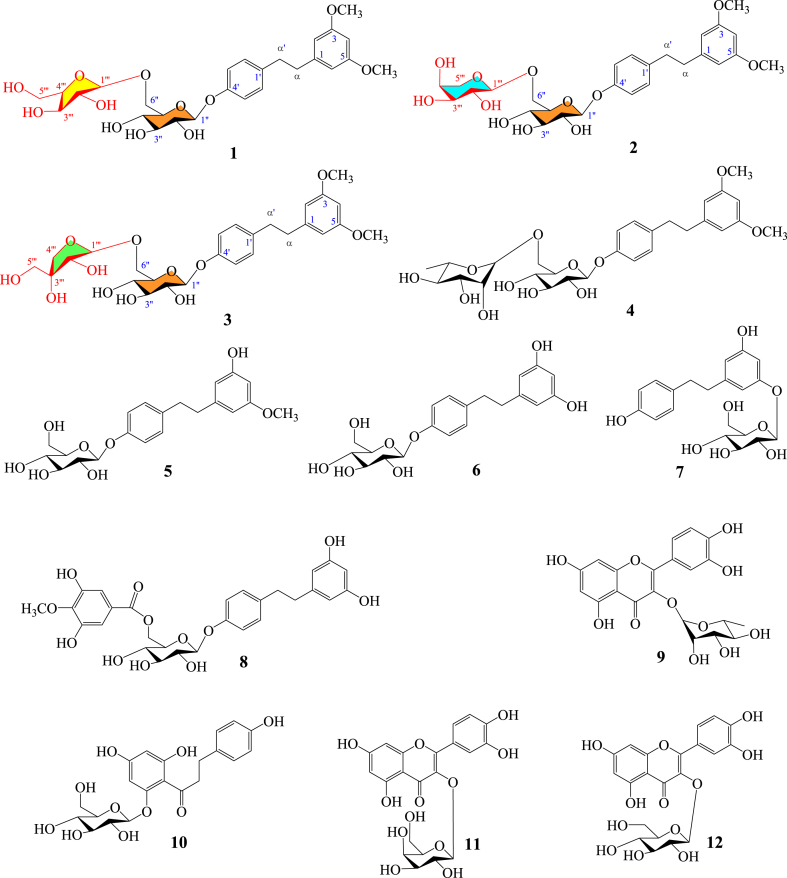
Chemical structures of compounds **1–12.**

*Camellia oleifera* Abel., a member of the *Camellia* genus (Theaceae family), has a wide distribution across several provinces in China, including Jiangxi, Hunan, Fujian, Guangdong, and Guangxi [1]. Recognized as a traditional Chinese herb-medicine, the dried leaves of *C. oleifera* are officially listed in the National Compilation of Chinese Herbal Medicine and Chinese Materia Medica. The plant leaves are known for their various therapeutic properties, including clearing heat and detoxifying, astringent hemostasis, refreshing the brain, promoting blood circulation and dispersing stasis, relieving pain. They have been traditionally employed as a remedy for conditions such as epistaxis, skin ulceration and itching, ulcerative gangrene, acute pharyngitis, stomachache, as well as sprain and contusion [2,3]. The identification of triterpenoids and their saponins, flavonoids and their glycosides, dihydrostilbenes and their glycosides, anthraquinones, lignans, phenols, organic acids, steroids, and fatty acids have been significant outcomes of previous research conducted on this plant [[4], [5], [6], [7], [8], [9], [10]]. These compounds have exhibited diverse pharmacological actions, such as anti-tumor effects [4], anti-inflammatory properties [5], anti-neuroinflammatory and neuroprotective effects [11,12], antioxidant activity [8,13,14], antibacterial effects [5], hypoglycemic effects [15,16], and anti-thrombotic effects [17]. In our current study, we focused on the methanol extracts of *C. oleifera* leaves and identified three new bioactive dihydrostilbene glycosides (**1–3**) and nine known secondary metabolites (**4–12**) (Fig. 1). This article presents the isolation, structure determination, and assessment of the cytotoxicity and anti-inflammatory behaviour of these natural products.

## 2 Materials and methods

### 2.1 Instrument and reagent

An AUTOPOL I automatic polarimeter from Rudolph Research Analytical (U.S.A) was used for optical rotation test. A SPECORD 50 PLUS UV spectrophotometer purchased from Analytik Jena AG (Germany) was employed for UV spectra. A NICOLETIS50 FT-IR spectrometer from Thermo Fisher (Germany) was applied for IR spectra. An AVANCE III Bruker-400 spectrometer (Switzerland) was utilized for NMR spectra. Residual solvent peaks were used as references for the chemical shifts of NMR. A Xevo G2-XS TOF instrument (Waters) was operated for obtaining MS data. The instruments and reagents used below, including silica gel, TLC, C_18_ column, semi-preparative HPLC system, LPS, MTT, FBS, DMEM, and ELISA kits for NO and PGE_2_, are consistent with our previous literature reports [18].

### 2.2 Plant materials

The leaves of *Camellia oleifera* Abel. were gathered from their natural habitat in Xingguo, Jiangxi Province, China, in May 2020. Then, the medicinal materials were verified by Xiao-Jun Li from the Department of Pharmacy at Gannan Medical University. The voucher specimens have been stored at the Key Laboratory of Natural Product Research and Development in Jiangxi Province Universities, Gannan Medical University, under the reference number CO202005.

### 2.3 Extraction and purification

2.8 kg of dried leaves of *C. oleifera* were pulverized into powder and subjected to three extractions using 70 % methanol (3 × 10 L) at 60 °C with reflux for 2 h on each occasion. Afterwards, the solvent was evaporated, resulting in a MeOH extract that was subsequently dispersed in distilled water. Sequential partitioning was carried out using organic solvents with different polarities for giving petroleum ether part (36.7 g), EtOAc part (201 g), and n-BuOH part (357.4 g), respectively.

The 201 g of EtOAc part was first isolated by silica gel column chromatography (CC). The elution process involved a gradient of CH_2_Cl_2_–CH_3_OH with varying ratios of 100:1, 50:1, 30:1, 20:1, 10:1, 5:1, 3:1, 1:1, and 1:3 (v/v). Each fraction was collected, yielding seven major fractions (E1-E7) by TLC analysis. 84.55 g of E5 was performed on a silica gel CC applying a mobile phase of dichloromethane/methanol/water (10:2:0.1–1:3:0.2, v/v/v). This process yielded six sub-fractions (E5.1-E5.6). A C_18_ semi-preparative HPLC was operated for the further purification of E5.4 (350 mg), using a gradient elution of methanol/water (flow rate: 3 mL/min, 30:70–70:30, v/v). As a result, compounds **4** (132.2 mg), **9** (18.4 mg), **5** (7.1 mg), **1** (12.9 mg), **2** (8.3 mg), **3** (7.3 mg), and **10** (12.6 mg) were obtained consecutively. E5.6 (126 mg) was chromatographed by C_18_ semi-preparative HPLC using a mobile phase of methanol/water (flow rate: 3 mL/min, 30:70–70:30, v/v). This process resulted in the isolation of compounds **6** (7.1 mg) and **7** (8.6 mg), along with sub-fractions E5.6.1-E5.6.3. Subsequently, in order to obtain compounds **11** (5.9 mg) and **12** (7.6 mg), a C_18_ semi-preparative HPLC method was established for the further purification of E5.6.1 (30.2 mg), with the gradient elution of methanol/water (flow rate: 3 mL/min, 20:70–50:50, v/v). Simultaneously, compounds **8** (6.8 mg) and **10** (8.7 mg) were yield from E5.6.2 (33.7 mg) via purification of C_18_ semi-preparative HPLC using a elution of methanol/water (flow rate: 3 mL/min, 50:50, v/v).

### 2.4 Spectroscopic data

Oleiferaside A (**1**): white amorphous powder; HR-ESI-MS *m/z:* 575.2092 [M + Na]^+^ (calcd. for C_27_H_36_O_12_Na, 575.2099); molecular formula: C_27_H_36_O_12_; UV (MeOH) *λ*_max_ (log *ε*) 203 (0.94), 218 (0.41); [α]_20_^D^ −69.97 (*c* 0.23, MeOH); IR (microscope) *ν*_max_ 3650, 1635, 1540, 1507, 1150, 1074, 831, 685 cm^−1^; NMR data, refer to Table 1.

**Table 1:** NMR data of undescribed compounds **1–3**. ^1^H/400 MHz,^13^C/100 MHz, *δ* in ppm, *J* in Hz.

Position	1^a,b^		2^a,b^		3^a,b^	
	*δ*_H_	*δ*_C_	*δ*_H_	*δ*_C_	*δ*_H_	*δ*_C_
1	–	145.3	–	145.3	–	145.3
2	6.30 (d, *J* = 2.2)	107.6	6.30 (d, *J* = 2.2)	107.6	6.30 (d, *J* = 2.2)	107.6
3	–	162.2	–	162.2	–	162.2
4	6.27 (d, *J* = 2.2)	98.9	6.27 (d, *J* = 2.2)	98.9	6.27 (d, *J* = 2.2)	98.9
5	–	162.2	–	162.2	–	162.2
6	6.30 (d, *J* = 2.2)	107.6	6.30 (d, *J* = 2.2)	107.6	6.30 (d, *J* = 2.2)	107.6
α	2.80 (m)	39.5	2.80 (m)	39.5	2.80 (m)	39.5
α′	2.81 (m)	38.1	2.81 (m)	38.1	2.81 (m)	38.1
1′	–	137.1	–	137.1	–	137.1
2′	7.10 (d, *J* = 8.6)	130.5	7.10 (d, *J* = 8.6)	130.5	7.10 (d, *J* = 8.6)	130.5
3′	7.00 (d, *J* = 8.6)	117.6	7.00 (d, *J* = 8.6)	117.6	7.00 (d, *J* = 8.6)	117.6
4′	–	157.4	–	157.4	–	157.4
5′	7.00 (d, *J* = 8.6)	117.6	7.00 (d, *J* = 8.6)	117.6	7.00 (d, *J* = 8.6)	117.6
6′	7.10 (d, *J* = 8.6)	130.5	7.10 (d, *J* = 8.6)	130.5	7.10 (d, *J* = 8.6)	130.5
3,5-OCH_3_	3.71 (s)	55.6	3.71 (s)	55.6	3.71 (s)	55.6
Sugar moiety	Glc		Glc		Glc	
1″	4.83 (d, *J* = 7.3)	102.5	4.85 (d, *J* = 7.2)	102.3	4.81 (d, *J* = 7.5)	102.6
2″	3.43 (m)	74.9	3.43 (m)	74.9	3.43 (m)	74.9
3″	3.43 (overlapped)	77.9	3.43 (overlapped)	77.8	3.43 (overlapped)	77.9
4″	3.36 (m)	71.8	3.37 (m)	71.5	3.35 (m)	71.6
5″	3.59 (m)	76.8	3.62 (m)	77.3	3.59 (m)	77.0
6a′′	3.60 (m)	68.2	3.62 (overlapped)	69.3	3.61 (m)	68.8
6b′′	4.05 (dd, *J* = 5.4, 14.3)		4.10 (dd, *J* = 2.1, 11.5)		4.01 (dd, *J* = 1.5, 10.6)	
Sugar moiety	Araf		Arap		Apif	
1‴	4.93 (d, *J* = 1.5)	110.0	4.30 (d, *J* = 6.8)	104.9	4.98 (d, *J* = 2.4)	111.1
2‴	4.00 (dd, *J* = 3.4, 1.5)	83.3	3.57 (m)	72.5	3.91 (d, *J* = 2.4)	78.0
3‴	3.82 (dd, *J* = 6.0, 3.4)	78.9	3.49 (dd, *J* = 3.3, 8.9)	74.1	–	80.5
4‴	3.96 (m)	85.8	3.75 (m)	69.5	3.76 (d, *J* = 9.6)	75.0
					3.98 (d, *J* = 9.6)	
5‴	3.62 (m)	63.0	3.42 (m)	66.7	3.58 (s)	65.5
	3.70 (m, overlapped)		3.82 (dd, *J* = 3.1, 12.4)			

a) Recorded in CD_3_OD. b) Multiplicities inferred from DEPT and HSQC experiments. Glc: glucopyranose; Araf: arabinofuranose; Arap: arabinopyranose; Apif: apiofuranose.

Oleiferaside B (**2**): white amorphous powder; HR-ESI-MS *m/z* 575.2091 [M + Na]^+^ (calcd. for C_27_H_36_O_12_Na, 575.2099); molecular formula: C_27_H_36_O_12_; UV (MeOH) *λ*_max_ (log *ε*) 205 (2.25), 218 (1.04); [α]_20_^D^ −68.84 (*c* 0.05, MeOH); IR (microscope) *ν*_max_ 3628, 1593, 1540, 1508, 1455, 1204, 1066, 829, 683 cm^−1^; NMR data, refer to Table 1.

Oleiferaside C (**3**): white amorphous powder; HR-ESI-MS *m/z* 575.2091 [M + Na]^+^ (calcd. for C_27_H_36_O_12_Na, 575.2099); molecular formula: C_27_H_36_O_12_; UV (MeOH) *λ*_max_ (log *ε*) 205 (2.33), 218 (1.24); [α]_20_^D^ −25.31 (*c* 0.13, MeOH); IR (microscope) *ν*_max_ 3490, 1593, 1508, 1457, 1226, 1203, 1149, 1056, 823, 691 cm^−1^; NMR data, refer to Table 1.

### 2.5 Acidic hydrolysis of **1**–**3**

Acid hydrolysis and derivatization methods referred to previous studies [19,20]. In short, each of compounds **1–3** (1.5 mg each) was added to 2 M HCl (500 μL) and subjected to heating for 2 h at 90 °C. After the hydrolysis process, the reaction mixture was neutralized with 500 μL of 2 M NH_4_OH and subsequently dried using an evaporator. Hydrolyzed samples **1–3**, along with standard sugars (d-glucopyranose, l-arabinofuranose, l-arabinopyranose, and d-apiofuranose, 5 mg each), and 5 mg of l-cysteine methyl ester hydrochloride were dissolved in 1 mL of pyridine and reacted for 1 h at 60 °C. Subsequently, 5 μL of 2-methylphenyl isothiocyanate was introduced into the mixture, followed by an additional hour of heating. The resulting reaction mixture (20 μL) was then analyzed using RP-HPLC and detected at 250 nm to determine the types of sugars present. This was done by comparing with authentic samples, utilizing the elution system CH_3_CN–H_2_O in 0.1 % HCOOH (v/v, 15:85–35:65, flow rate: 0.8 mL/min). The *t*_R_ values obtained were 35.087 min for d-glucopyranose, 37.319 min for l-arabinofuranose, 37.011 min for l-arabinopyranose, and 44.219 min for d-apiofuranose (Table S1).

### 2.6 Cell culture

The mouse RAW264.7 macrophages (RRID: CVCL_0493) were purchased from the American Type Culture Collection (ATCC, Manassas, VA). The cell cultures employed in our investigation underwent examination and were validated to be free from mycoplasma contamination. Each RAW264.7 cell was cultured separately in DMEM culture medium with 100 U/mL of penicillin G, 100 mg/L of streptomycin, and 10 % heat-inactivated FBS. Following that, the RAW264.7 macrophage was placed in a controlled environment with 5 % CO_2_ at a temperature of 37 °C for incubation. Every 2 days, the culture medium was refreshed. When the cell bottle reached 80 % confluency, the previous medium was discarded, and the cells underwent 2–3 washes with PBS. Subsequently, the addition of 0.25 % trypsin facilitated cell digestion, and repeated pipetting with a pipette ensured even dispersion of the cells. The resulting single-cell suspension was seeded at a 1:5 ratio in the cell culture medium.

### 2.7 MTT assay for cell viability

The step-by-step protocols for MTT assay were documented in our prior studies [21,22]. The analysis was performed in triplicate, independently.

### 2.8 Nitrite assay

As stated in our previous research, we employed the ELISA kit to accurately quantify the levels of NO [21,22].

### 2.9 Prostaglandin E_2_ assay

The measurement of Prostaglandin E_2_ (PGE_2_) levels for selected compounds were conducted utilizing commercially obtainable kits. Three separate assays were carried out on the basis of operation instructions. Briefly, RAW264.7 macrophages were seeded in 24-well culture plates at a density of 5 × 10^4^ cells/well. The test compounds were exposed to varying concentrations of treatment, and subsequently stimulated with 1 μg/mL of LPS for 24 h. Following the incubation, the supernatant was collected and utilized for PGE_2_ concentration measurement via the application of a PGE_2_ ELISA kit.

### 2.10 Statistical analysis

One-way ANOVA was employed for statistical analysis of normally distributed data to assess variations among mean values. The findings are shown as the mean ± standard deviation (S.D.). The statistical significance was considered at p < 0.05.

## 3 Results and discussion

**Fig. 2:**
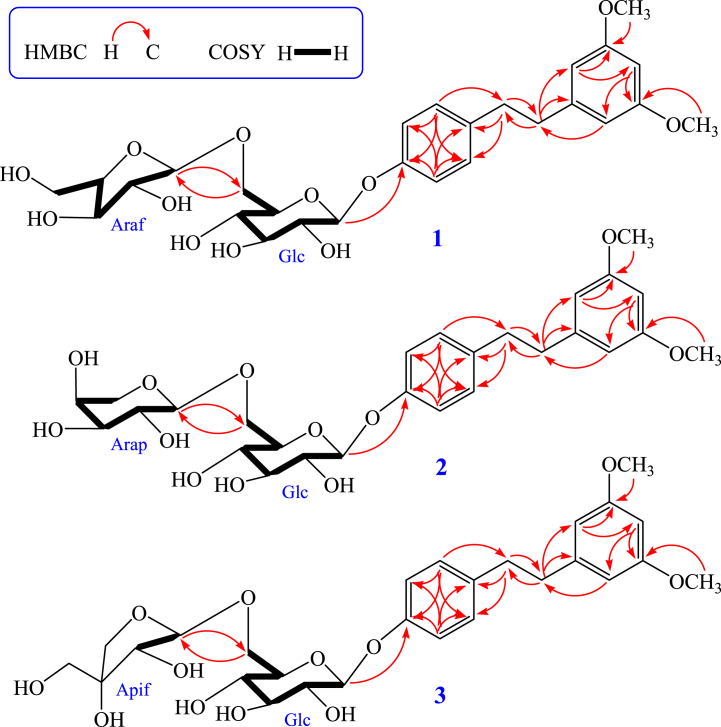
Important ^1^H–^1^H COSY (highlighted) and HMBC (indicated by arrows) correlations of undescribed compounds **1–3**.

Compound **1** was acquired in the form of a white amorphous powder. HR-ESI-MS data which revealed a *m/z* peak at 575.2092 [M+Na]^+^ (calculated for C_27_H_36_O_12_Na, 575.2099) demonstrated that the molecular formula was C_27_H_36_O_12_. The ^1^H NMR spectrum of compound **1** (recorded in CD_3_OD) exhibited the following signals: seven aromatic protons at *δ* 7.10 (2H, d, *J* = 8.6 Hz), 7.00 (2H, d, *J* = 8.6 Hz), 6.30 (2H, d, *J* = 2.2 Hz) and 6.27 (1H, d, *J* = 2.2 Hz); two methoxy groups at *δ* 3.71 (6H, s); two methylenes at *δ* 2.80 (2H, m) and 2.81 (2H, m). In addition, the analysis of the spectra indicated the presence of two sugars, as evidenced by two anomeric protons at *δ* 4.83 (1H, d, *J* = 7.3 Hz) and 4.93 (1H, d, *J* = 1.5 Hz). Confirmation of the presence of 27 carbon signals was achieved through additional analysis of the ^13^C NMR and DEPT spectra. Among these, 16 signals were attributed to a dihydrostilbene aglycone moiety, while the remaining 11 signals were assigned to a disaccharide residue. The dihydrostilbene skeleton of compound **1** was identified through the analysis of its ^1^H and ^13^C NMR spectra (refer to Table 1), with the presence of characteristic aromatic carbons at *δ* 145.3 (C-1), 107.6 (C-2, C-6), 162.2 (C-3, C-5), 98.9 (C-4) and 137.1 (C-1′), 130.5 (C-2′, C-6′), 117.6 (C-3′, C-5′), 157.4 (C-4′), two methylene carbons at *δ* 39.5 (C-α) and 38.1 (C-α′), respectively. The NMR spectral data of compound **1** closely resembled those of compound **4**, which had been previously documented from *C. oleifera* [7], except the presence of different sugar moiety (arabinofuranose) resonances [*δ*_H_ 4.93 (1H, d, *J* = 1.5 Hz, araf-H-1‴)/*δ*_C_ 110.0, 83.3, 78.9, 85.8, 63.0 (araf-C-1‴-5‴)] in **1**, which was located at glc-C-6′′ (*δ* 68.2), instead of rhamnopyranose in **4**. The coupling constants of anomeric protons glc-H-1′′ (*δ* 4.83, 1H, d, *J* = 7.3 Hz) and araf-H-1‴ (*δ* 4.93, 1H, d, *J* = 1.5 Hz) showed that the glucopyranose and arabinofuranose were *β*-positioned and *α*-positioned, respectively. Additionally, assignment of the sugar chain (6-*O*-*α*-l-arabinofuranosyl-*β*-d- glucopyranoside) was conducted through comparing its NMR spectral data with known compound 2-phenylethyl 6-*O*-*α*-l-arabinofuranosyl-*β*-d-glucopyranoside and literature values [23,24]. The HMBC correlations from protons H_6_ (2 × OMe) at *δ* 3.71 (6H, s) to carbons C-3 and C-5 (*δ* 162.2); proton H-1″ at *δ* 4.83 (1H, d, *J* = 7.3 Hz) correlated with C-4′ (*δ* 157.4); the correlations from H-1‴ at *δ* 4.93 (1H, d, *J* = 1.5 Hz) to C-6′′ (*δ* 68.2) and from H-6a′′ at *δ* 3.60 (1H, m) and H-6b′′ at *δ* 4.05 (1H, m) to C-1‴ (*δ* 110.0) were further confirmed that the methoxyl moiety, *β*-d-glucopyranose and *α*-l-arabinofuranose were located at C-3/C-5, C-4′ and glc-C-6″, respectively (Fig. 2). In addition, the monosaccharides of **1** were further confirmed to be d-glucopyranose and l-arabinofuranose via the RP-HPLC analysis after acid hydrolysis and derivatization of **1**, with the retention time (*t*_R_) were 35.087 min and 37.319 min for d-glucopyranose and l-arabinofuranose (Table S1). As a result, compound **1** was conclusively identified as 3,5-dimethoxydihydrostilbene 4′-*O*-*α*-l-arabinofuranosyl-(1 → 6)-*β*-d-glucopyranoside, a previously unreported compound that has been designated as oleiferaside A.

Compound **2** was achieved in the form of a white amorphous powder. The analysis of HR-ESI-MS data revealed the molecular formula of C_27_H_36_O_12_ for compound **2**, indicating the presence of ten degrees of unsaturation. Comparing the ^1^H and ^13^C NMR spectra of compounds **1** and **2**, it was evident that they shared a high degree of similarity. The main difference observed was the presence of a *α*-l-arabinopyranose [*δ*_H_ 4.30 (1H, d, *J* = 6.8 Hz, arap-H-1‴)/*δ*_C_ 104.9, 72.5, 74.1, 69.5, 66.7 (arap-C-1‴-5‴)] in **2**, instead of a *α*-l-arabinofuranose [*δ*_H_ 4.93 (1H, d, *J* = 1.5 Hz, araf-H-1‴)/*δ*_C_ 110.0, 83.3, 78.9, 85.8, 63.0 (araf-C-1‴-5‴)] in **1** (see Table 1). In addition, the assignment of the sugar chain (6-*O*-*α*-l-arabinopyranosyl-*β*-d-glucopyranoside) was accomplished by comparing its spectral data with those of the known compound 2-phenylethyl 6-*O*-*α*-l-arabinopyranosyl- *β*-d-glucopyranoside and relevant literature values [23]. These observations suggested that the relative configurations of glucopyranose and arabinopyranose in **2** were *β*-positioned and *α*-positioned. Also, the *β*-glucopyranose and *α*-arabinopyranose located at C-4′ and glc-C-6″ were further confirmed by HMBC spectrum (Fig. 2). Moreover, RP-HPLC analysis further confirmed that the monosaccharides of **2** are d-glucopyranose and l-arabinopyranose, which were obtained through acid hydrolysis of **2** with 2 M HCl, with the *t*_R_ were 35.087 min and 37.011 min for d-glucopyranose and l-arabinopyranose (Table S1). As a result, compound **2** was conclusively identified as 3,5-dimethoxydihydrostilbene 4′-*O*-*α*-l-arabinopyranosyl- (1 → 6)-*β*-d-glucopyranoside, a previously unreported compound that has been designated as oleiferaside B.

Compound **3** was gained in the form of a white amorphous powder. The analysis of HR-ESI-MS data revealed the molecular formula of C_27_H_36_O_12_ for compound **3**, indicating the presence of ten degrees of unsaturation. The ^1^H and ^13^C NMR spectra showed a high degree of similarity between compounds **1** (or **2**) and **3**, with the main distinguishing feature being the presence of a terminal connected sugar moiety *β*-d-apiofuranose [*δ*_H_ 4.98 (1H, d, *J* = 2.4 Hz, apif-H-1‴)/*δ*_C_ 111.1, 78.0, 80.5, 75.0, 65.5 (apif-C-1‴-5‴)] in **3**, instead of a *α*-l-arabinofuranose or *α*-l-arabinopyranose in **1** or **2** (see Table 1). Furthermore, the sugar chain assignment (6-*O*-*β*-d-apiofuranosyl-*β*-d-glucopyranoside) was determined by comparing its NMR spectral data with a known compound, 2-phenylethyl 6-*O*-*β*-d- apiofuranosyl-*β*-d-glucopyranoside, using *δ* values for glc and apif, as well as references from literature [23,24]. Based on these observations, it can be inferred that the relative configurations of glucopyranose and apiofuranose in compound **3** are both located in the *β* position. Additionally, the HMBC spectrum further confirmed the presence of *β*-glucopyranose at C-4′ and *β*-apiofuranose at glc-C-6''. (Fig. 2). Moreover, the monosaccharides in compound **3** were further identified as d-glucopyranose and d-apiofuranose through the RP-HPLC analysis following acid hydrolysis of **3** using 2 M HCl and derivatization of sugars, with the *t*_R_ were 35.087 min and 44.219 min for d-glucopyranose and d-apiofuranose (Table S1). As a result, compound **3** was conclusively identified as 3,5-dimethoxydihydrostilbene 4′-*O*-*β*-d-apiofuranosyl-(1 → 6)-*β*-d- glucopyranoside, a previously unreported compound that has been designated as oleiferaside C.

Nine previously described compounds **4–12** were determined to be 1-(3′,5′-dimethoxy) phenyl-2-[4″-*O*-*β*-d-glucopyranosyl(6 → 1)-*O*-*α*-l-rhamnopyranosyl]phenylethane (**4**) [7], sasastilboside A (**5**) [25], 3,5-dihydroxydihydrostilbene 4′-*O*-*β*-d-glucopyranoside (**6**) [7], 5,4′-dihydroxy-dihydrostilbene 3-*O*-*β*-d-glucopyranoside (**7**) [26], 3,5-dihydroxyl- dihydrostilbene 4′-*O*-[6″-*O*-(4‴-methoxylgalloyl)]-*β*-d-glucopyranoside (**8**) [12], quercitrin (**9**) [27], phlorizin (**10**) [28], quercetin-3-*O*-*β*-d-galactopyranoside (**11**) [9], and quercetin-3-*O*-*β*-d-glucopyranoside (**12**) [9] via comparing the NMR and mass data of the compounds with the values reported in the literature.

**Fig. 3:**
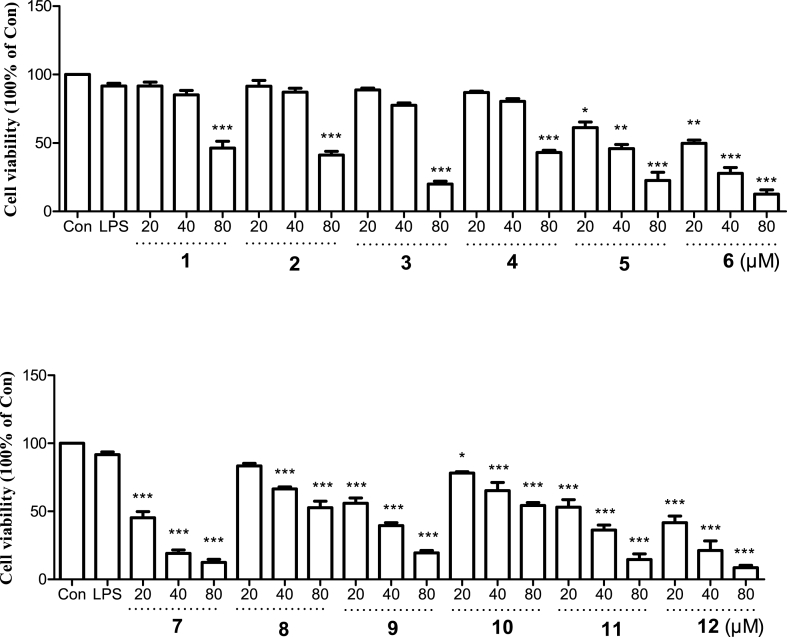
Cell viability of compounds **1**–**12** on LPS-induced RAW264.7 cells. The values are expressed as mean of three experiments ±S.D. *p < 0.05, **p < 0.01 and ***p < 0.001 compared with Con group.

To assess the anti-inflammatory activities of the isolates, LPS-induced RAW264.7 cells were utilized. Prior to conducting the experiments, the cytotoxicity of all compounds was tested up to a concentration of 80 μM using the MTT assay. We found that 20 μM and 40 μM of tested compounds **1**–**4** showed very weak impact on cell viability of RAW264.7 macrophages, while 80 μM of all compounds exhibited significant cytotoxicity (Fig. 3). As shown in Table 2, the results of nitrite and PGE_2_ assay demonstrated that the IC_50_ values of NO and PGE_2_ for compounds **1**–**4** were 28.56 ± 0.16 and 15.14 ± 0.11 (**1**), 42.21 ± 0.21 and 20.22 ± 0.18 (**2**), 37.88 ± 0.32 and 12.25 ± 0.24 (**3**), 23.47 ± 0.15 and 13.31 ± 0.22 (**4**), respectively. These findings uncovered the potential anti-inflammatory activity of those secondary metabolites. Additional investigations are necessary to assess the mechanism of anti-inflammation for active phytochemical constituents.

**Table 2:** Effects of 4 selected natural products on production of LPS-stimulated NO and PGE_2_ in RAW264.7 cells.

Compounds	NO (IC_50_, μM)	PGE_2_ (IC_50_, μM)
**1**	28.56 ± 0.16	15.14 ± 0.11
**2**	42.21 ± 0.21	20.22 ± 0.18
**3**	37.88 ± 0.32	12.25 ± 0.24
**4**	23.47 ± 0.15	13.31 ± 0.22
Butein	5.56 ± 0.13	8.79 ± 0.17

Butein: positive control. The data is presented as mean ± SD based on triple separate experiments.

## 4 Conclusions

To sum up, the research on the chemical constituents of *C. oleifera* leaves resulted in identifying twelve different compounds. Among them, three undescribed compounds named oleiferaside A (**1**), oleiferaside B (**2**), and oleiferaside C (**3**) were identified, along with nine previously reported natural products (**4–12**). Additionally, the toxicity of the separated substances was evaluated, along with their ability to inhibit the generation of NO and PGE_2_ caused by LPS in RAW264.7 macrophages. The tested dihydrostilbene glycosides (**1–4**) demonstrated moderate anti-inflammatory activities without causing any cytotoxic effects.

## Data availability statement

Data included in article/supp. material/referenced in article.

## CRediT authorship contribution statement

**Yi Xu:** Writing – original draft, Investigation. **Si-Qi Tang:** Writing – original draft, Methodology, Investigation. **Zong-Wu Suo:** Formal analysis. **Kai-Xin Wei:** Formal analysis. **Walter Luyten:** Validation. **Hao Huang:** Validation, Supervision, Resources. **Xiao-Jun Li:** Writing – review & editing, Supervision, Project administration, Funding acquisition, Conceptualization.

## Declaration of competing interest

The authors declare no conflict of interest.
