# Supplementary material for: Three undescribed dihydrostilbene glycosides from leaves of Camellia oleifera Abel. And their anti-inflammatory activity
Source: Heliyon. 2024 Apr 30;10(9):e30507. doi: 10.1016/j.heliyon.2024.e30507 (PMC11088326; doi:10.1016/j.heliyon.2024.e30507)
Supplement: Multimedia component 1 [file mmc1.docx]

**Supplementary Materials**

**Three undescribed dihydrostilbene glycosides from leaves of *Camellia oleifera* Abel. and their anti-inflammatory activity**

Yi Xu^a,b,#^, Si-Qi Tang^a,#^, Zong-Wu Suo^a^, Kai-Xin Wei^a^, Walter Luyten^c^, Hao Huang^a,*^, Xiao-Jun Li^a,*^

*^a^ National Engineering Research Center for Modernization of Traditional Chinese Medicine - Hakka Medical Resources Branch, School of Pharmacy, Gannan Medical University, Ganzhou 341000, China*

*^b^ First Affiliated Hospital of Gannan Medical University, Ganzhou 341000, China*

*^c^ Department of Biology, KU Leuven, Leuven, Belgium*

^#^ These authors contribute equally.

**Corresponding author:**

Xiao-Jun Li

School of Pharmacy, Gannan Medical University, Ganzhou, Jiangxi 341000, China.

E-mail: [xjli@gmu.edu.cn](mailto:xjli@gmu.edu.cn)

Tel.: +86 797-816-9805

Hao Huang

School of Pharmacy, Gannan Medical University, Ganzhou, Jiangxi 341000, China.

E-mail: [huanghao_26@163.com](mailto:huanghao_26@163.com)

Tel.: +86 797-816-9805

**Abstract**: Three previously unidentified dihydrostilbene glycosides, named oleiferaside A (**1**), oleiferaside B (**2**), and oleiferaside C (**3**), were discovered through a phytochemical exploration on *Camellia oleifera* Abel. leaves. Additionally, nine known secondary metabolites (**4-12**) were also identified. Undescribed secondary metabolites **1**-**3** were elucidated as 3,5-dimethoxydihydrostilbene 4′-*O*-*α*-L-arabinofuranosyl-(1→6)-*β*-D-glucopyranoside, 3,5-dimethoxydihydrostilbene 4′-*O*-*α*-L- arabinopyranosyl-(1→6)-*β*-D-glucopyranoside and 3,5-dimethoxydihydrostilbene 4′-*O*-*β*-D- apiofuranosyl-(1→6)-*β*-D-glucopyranoside, respectively. HR-MS and NMR spectroscopy were utilized for determining the structures of the isolates. The natural products were assessed for their anti-inflammatory effect using RAW264.7 macrophage stimulated by LPS. The findings demonstrated that compounds **1-4** exhibited inhibitory activities on NO and PGE_2_ production without causing cytotoxicity. These observations suggest that these compounds may have promising anti-inflammatory properties.

**Key words**: *Camellia oleifera* Abel.; dihydrostilbene glycosides; anti-inflammatory; oleiferaside A; oleiferaside B; oleiferaside C

**
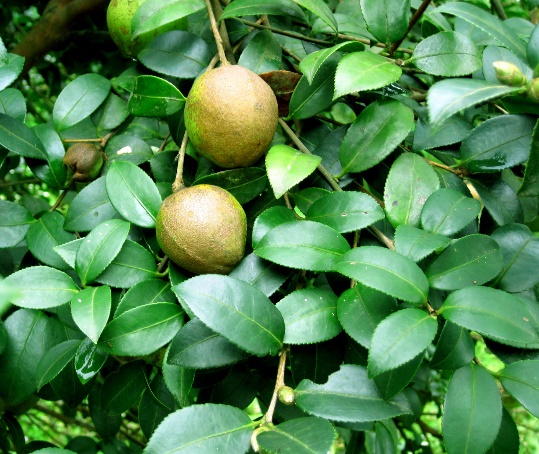

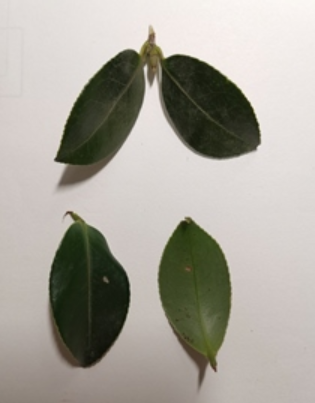
**

(a)

(b)

**Figure S1.** Photo of *Camellia oleifera* Abel. (a) and the leaves of *C. oleifera* Abel. (b) (Ganzhou City, Jiangxi Prov., by Li Xiao-jun)

**1.Spectroscopic Data of the Undescribed Compounds 1-3.**


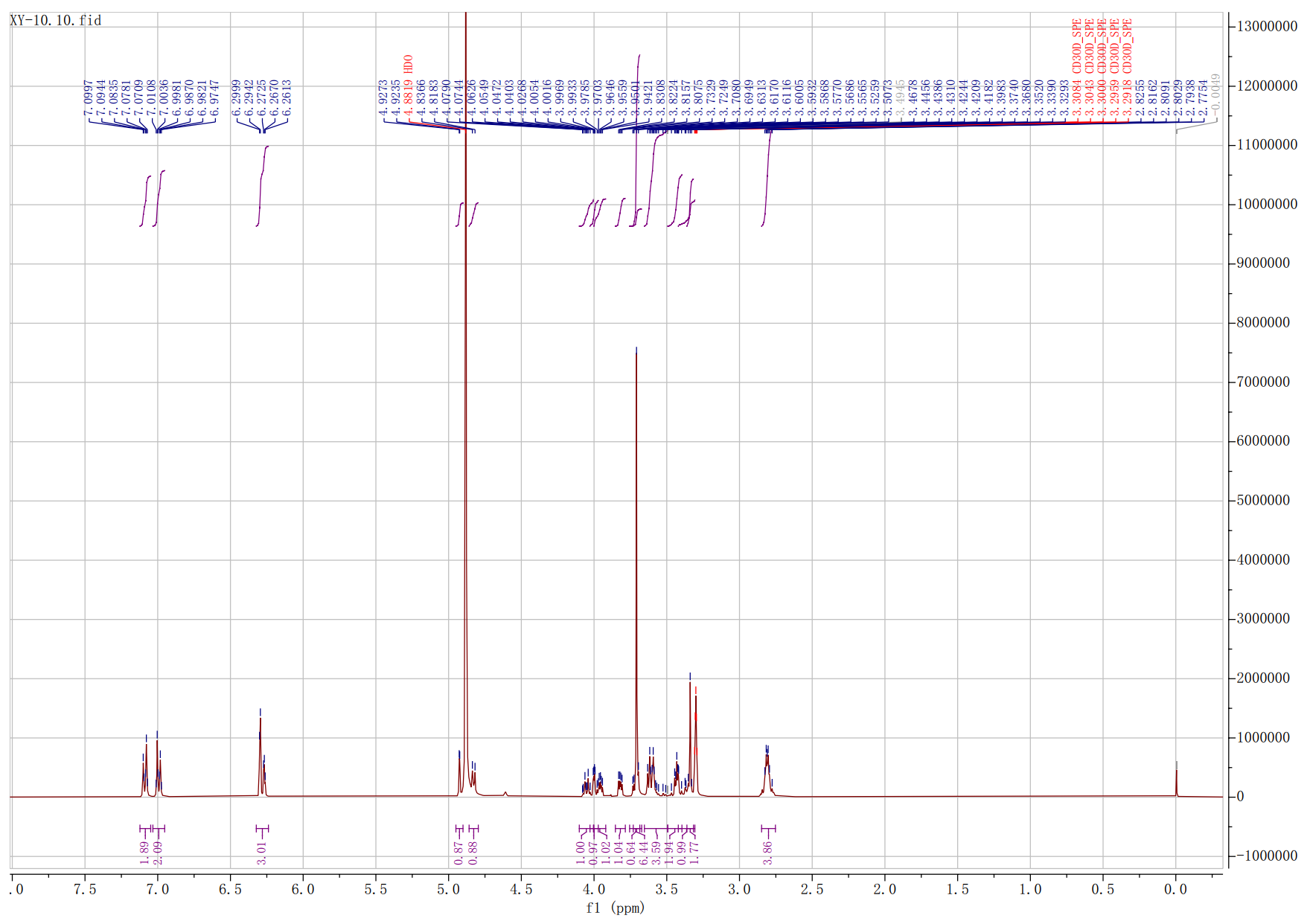


**Figure S2.** ^1^H NMR spectrum of **1** in methanol-*d_4_*


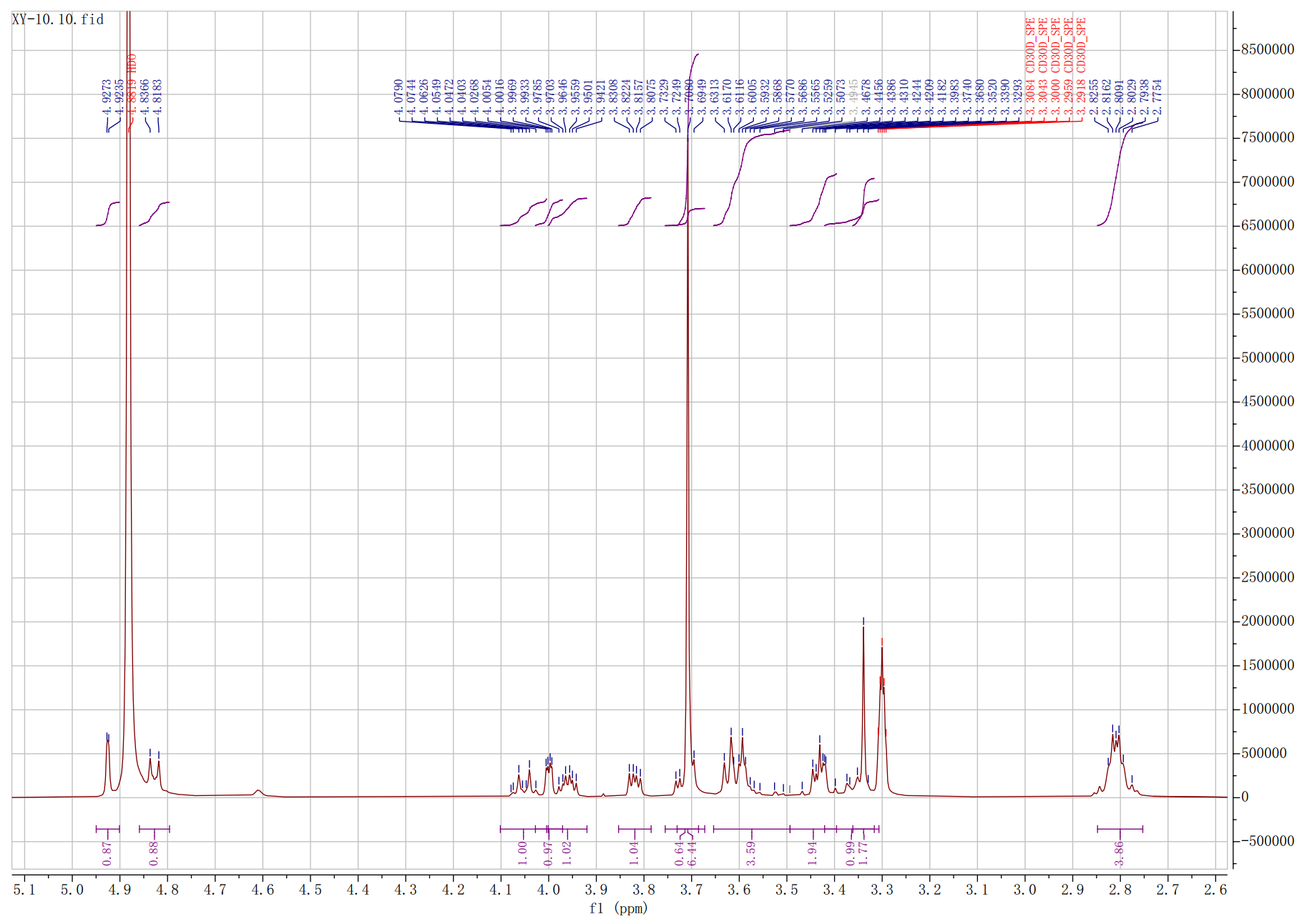


**Figure S3.** Expand ^1^H NMR spectrum of **1** in methanol-*d_4_*


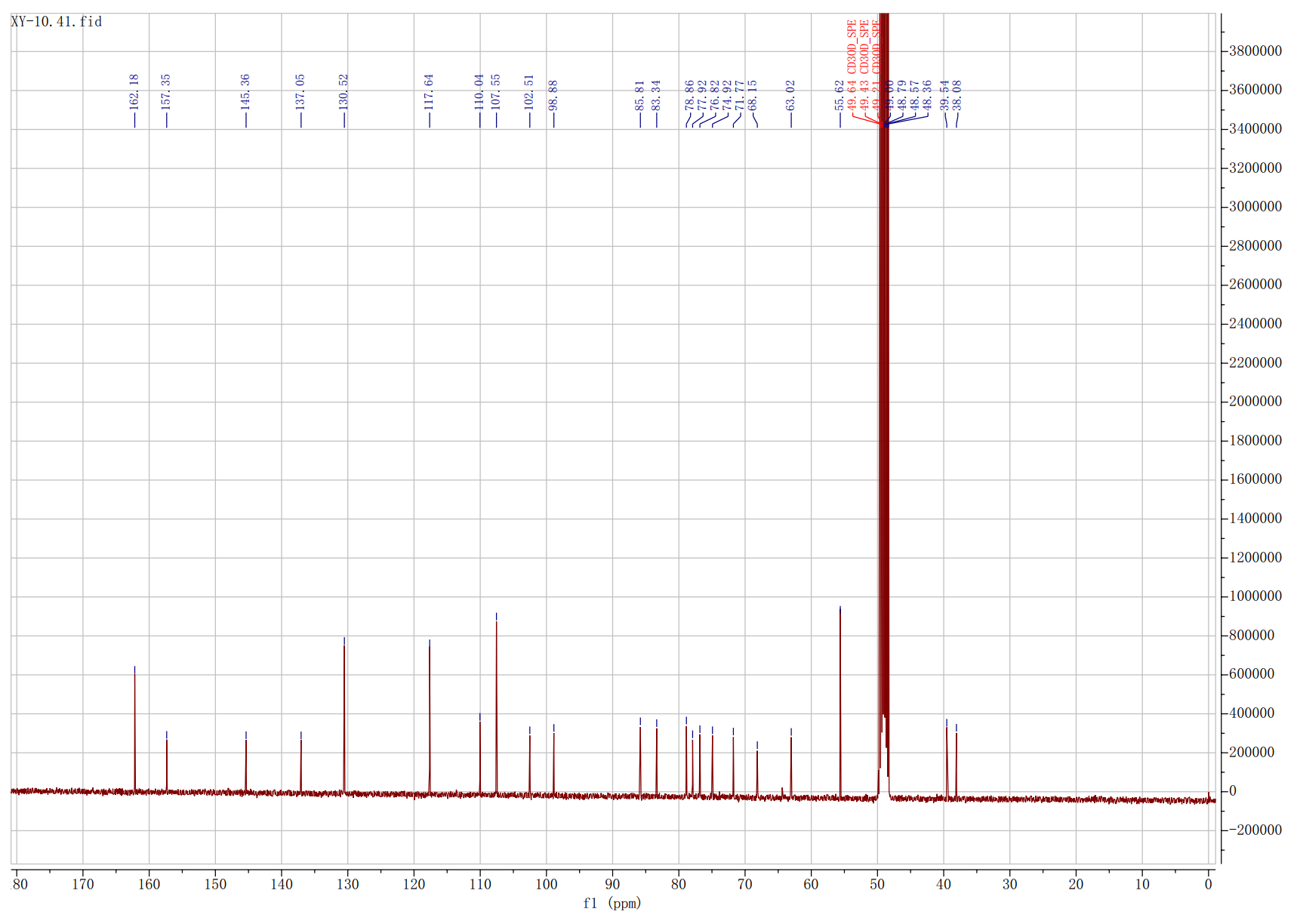


**Figure S4.** ^13^C NMR spectrum of **1** in methanol-*d_4_*


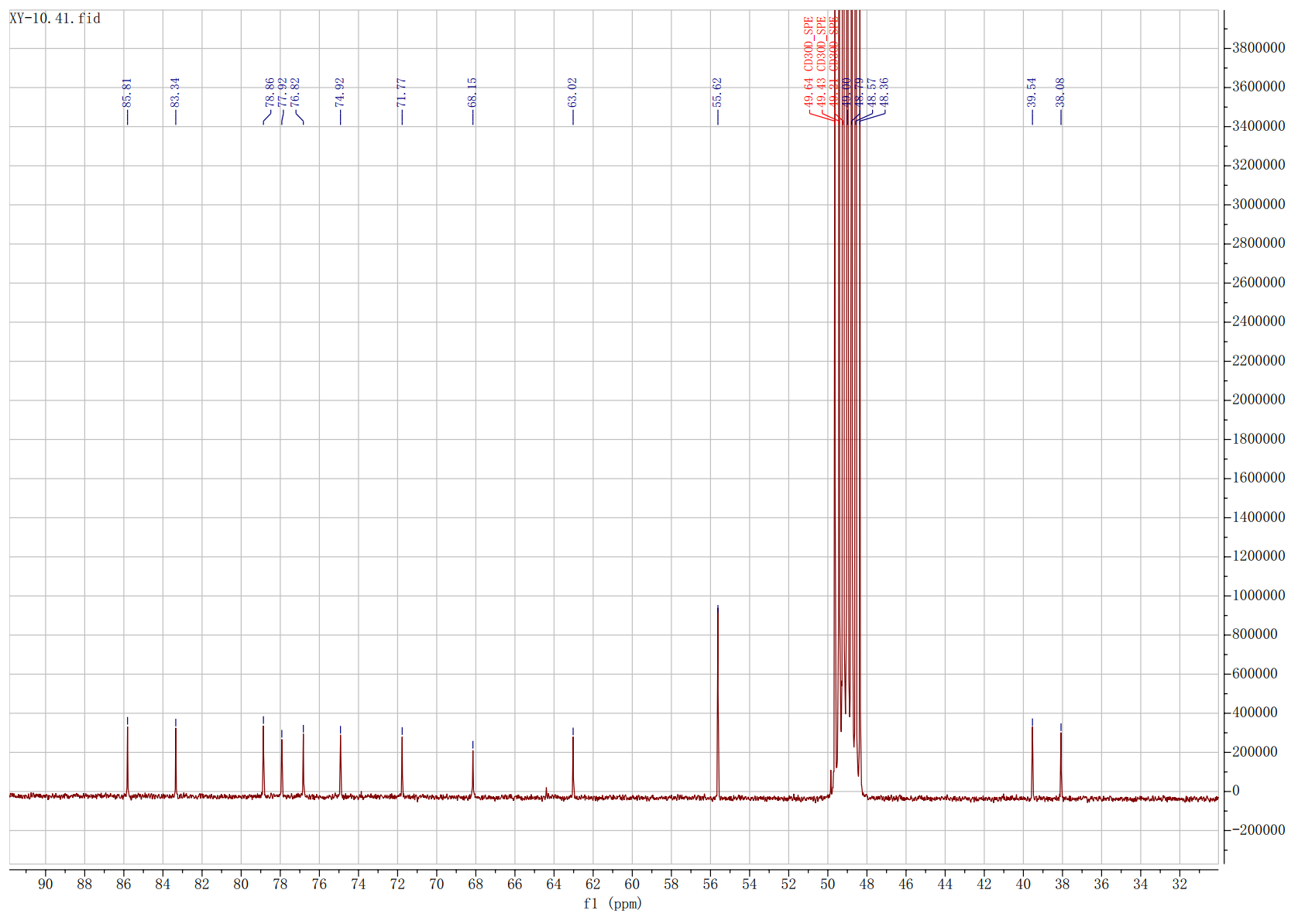


**Figure S5.** Expand ^13^C NMR spectrum of **1** in methanol-*d_4_*


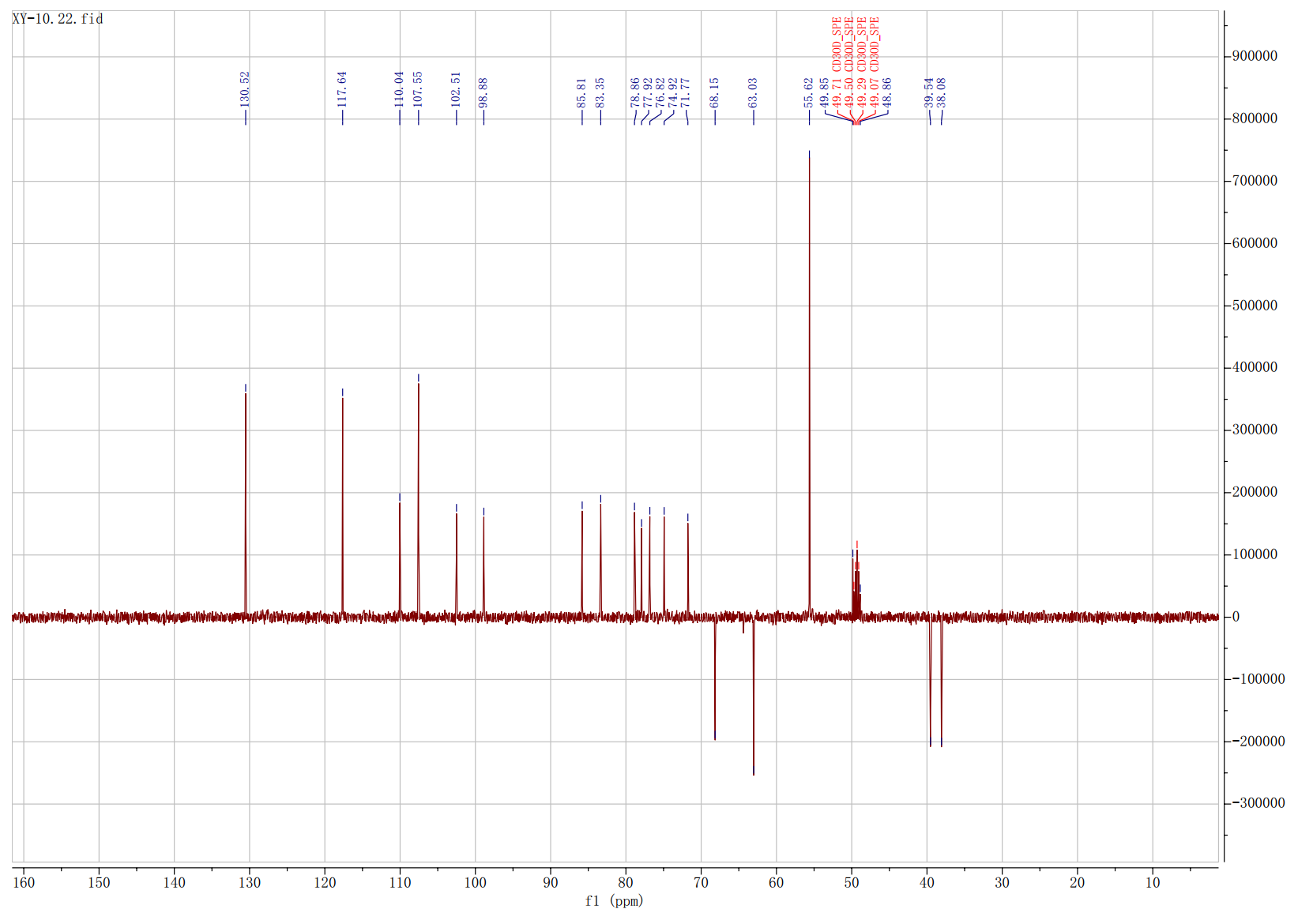


**Figure S6.** DEPT spectrum of **1** in methanol-*d_4_*


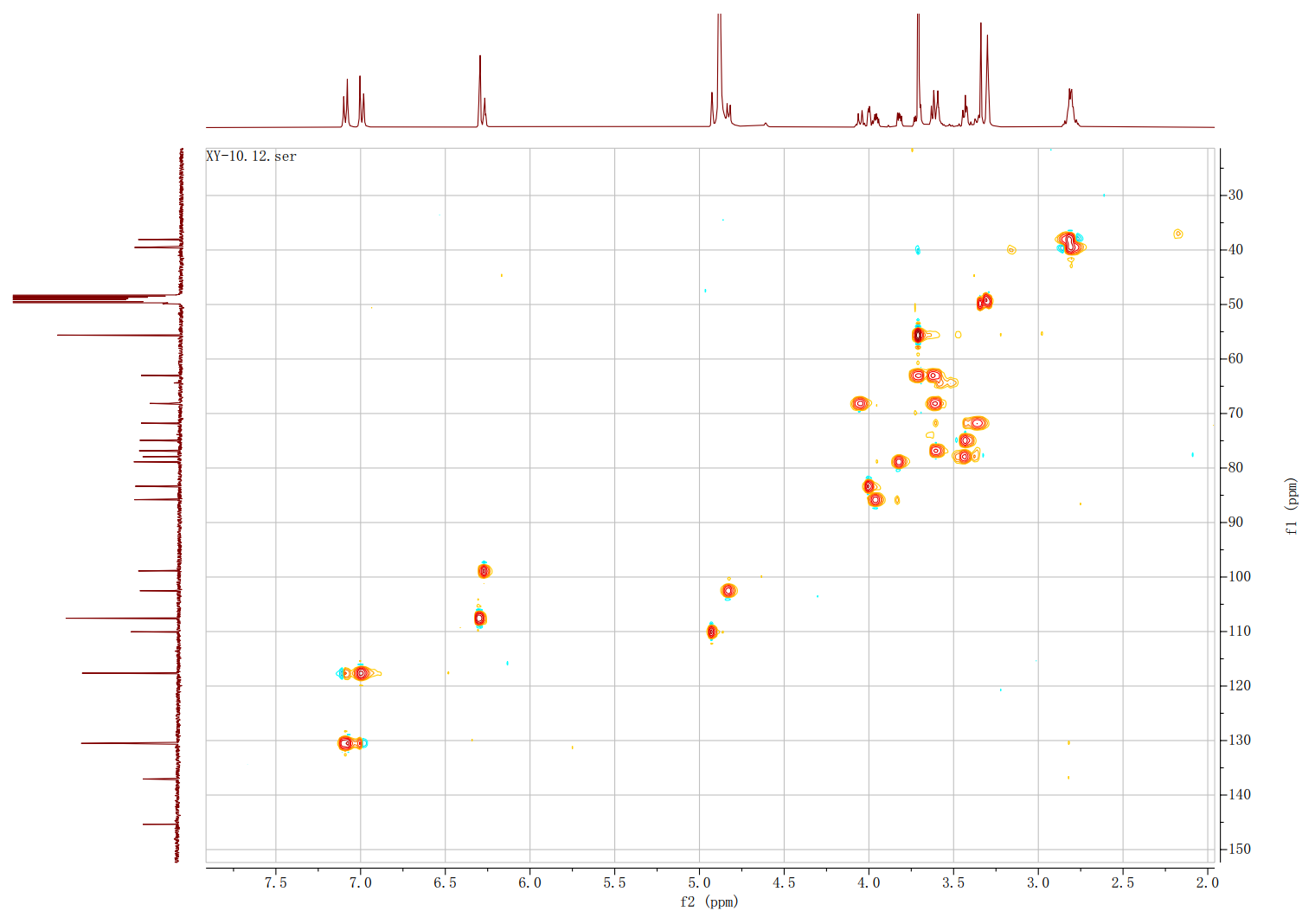


**Figure S7.** HSQC spectrum of **1** in methanol-*d_4_*


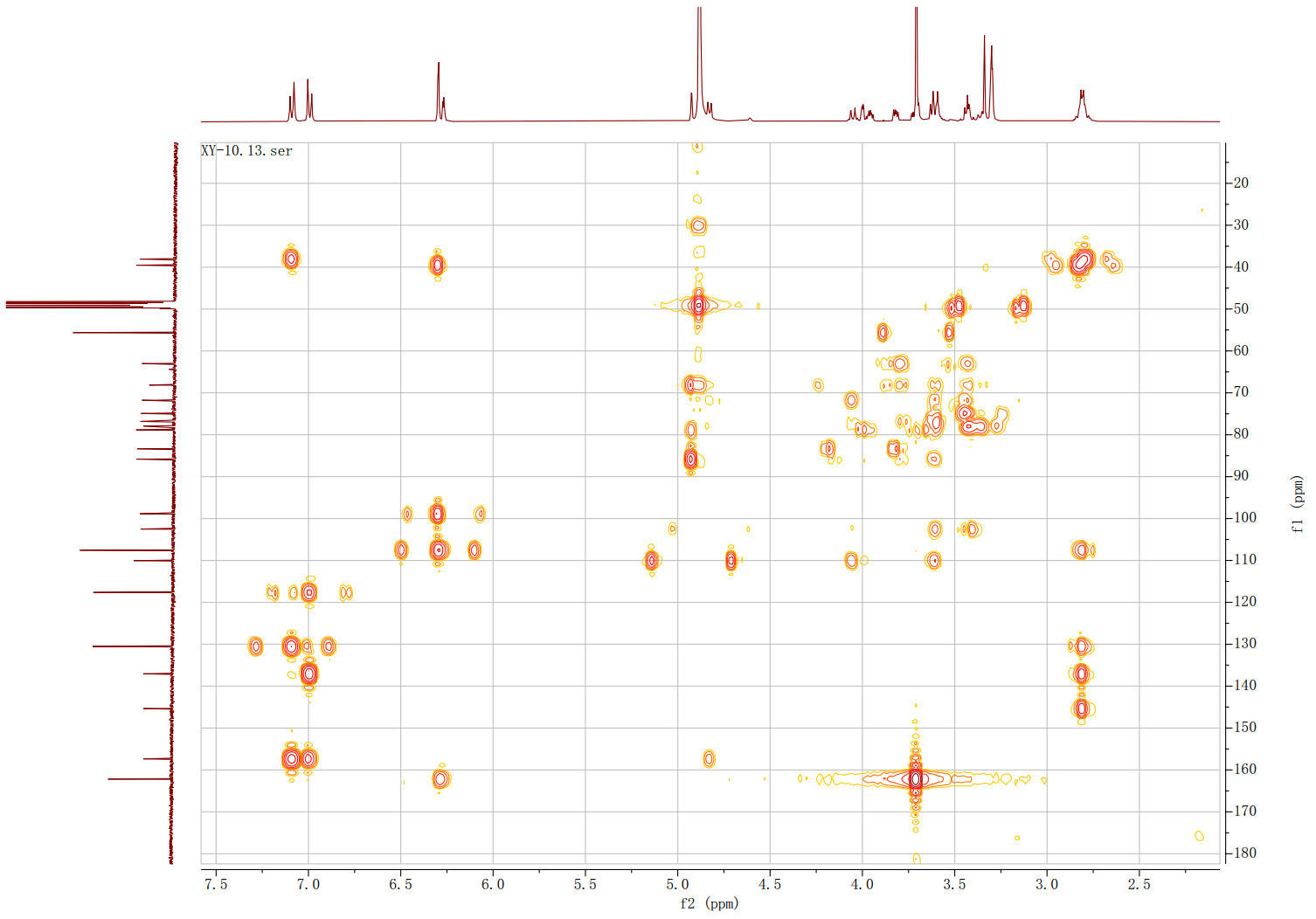


**Figure S8.** HMBC spectrum of **1** in methanol-*d_4_*


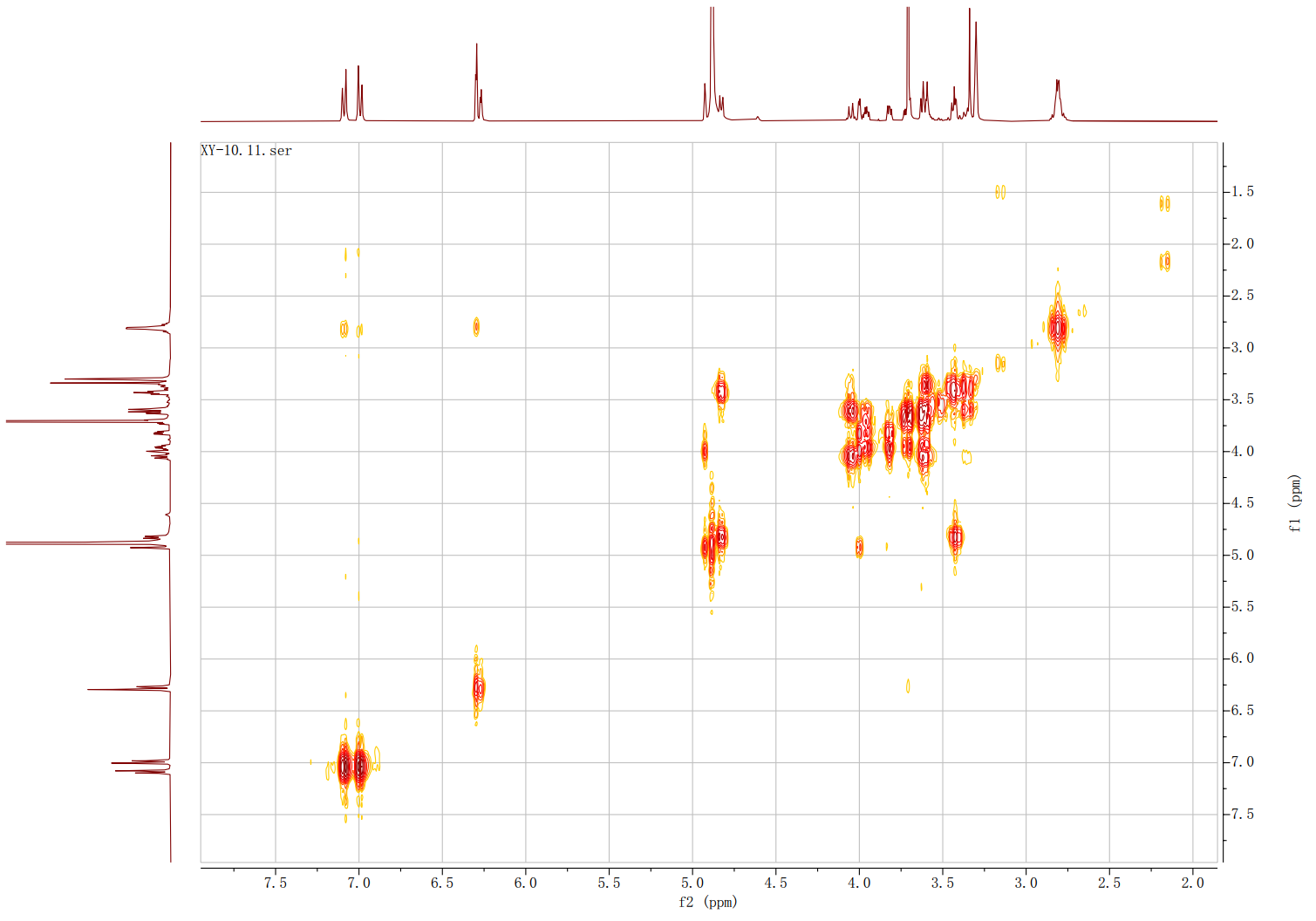


**Figure S9.** ^1^H-^1^H COSY spectrum of **1** in methanol-*d_4_*


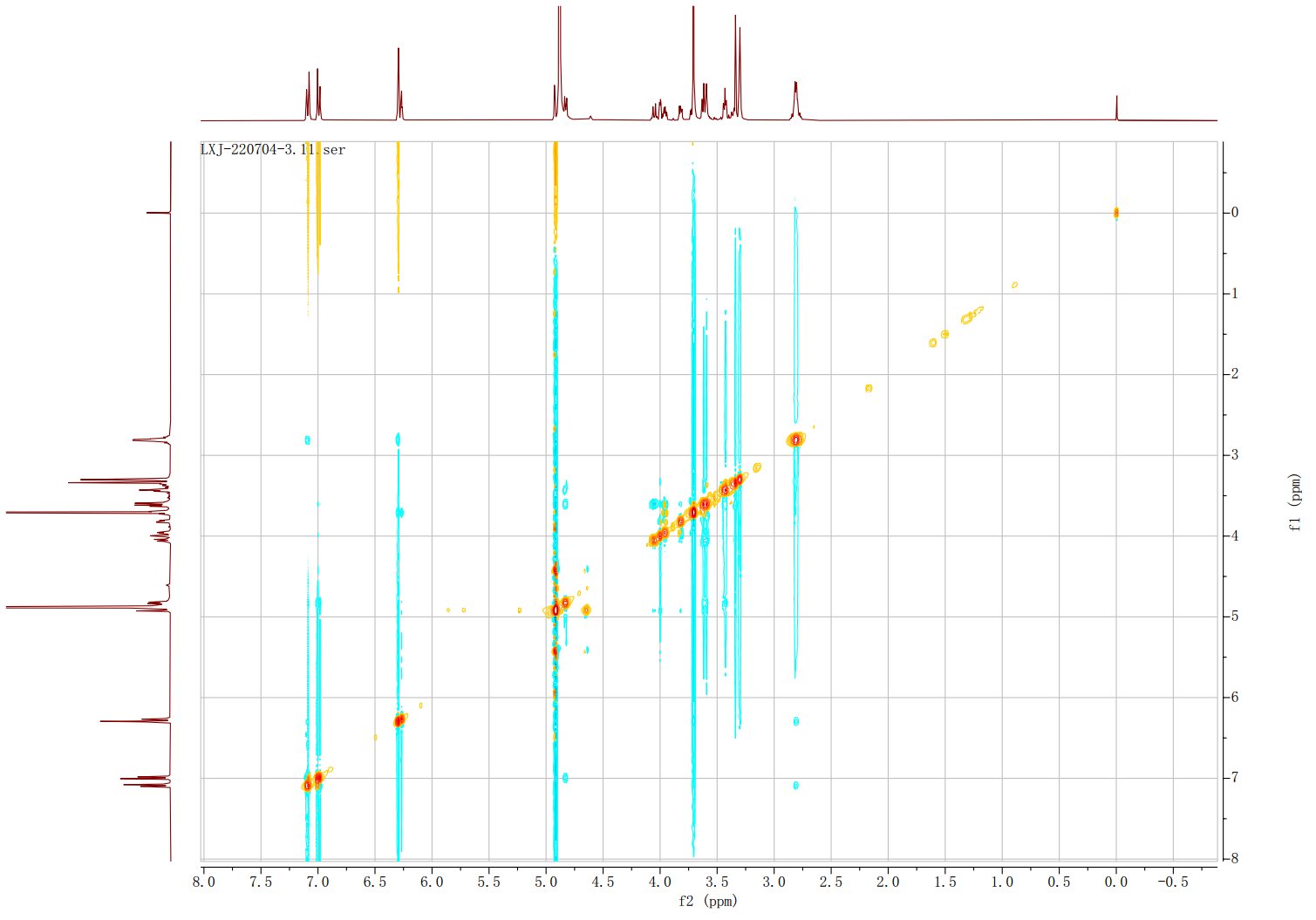


**Figure S10.** NOESY spectrum of **1** in methanol-*d_4_*


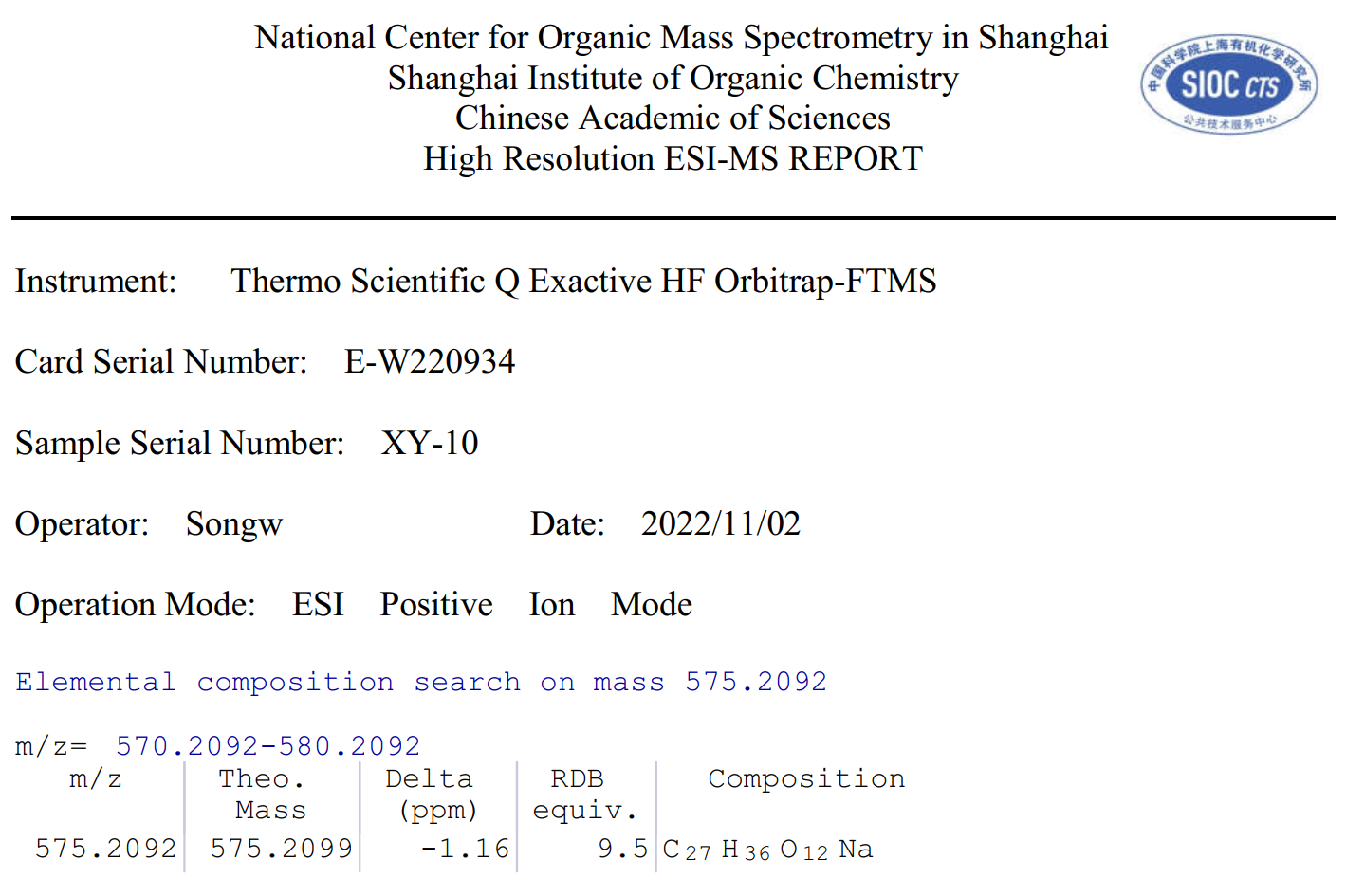


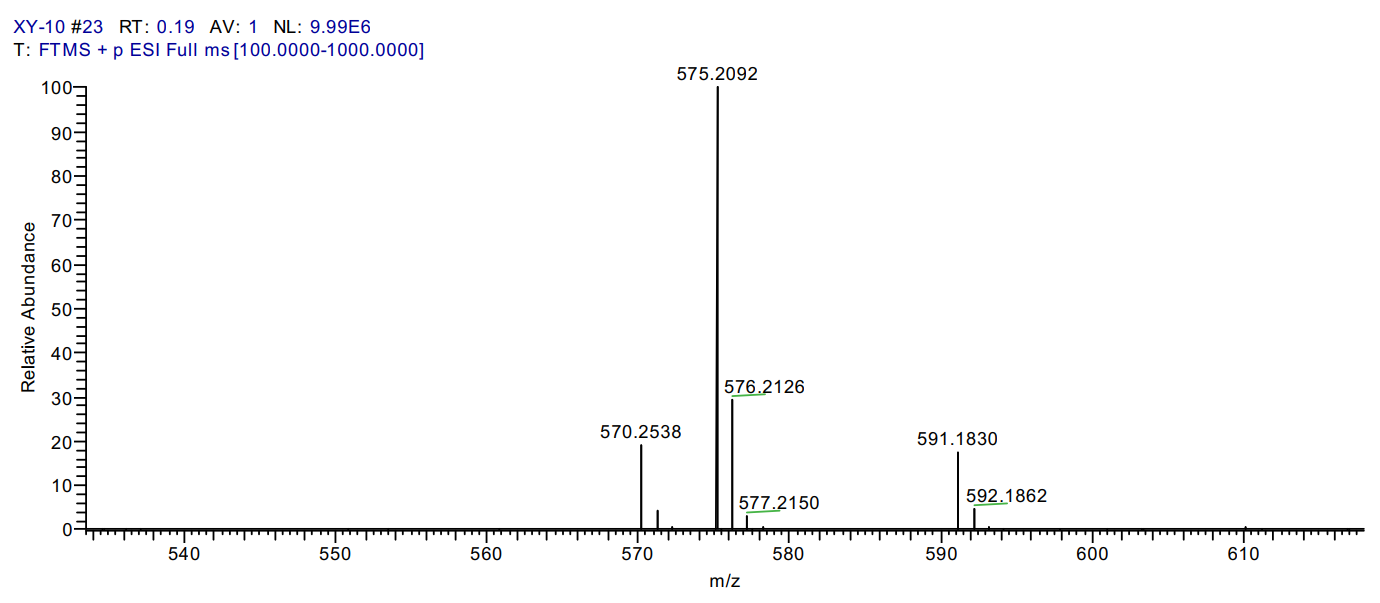


**Figure S11.** HRESIMS spectrum of **1**


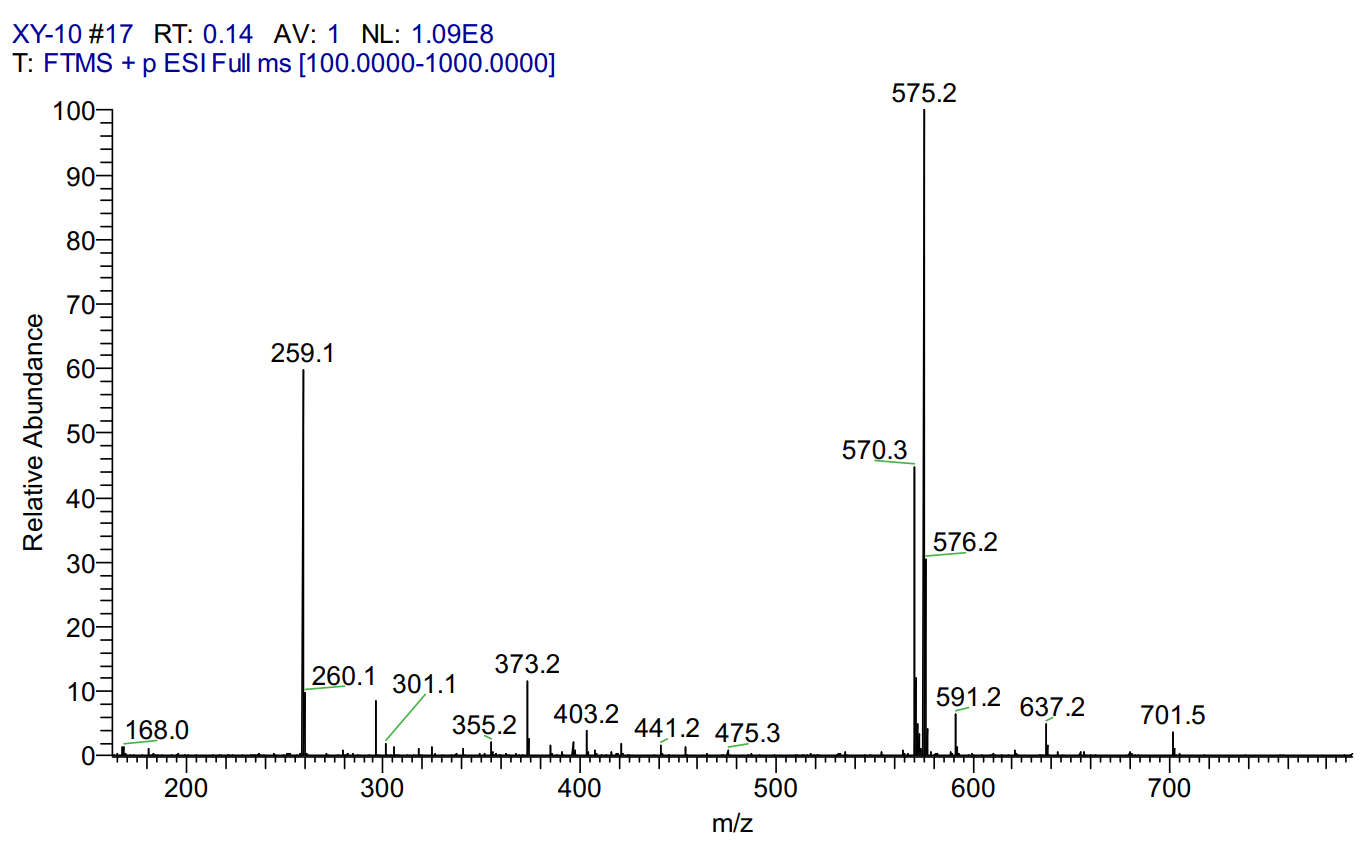


**Figure S12.** ESIMS spectrum of **1**


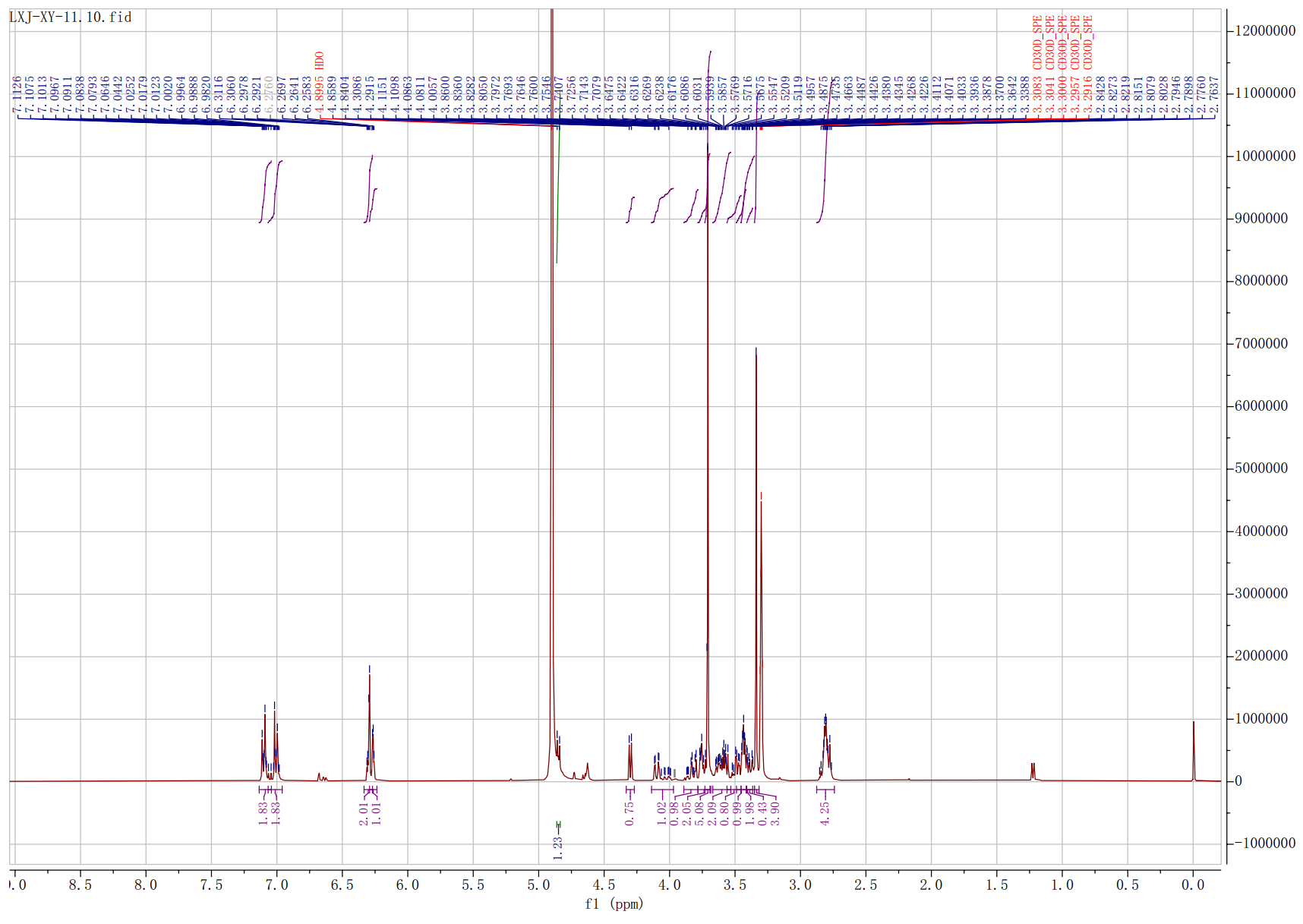


**Figure S15.** ^1^H NMR spectrum of **2** in methanol-*d_4_*


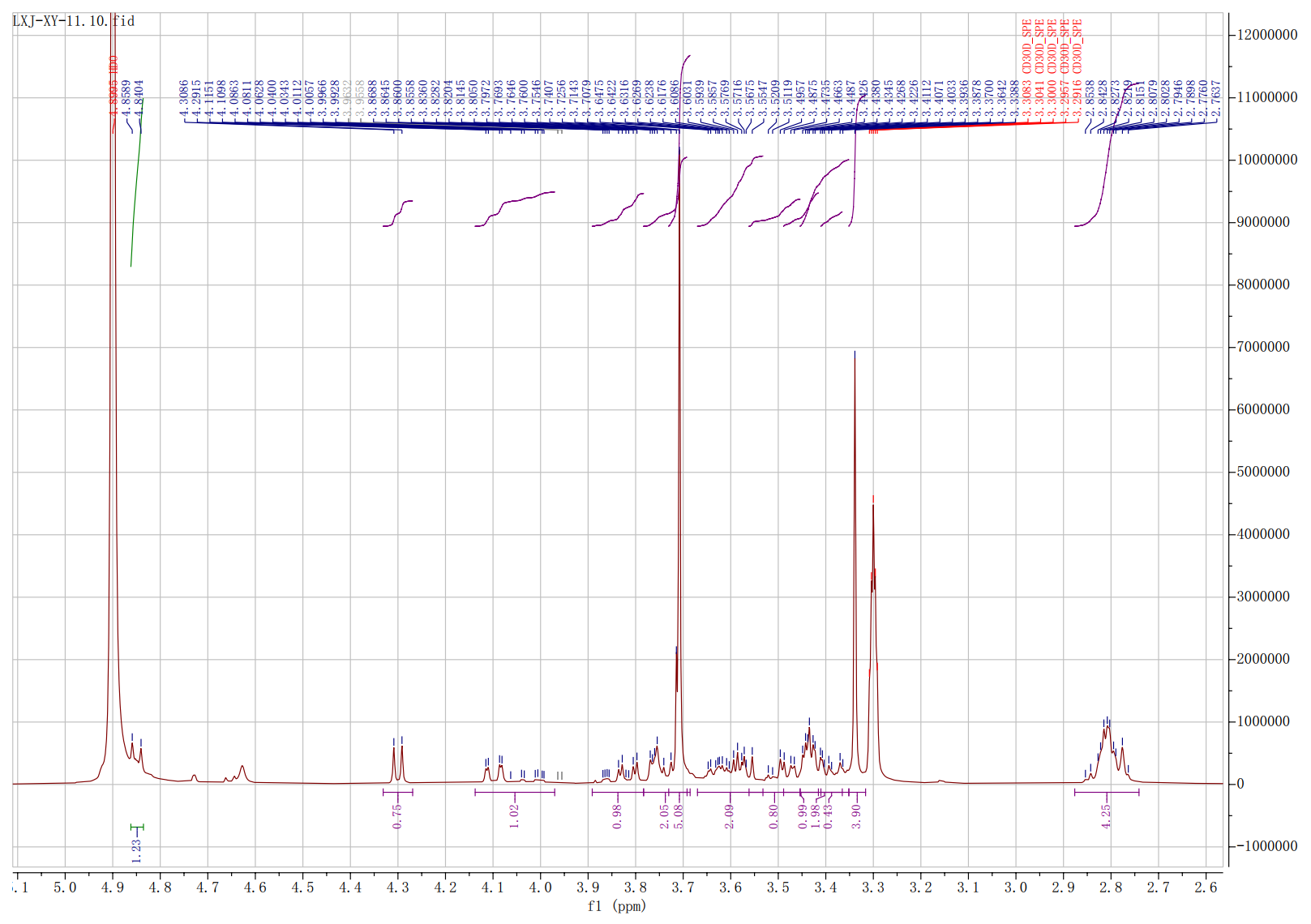


**Figure S16.** Expand ^1^H NMR spectrum of **2** in methanol-*d_4_*


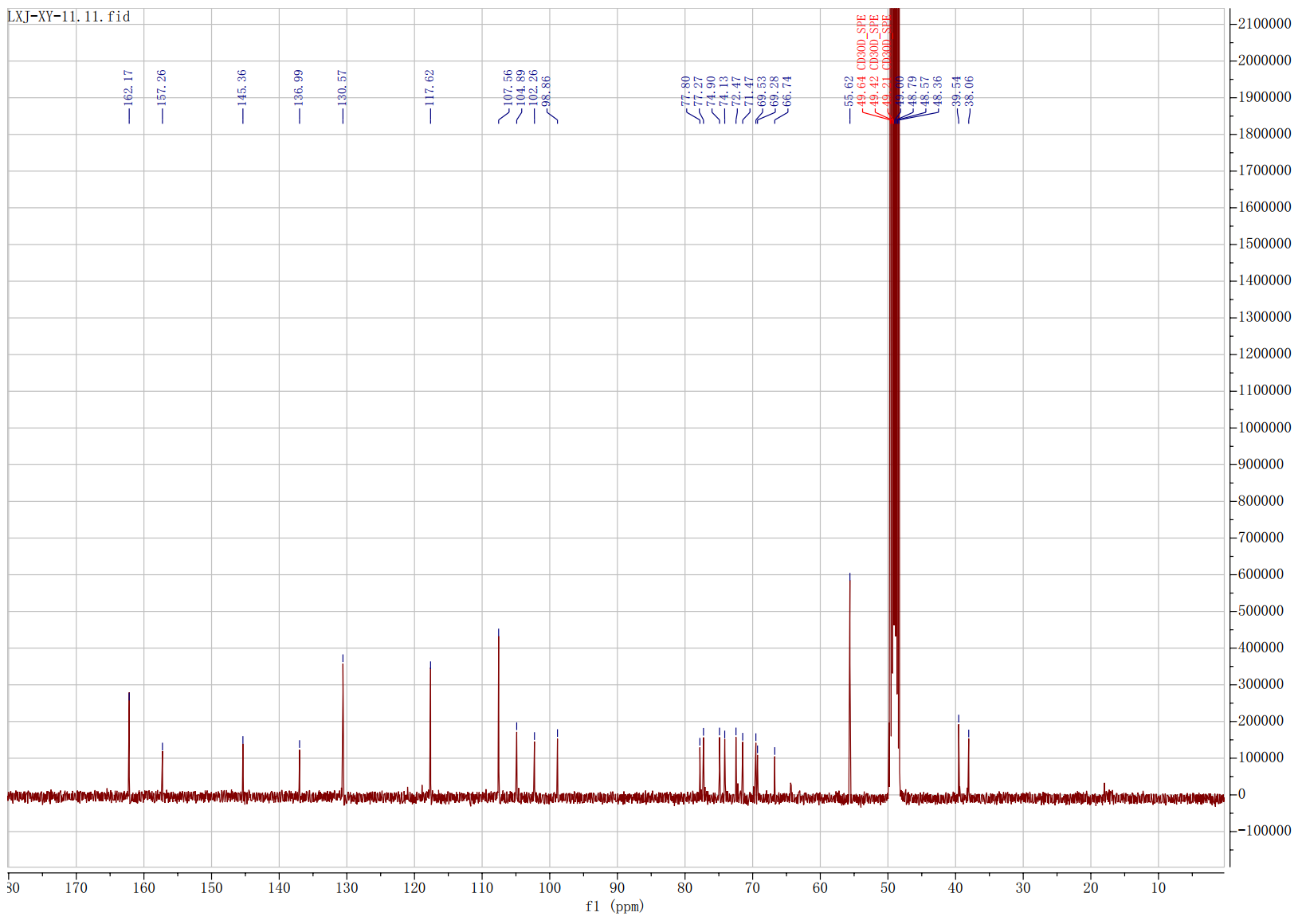


**Figure S17.** ^13^C NMR spectrum of **2** in methanol-*d_4_*


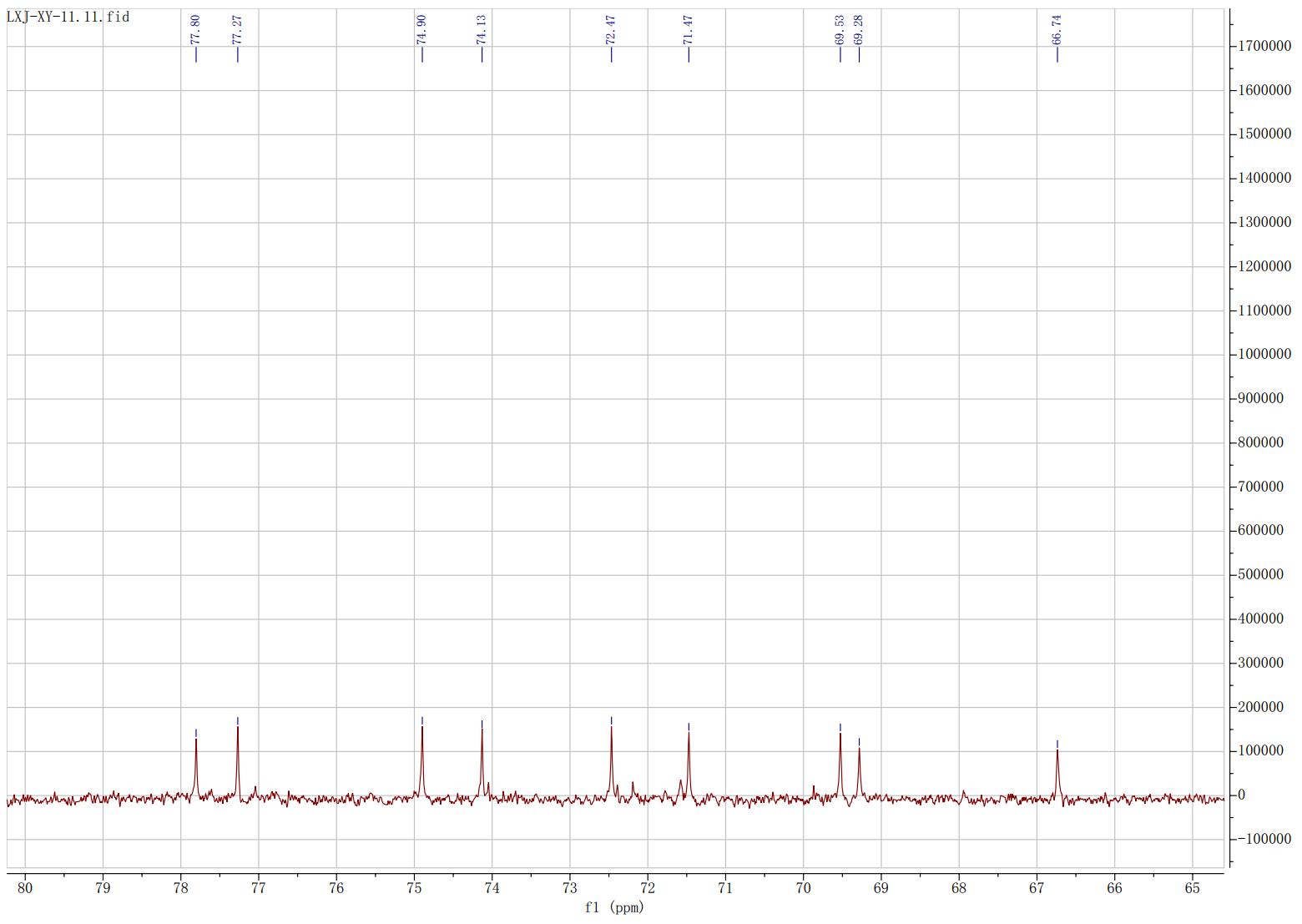


**Figure S18.** Expand ^13^C NMR spectrum of **2** in methanol-*d_4_*


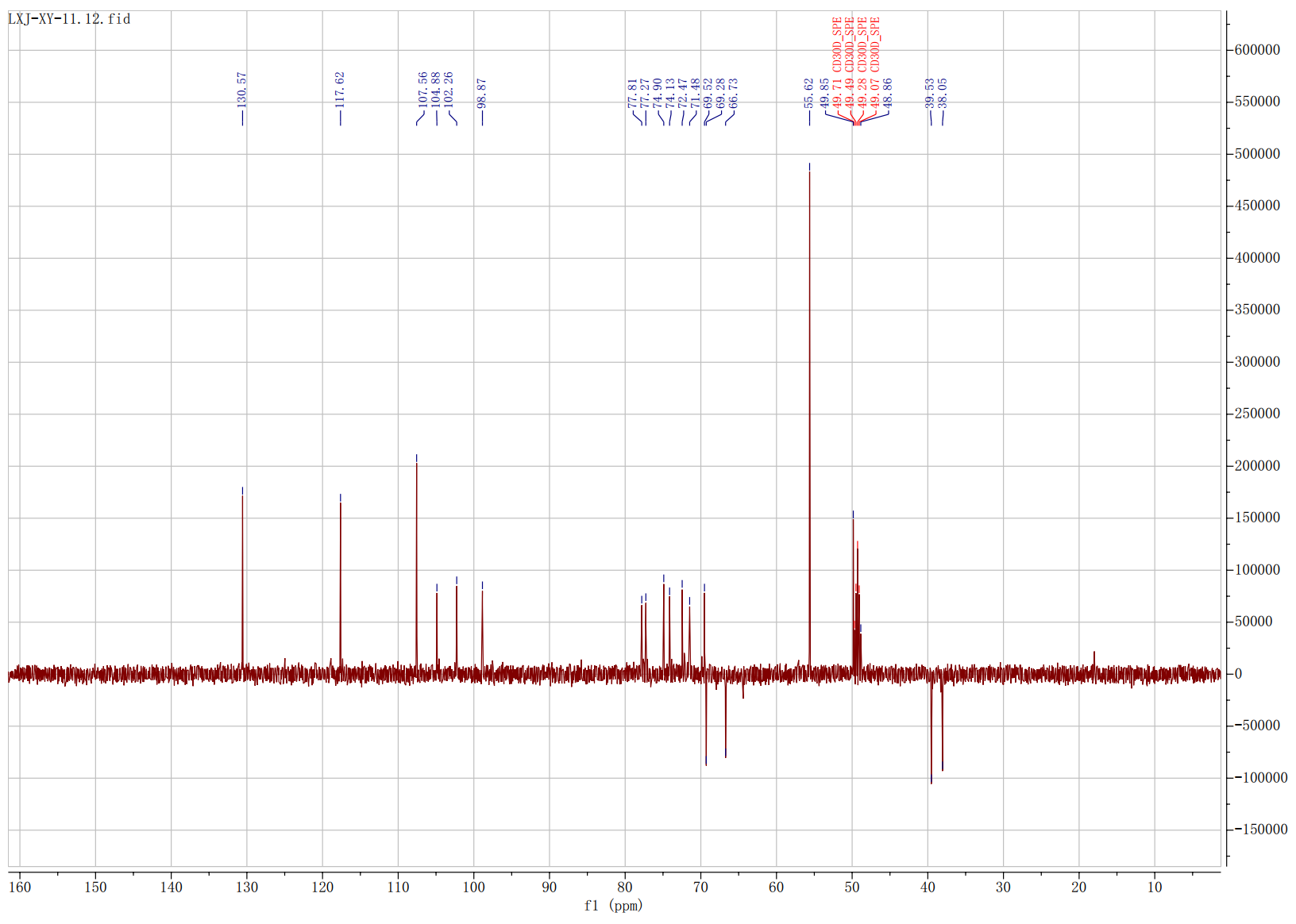


**Figure S19.** DEPT spectrum of **2** in methanol-*d_4_*


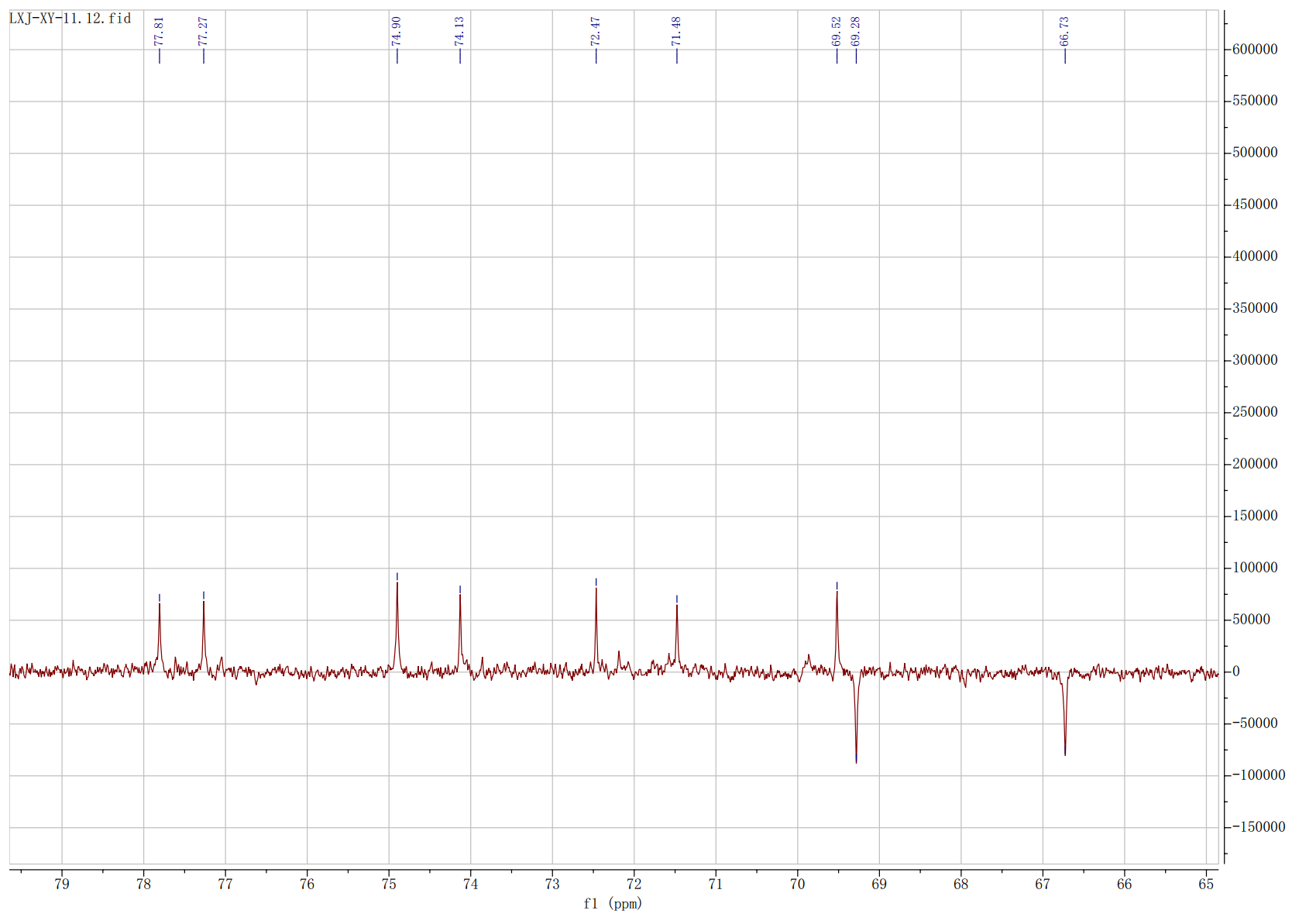


**Figure S20.** Expand DEPT spectrum of **2** in methanol-*d_4_*


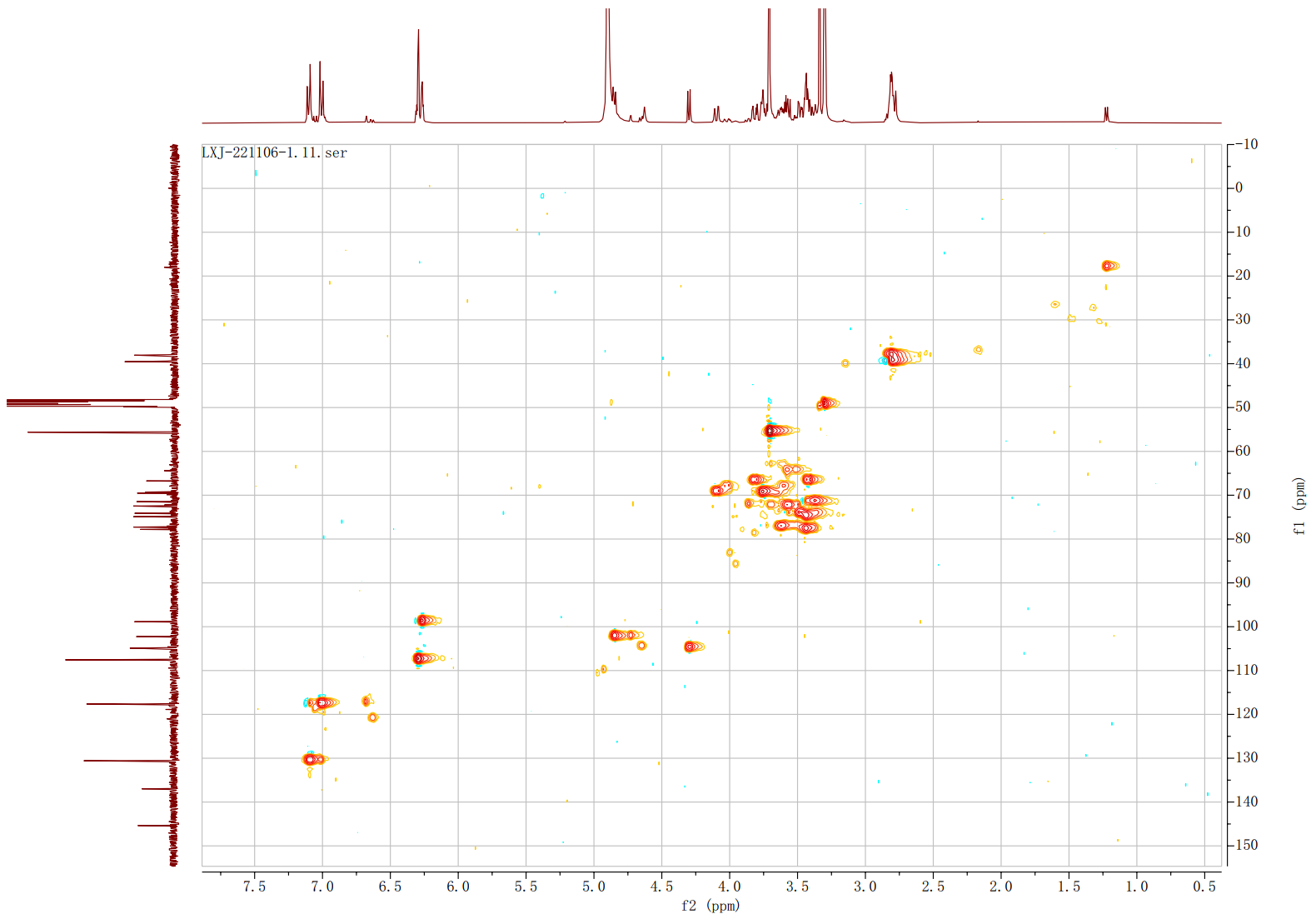


**Figure S21.** HSQC spectrum of **2** in methanol-*d_4_*


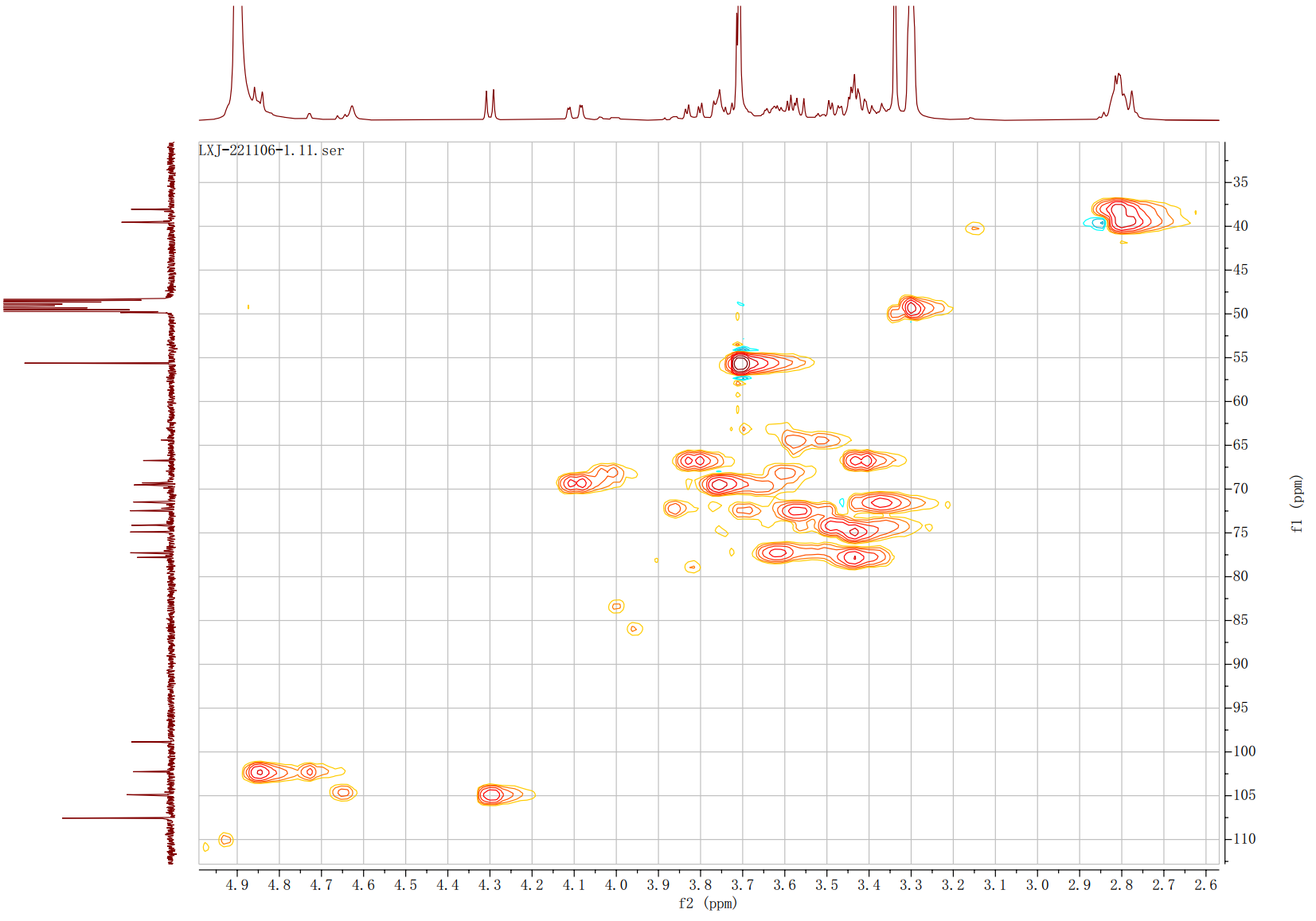


**Figure S22.** Expand HSQC spectrum of **2** in methanol-*d_4_*


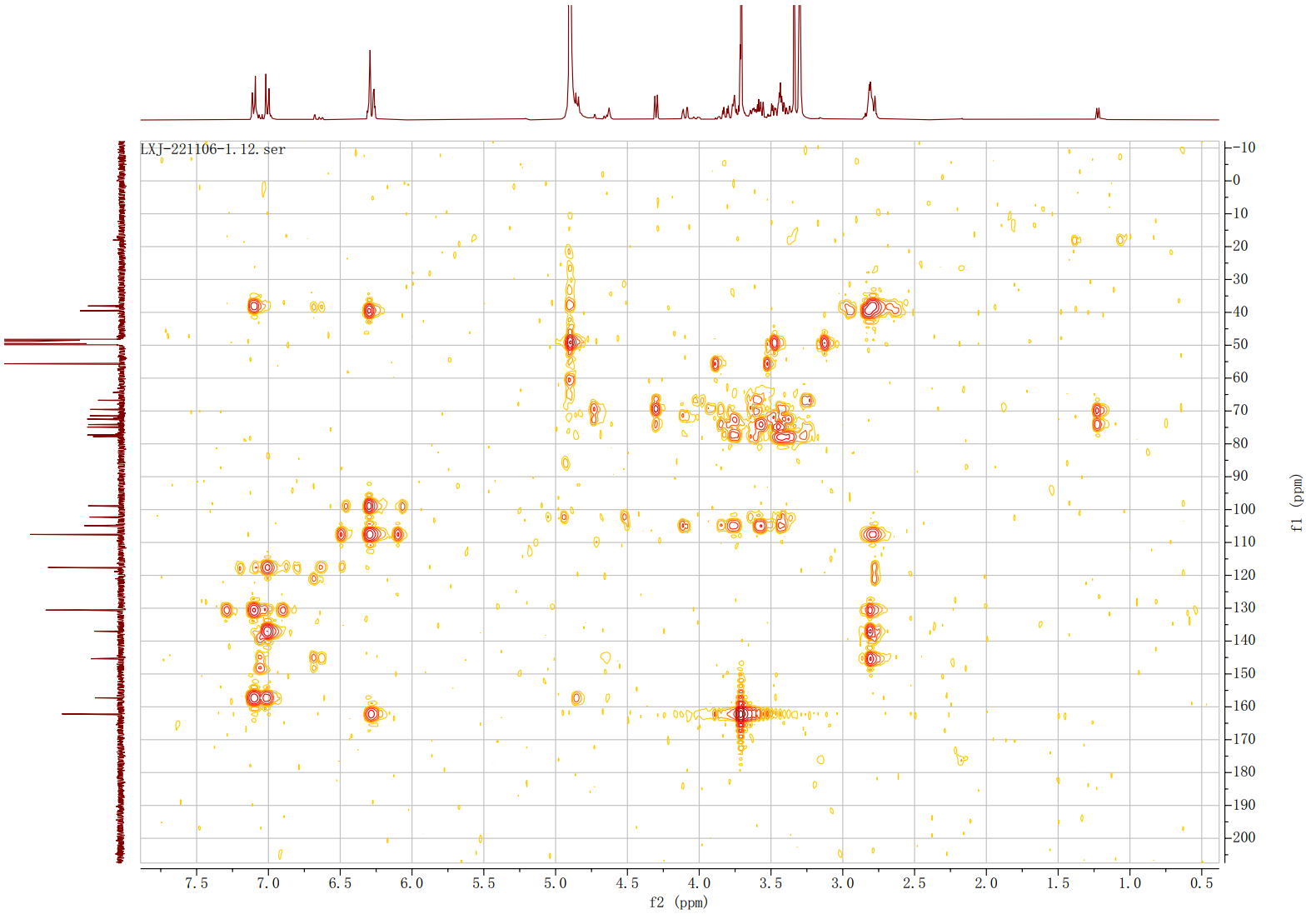


**Figure S23.** HMBC spectrum of **2** in methanol-*d_4_*


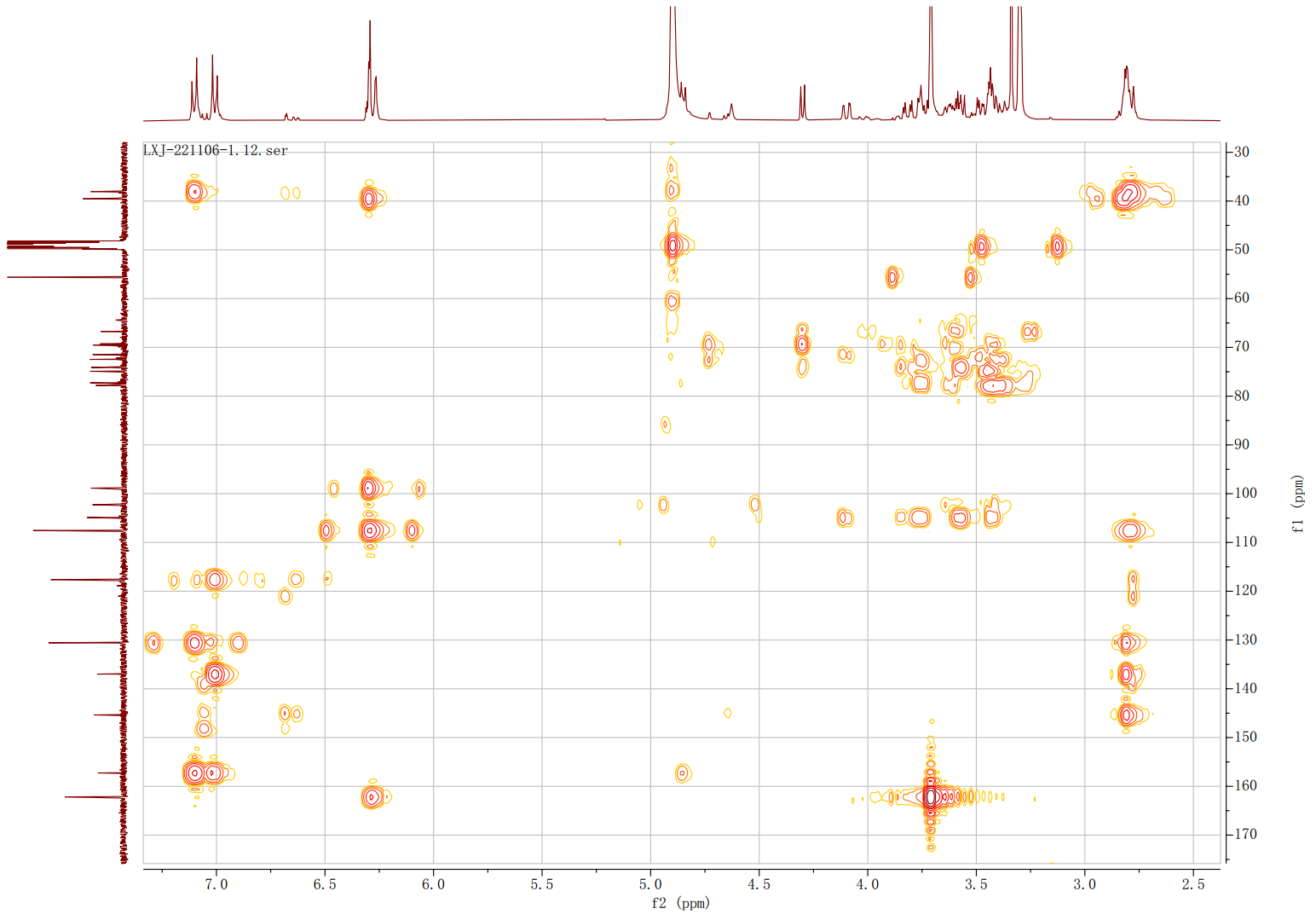


**Figure S24.** Expand HMBC spectrum of **2** in methanol-*d_4_*


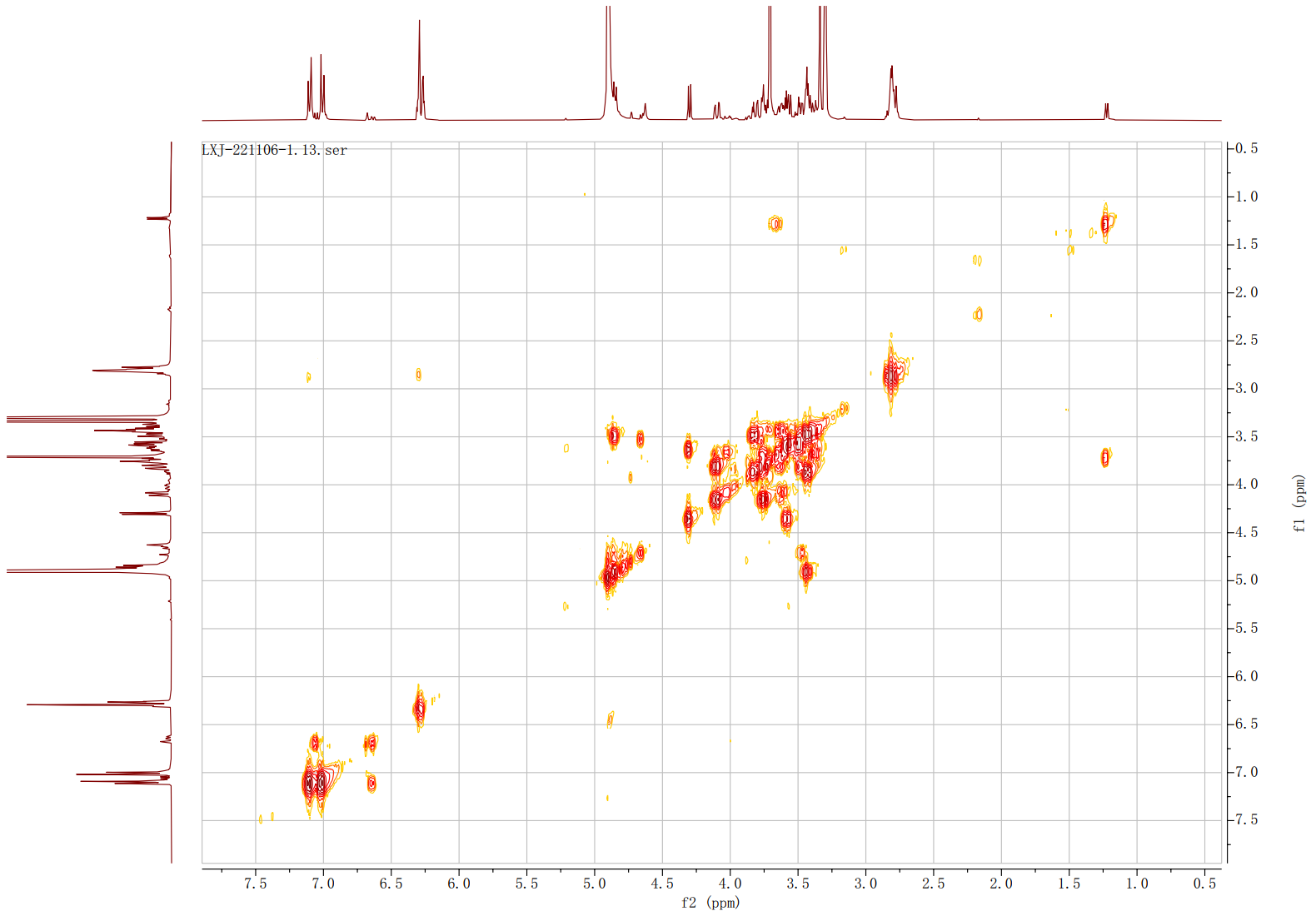


**Figure S25.** ^1^H-^1^H COSY spectrum of **2** in methanol-*d_4_*


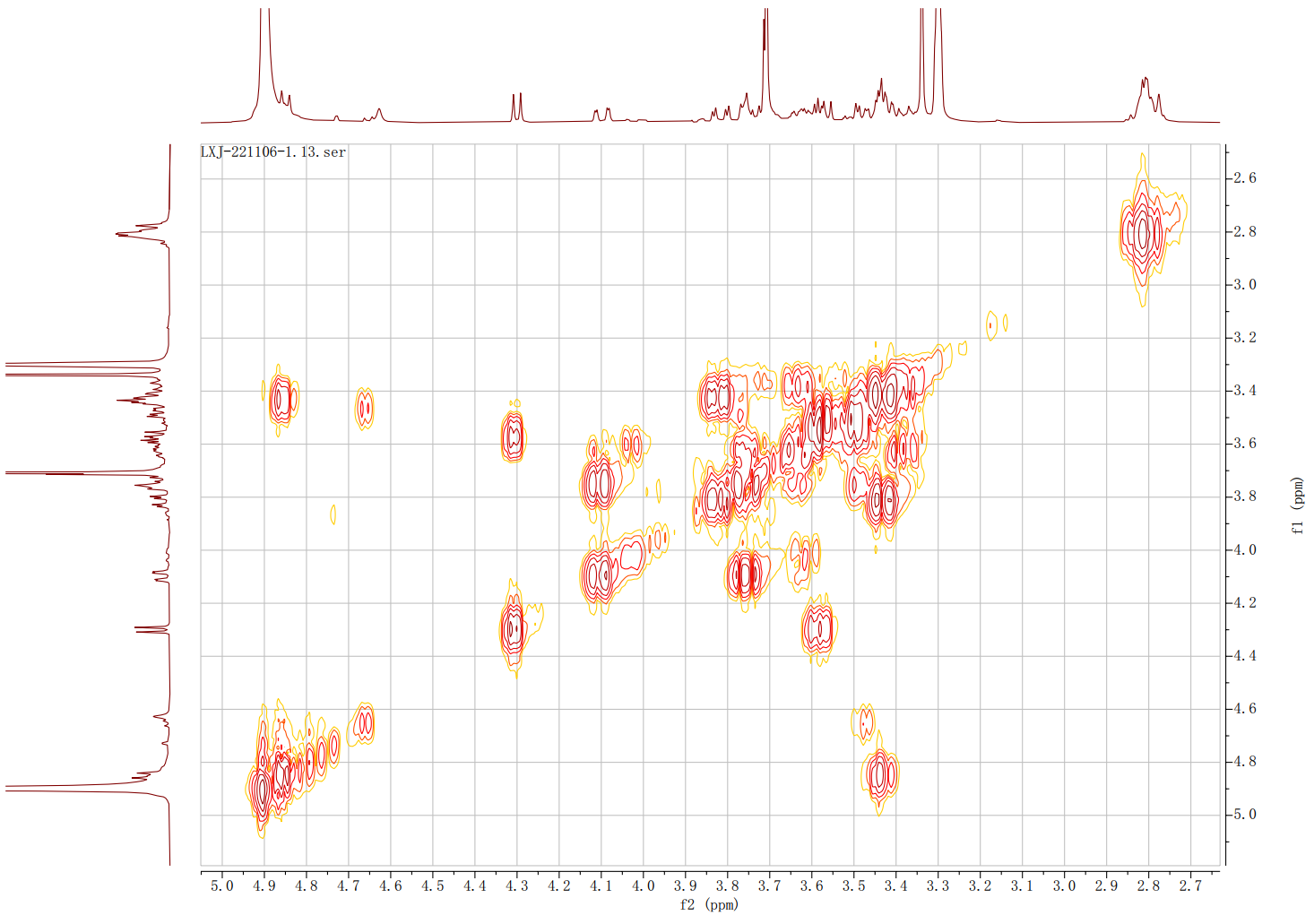


**Figure S26.** Expand ^1^H-^1^H COSY spectrum of **2** in methanol-*d_4_*


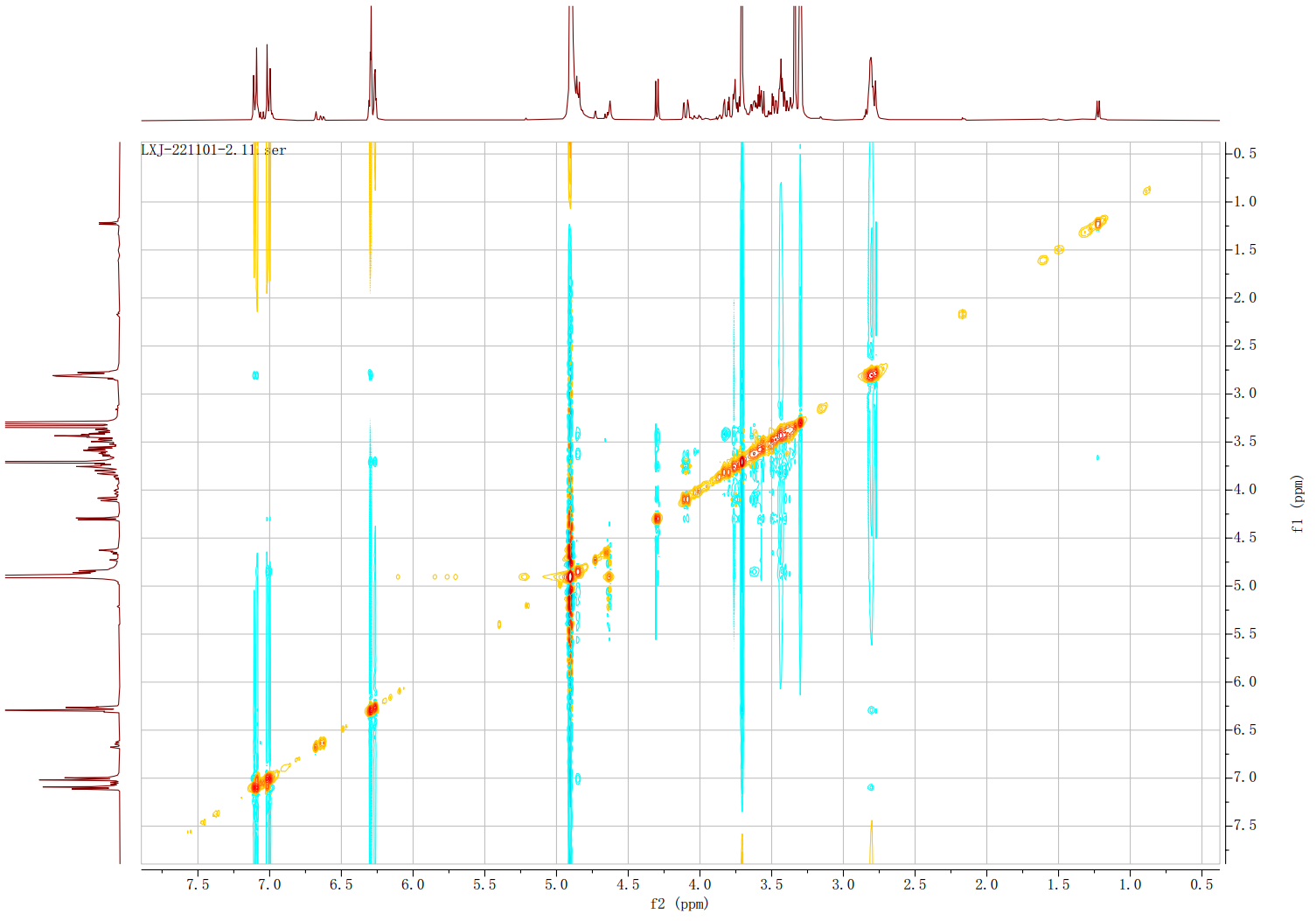


**Figure S27.** NOESY spectrum of **2** in methanol-*d_4_*


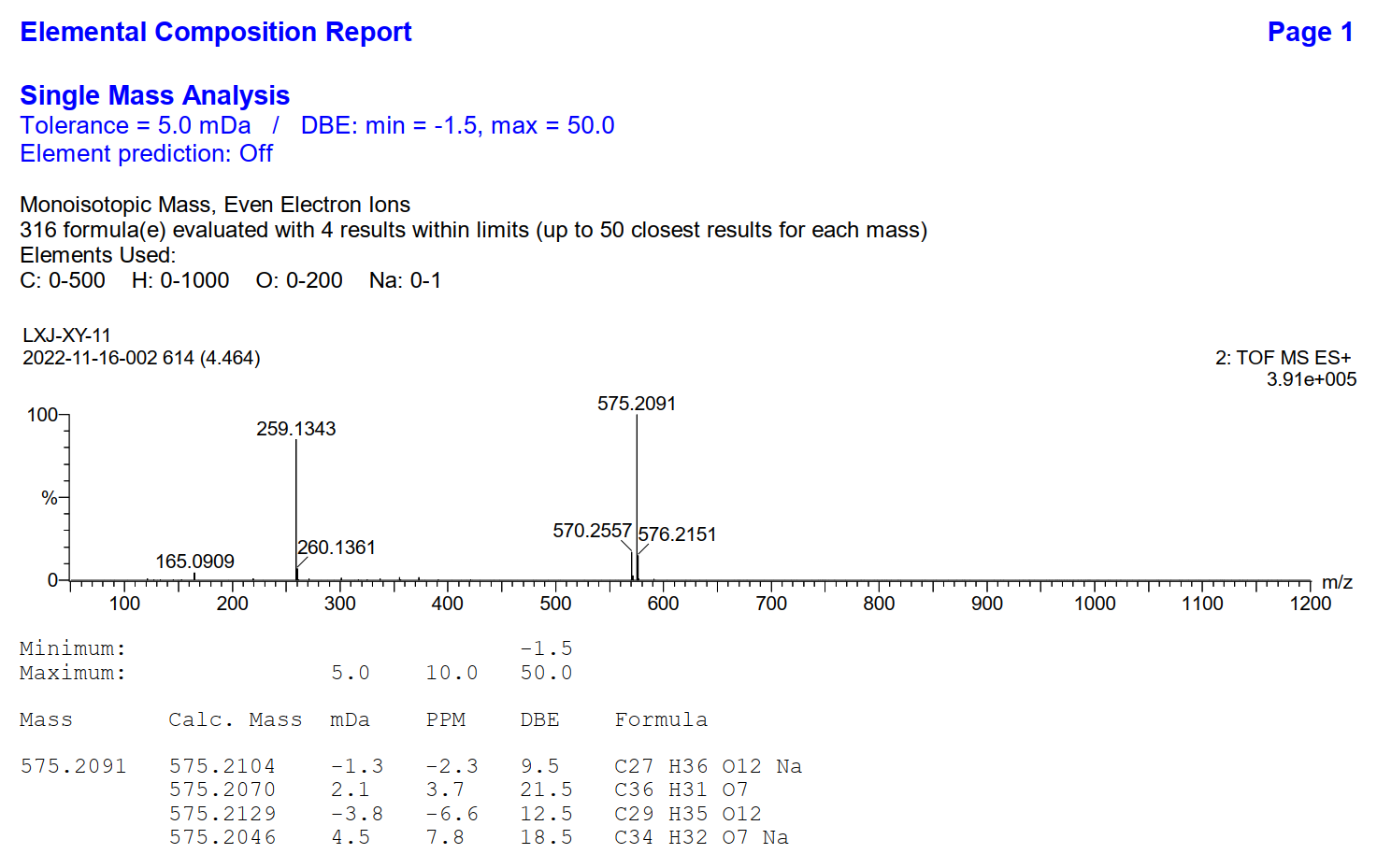


**Figure S28.** HRESIMS spectrum of **2**


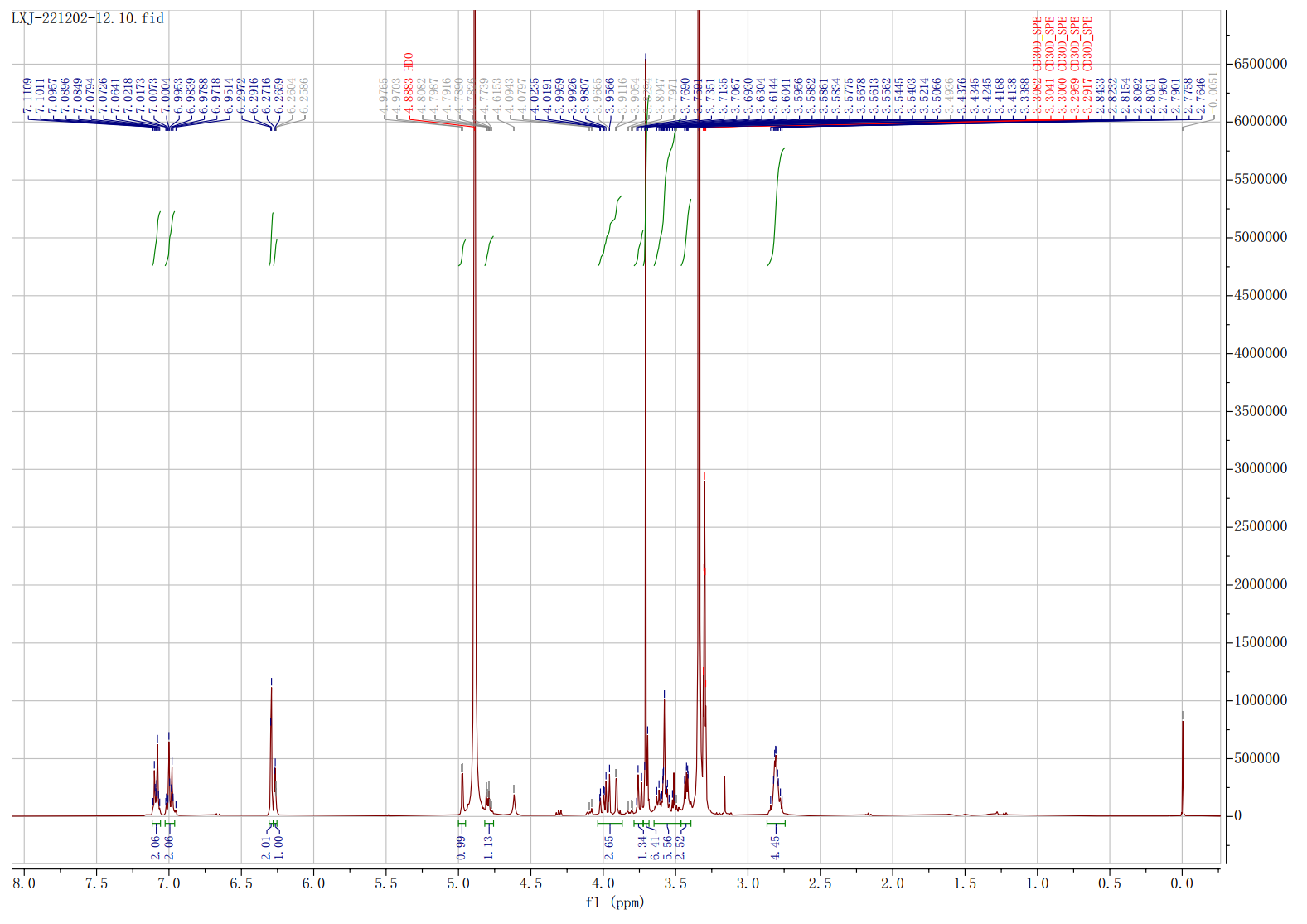


**Figure S31.** ^1^H NMR spectrum of **3** in methanol-*d_4_*


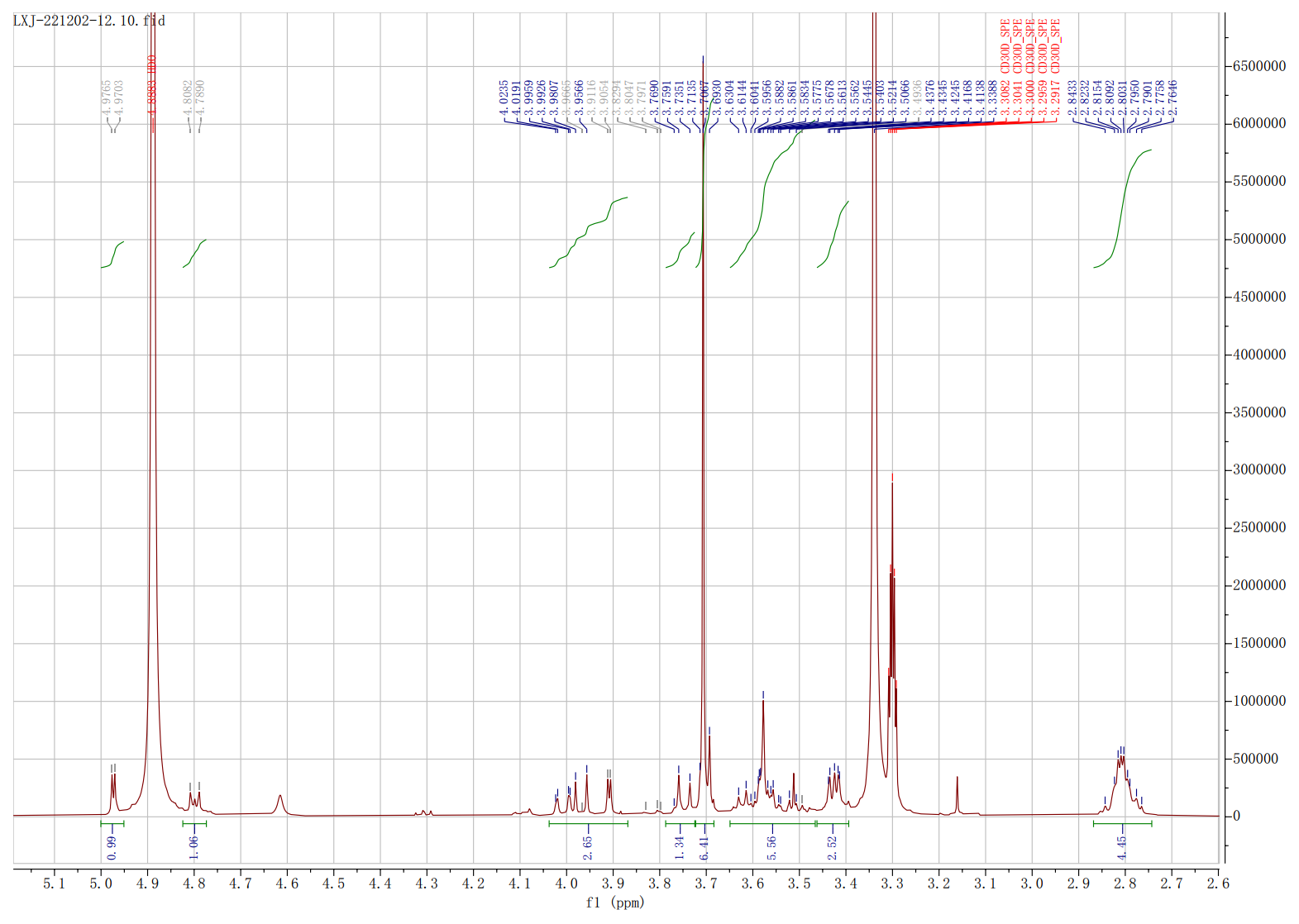


**Figure S32.** Expand ^1^H NMR spectrum of **3** in methanol-*d_4_*


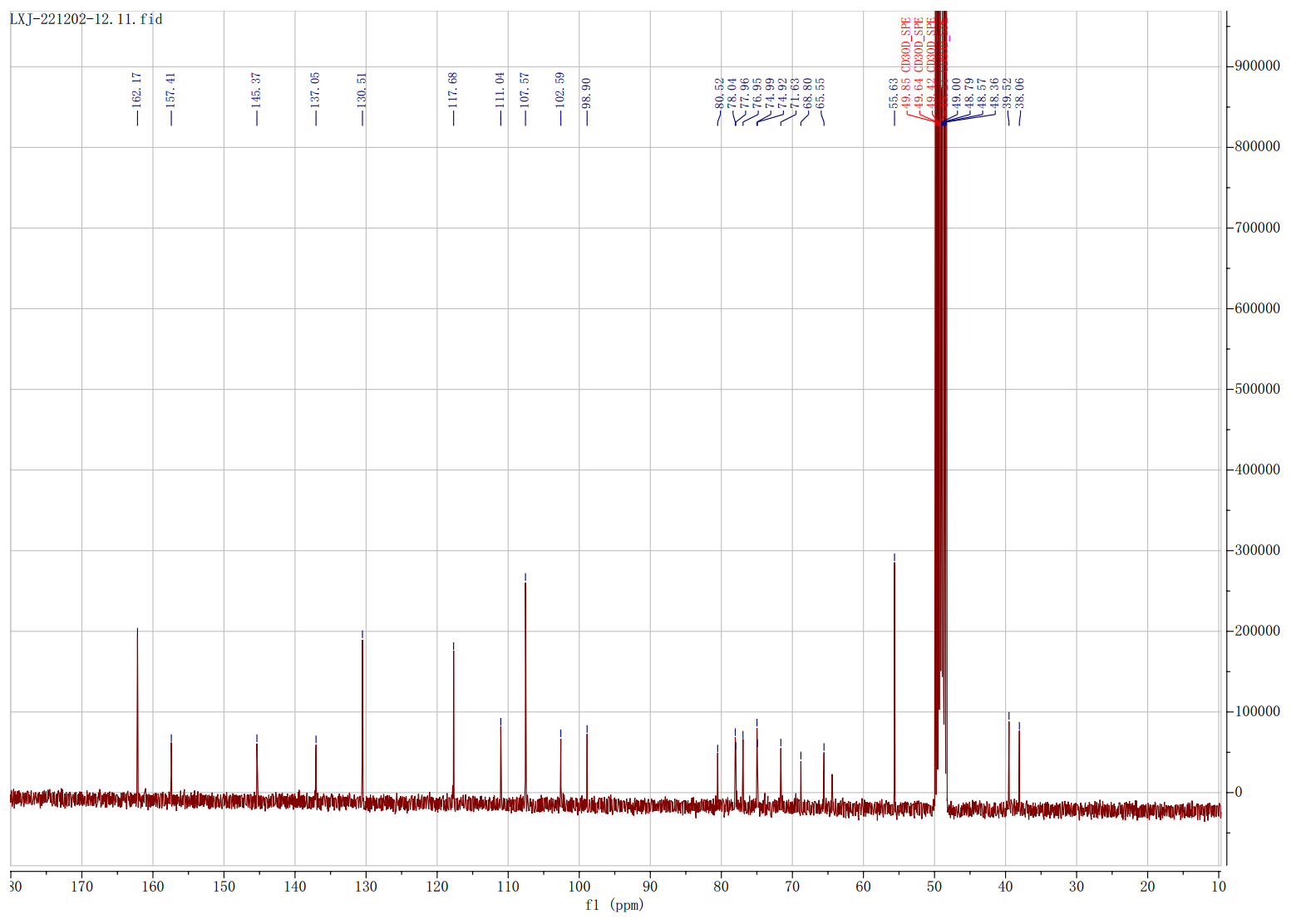


**Figure S33.** ^13^C NMR spectrum of **3** in methanol-*d_4_*


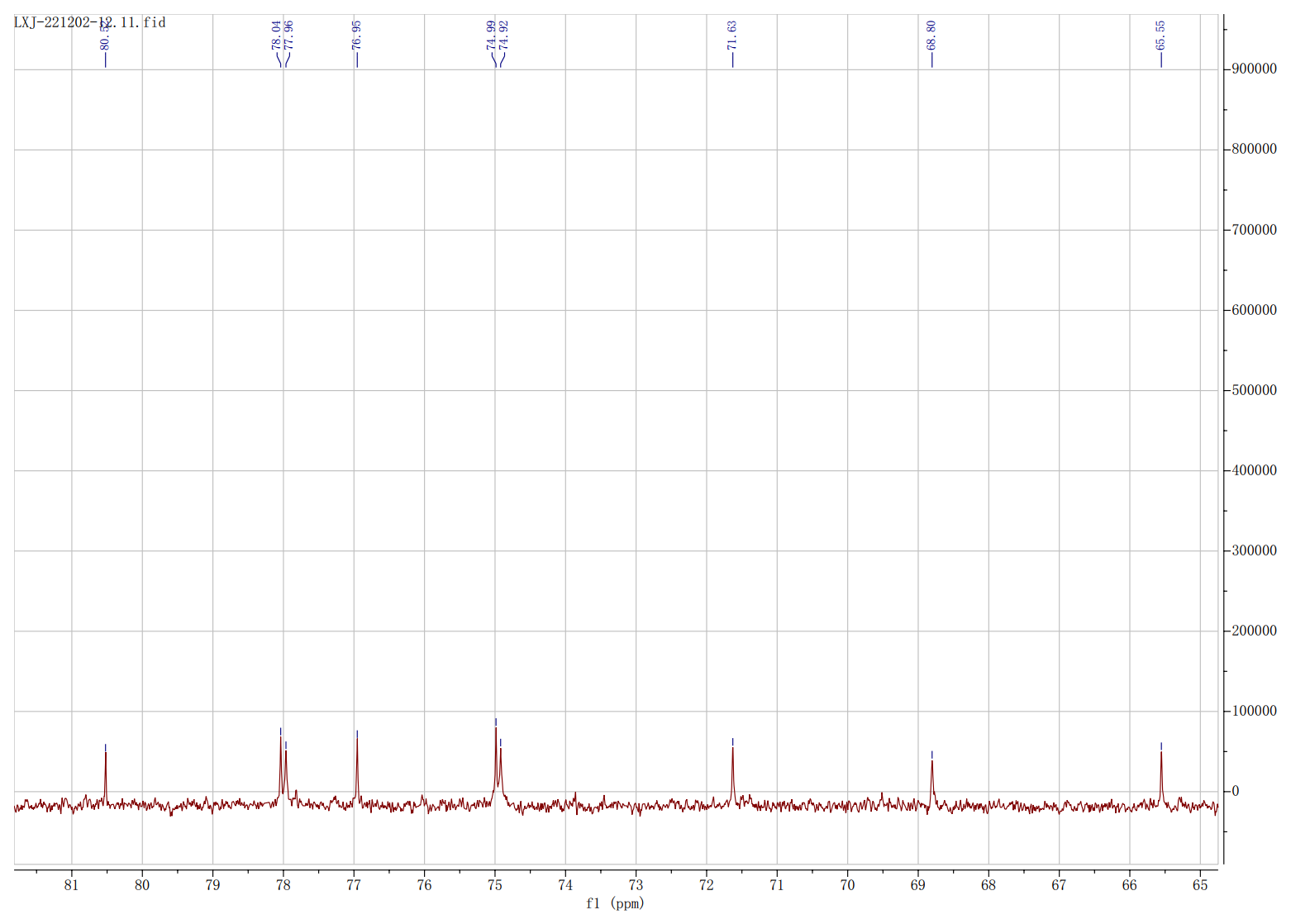


**Figure S34.** Expand ^13^C NMR spectrum of **3** in methanol-*d_4_*


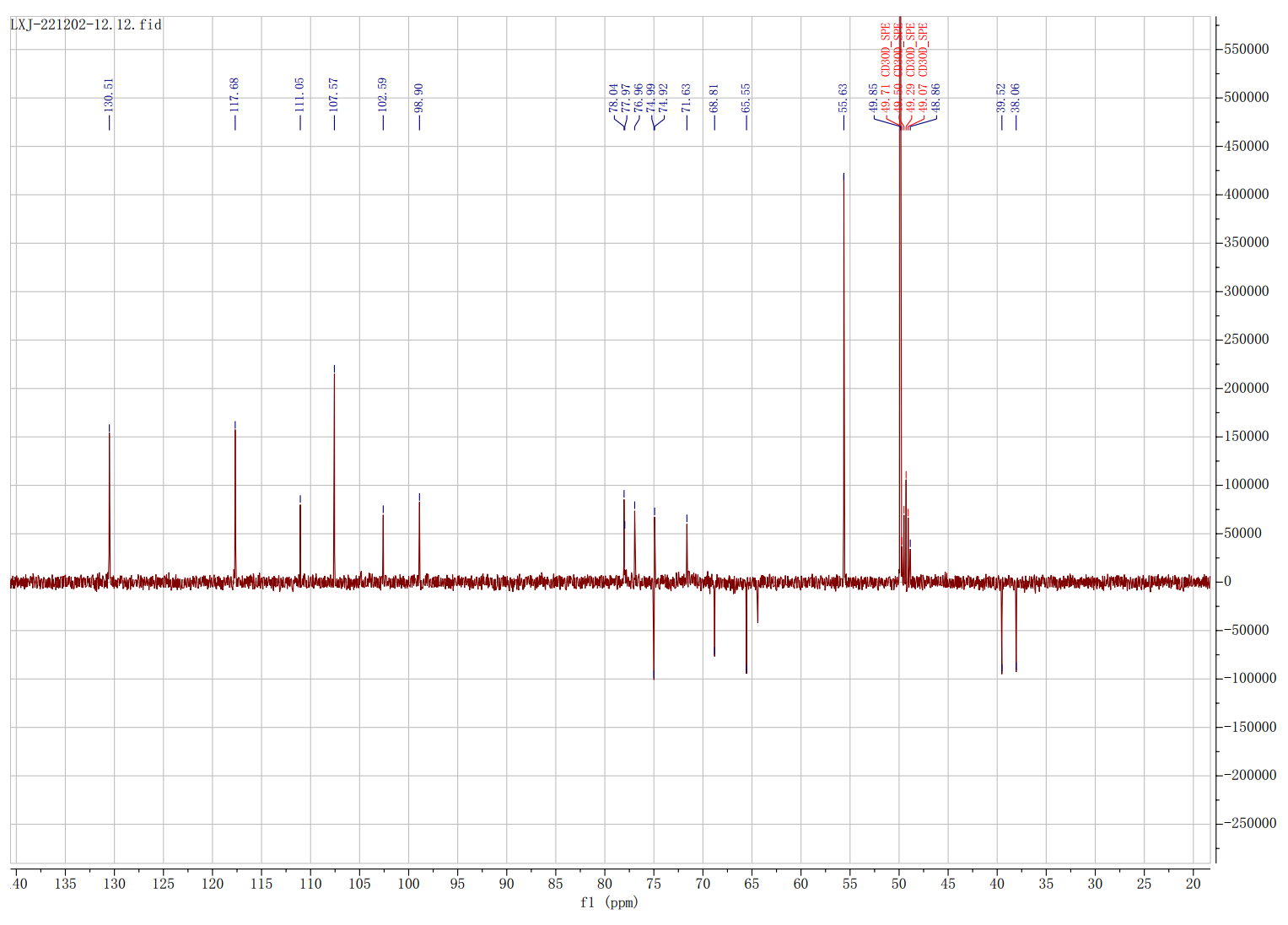


**Figure S35.** DEPT spectrum of **3** in methanol-*d_4_*


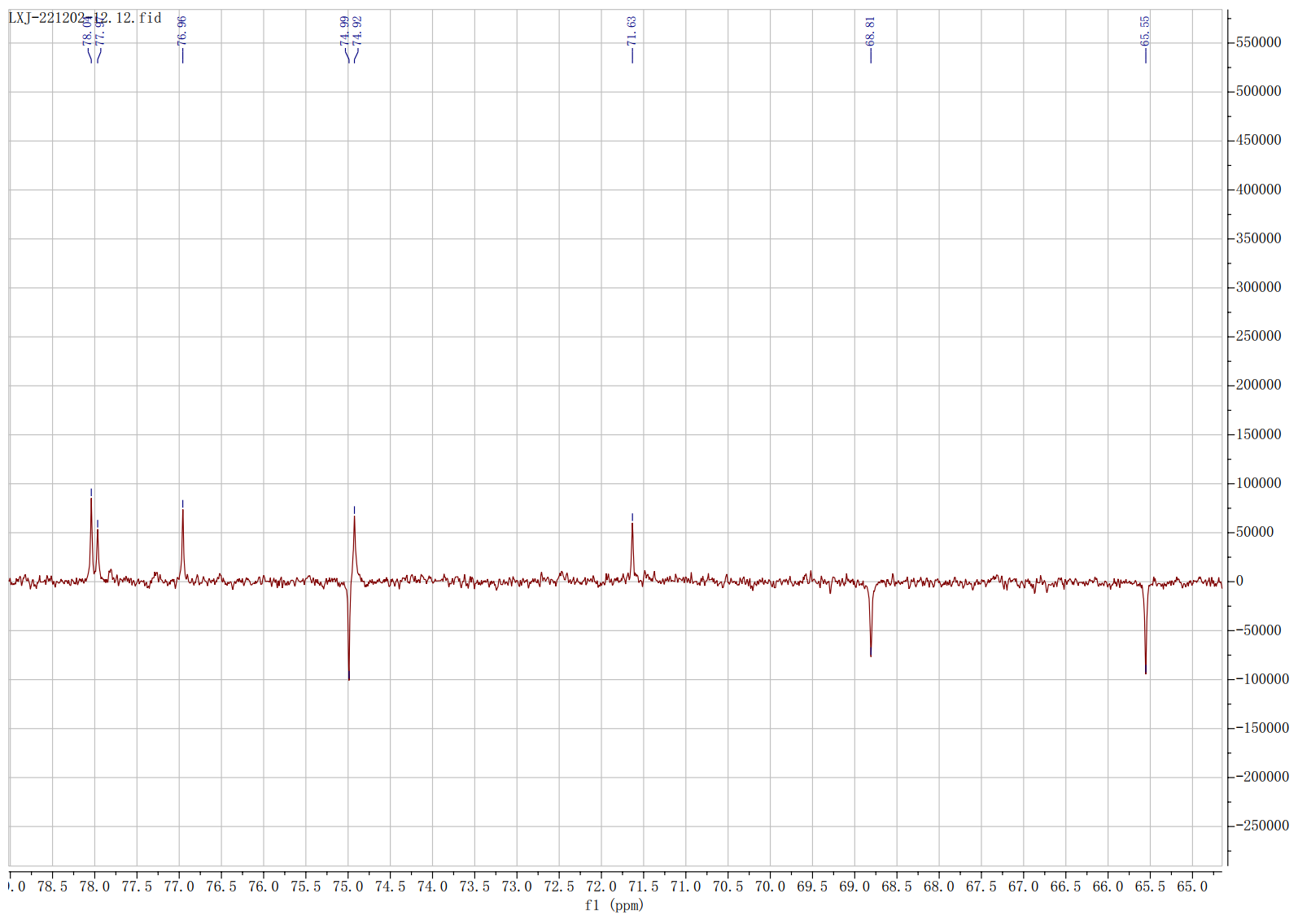


**Figure S36.** Expand DEPT spectrum of **3** in methanol-*d_4_*


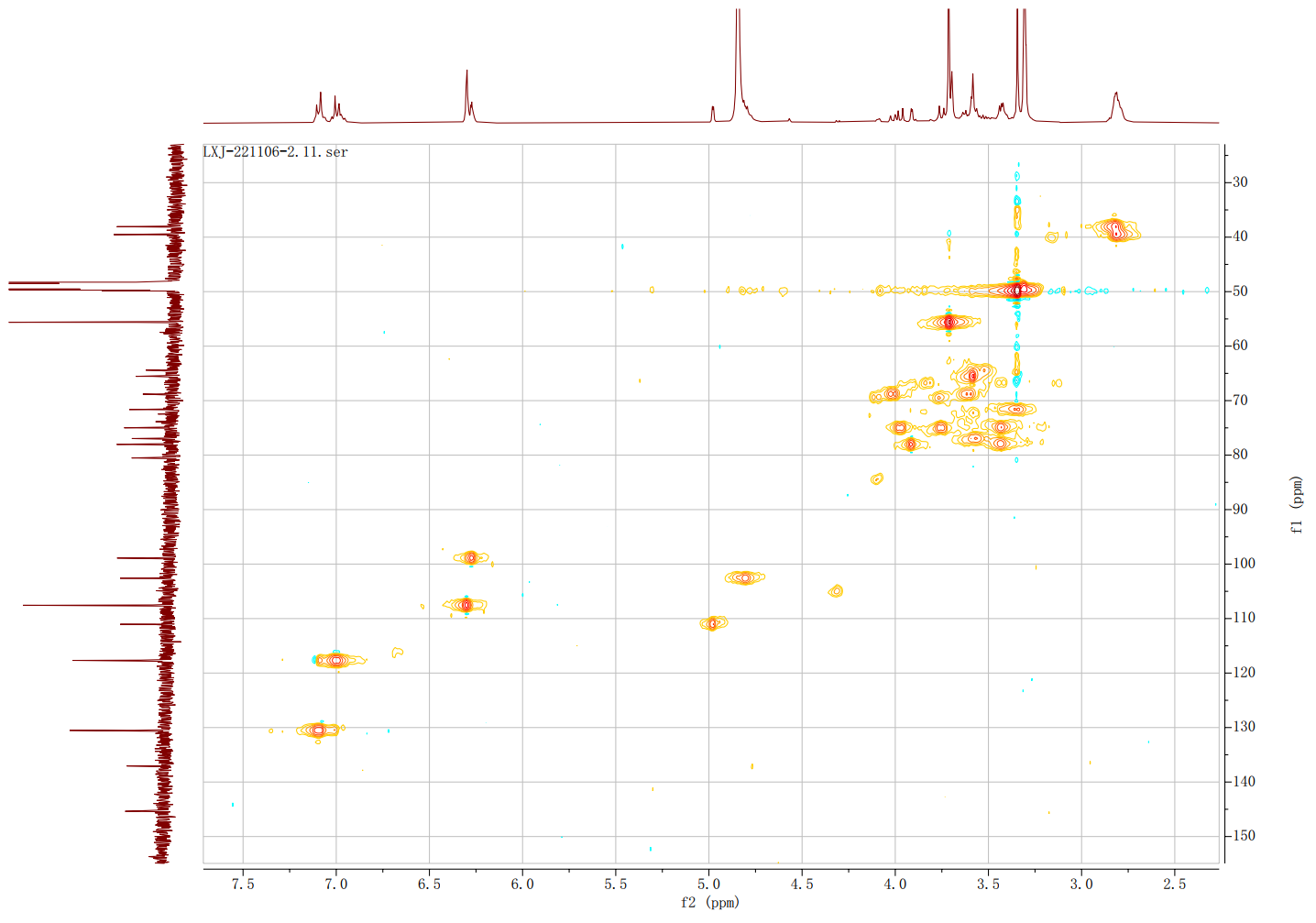


**Figure S37.** HSQC spectrum of **3** in methanol-*d_4_*


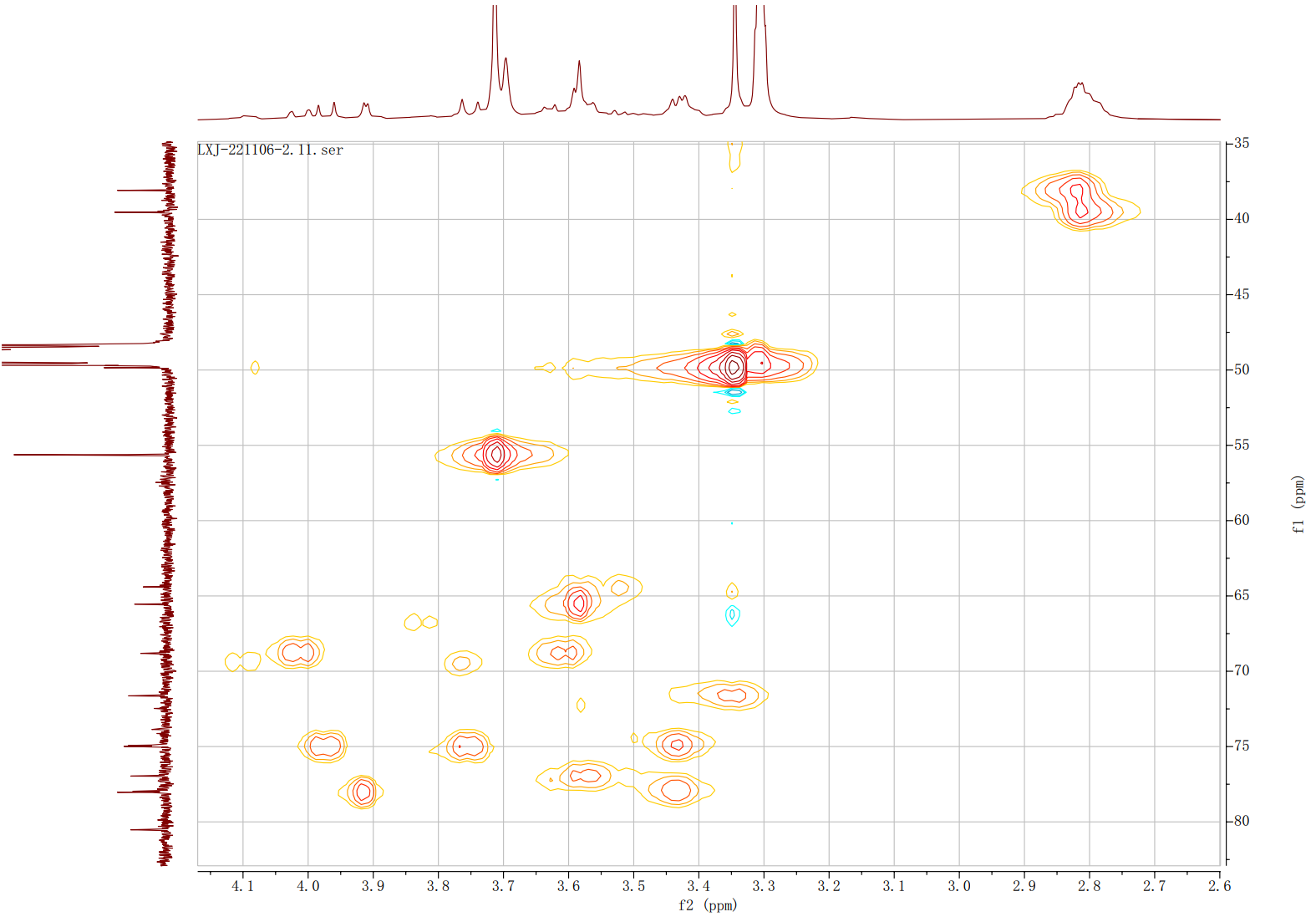


**Figure S38.** Expand HSQC spectrum of **3** in methanol-*d_4_*


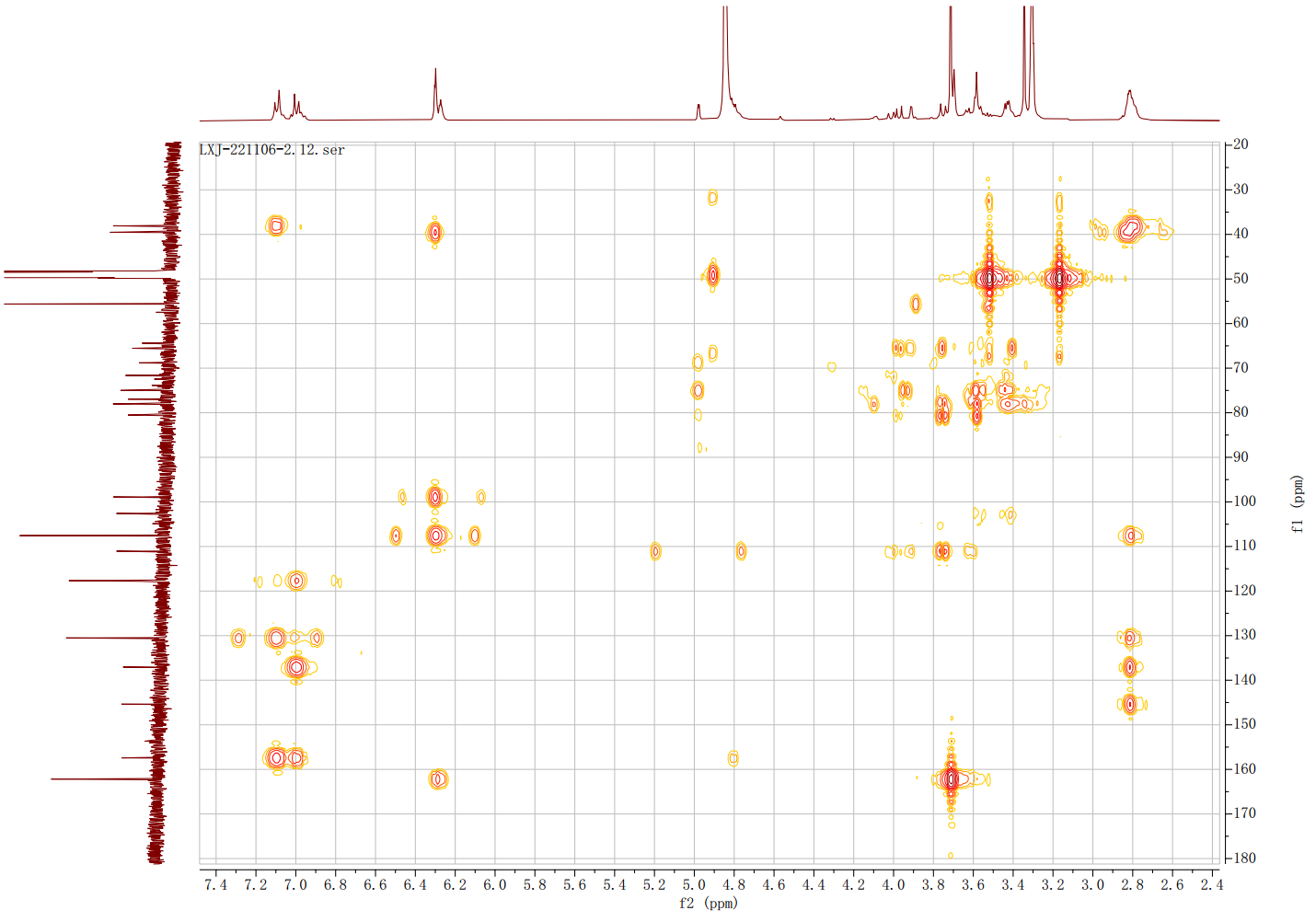


**Figure S39.** HMBC spectrum of **3** in methanol-*d_4_*


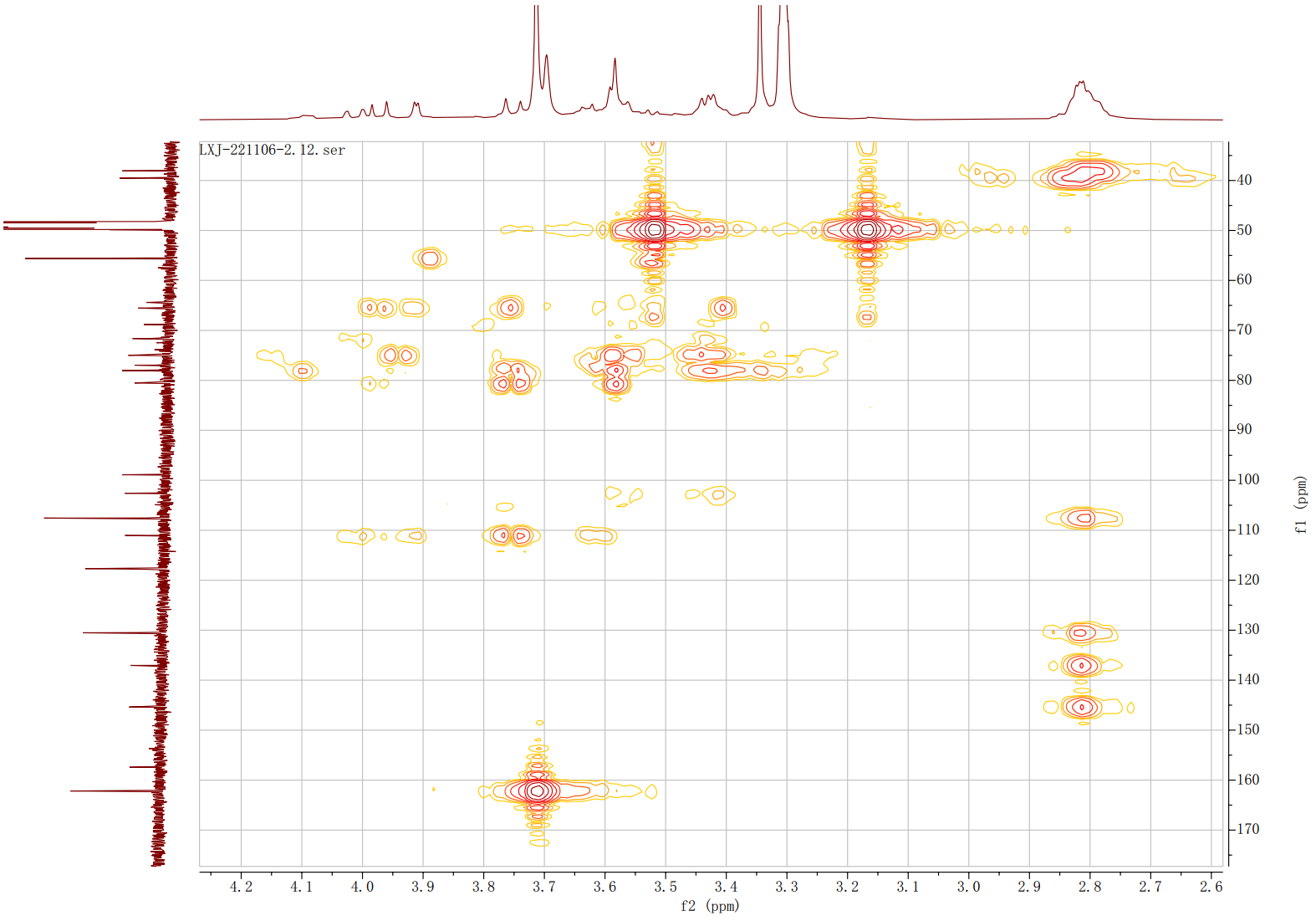


**Figure S40.** Expand HMBC spectrum of **3** in methanol-*d_4_*


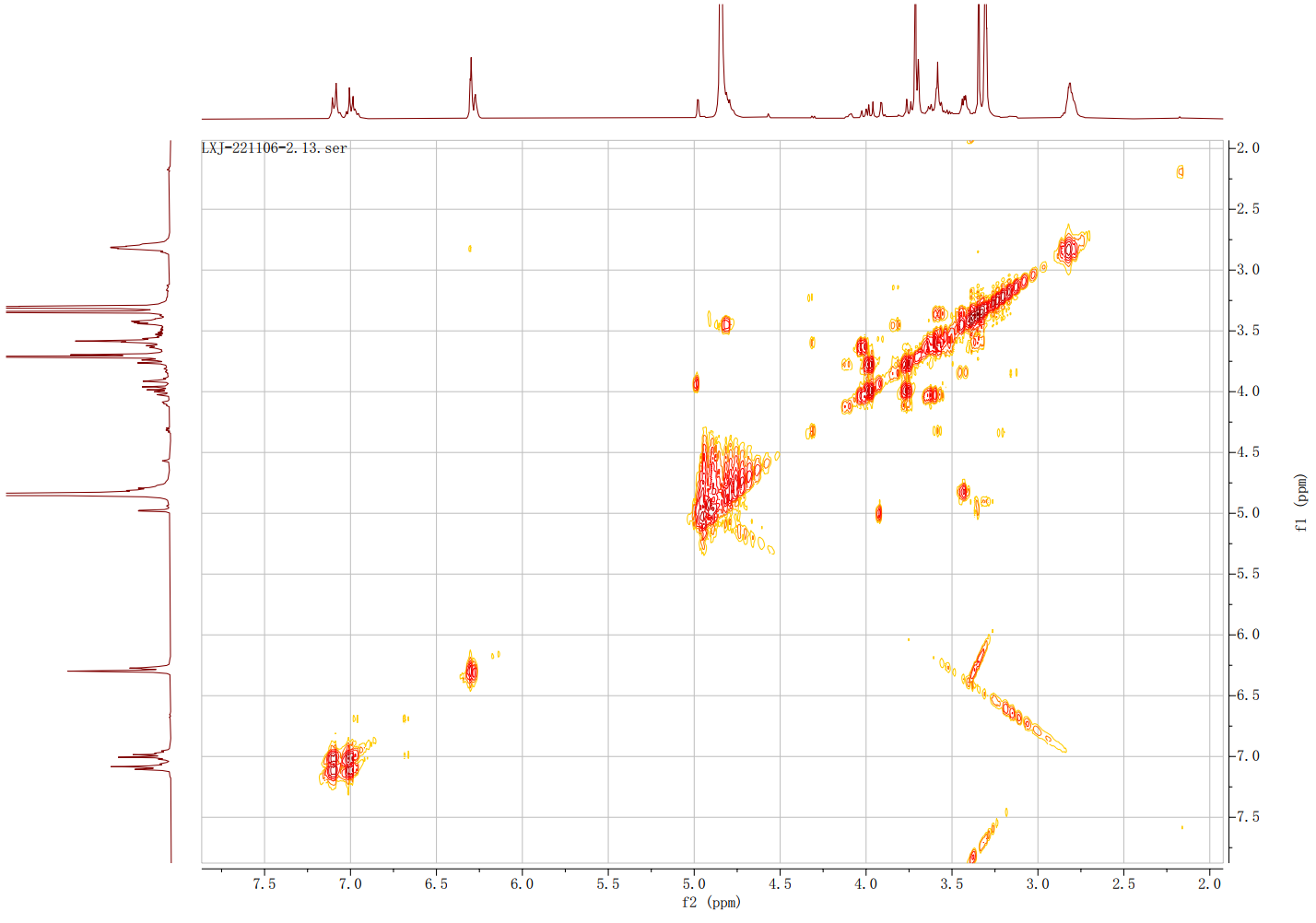


**Figure S41.** ^1^H-^1^H COSY spectrum of **3** in methanol-*d_4_*


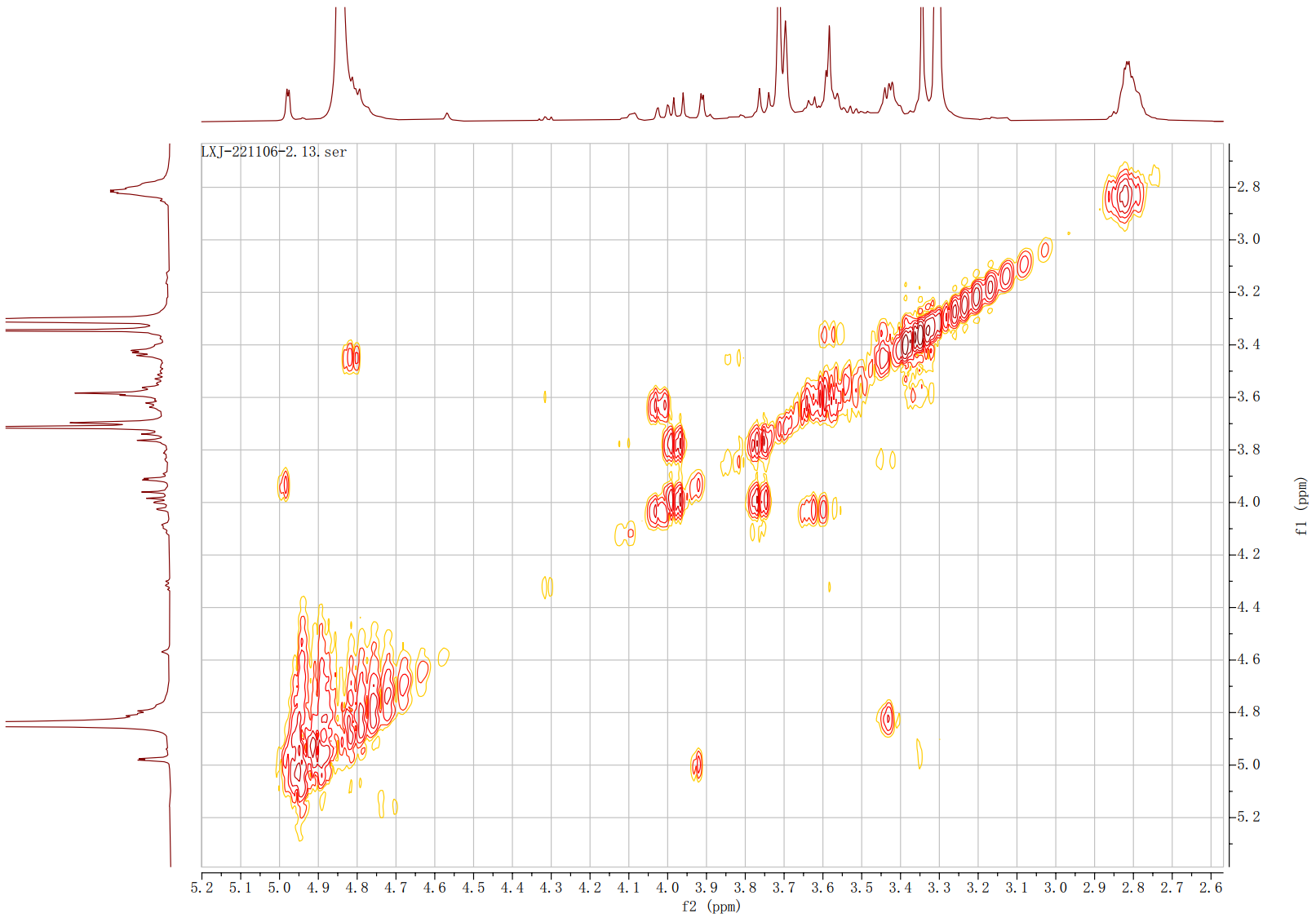


**Figure S42.** Expand ^1^H-^1^H COSY spectrum of **3** in methanol-*d_4_*


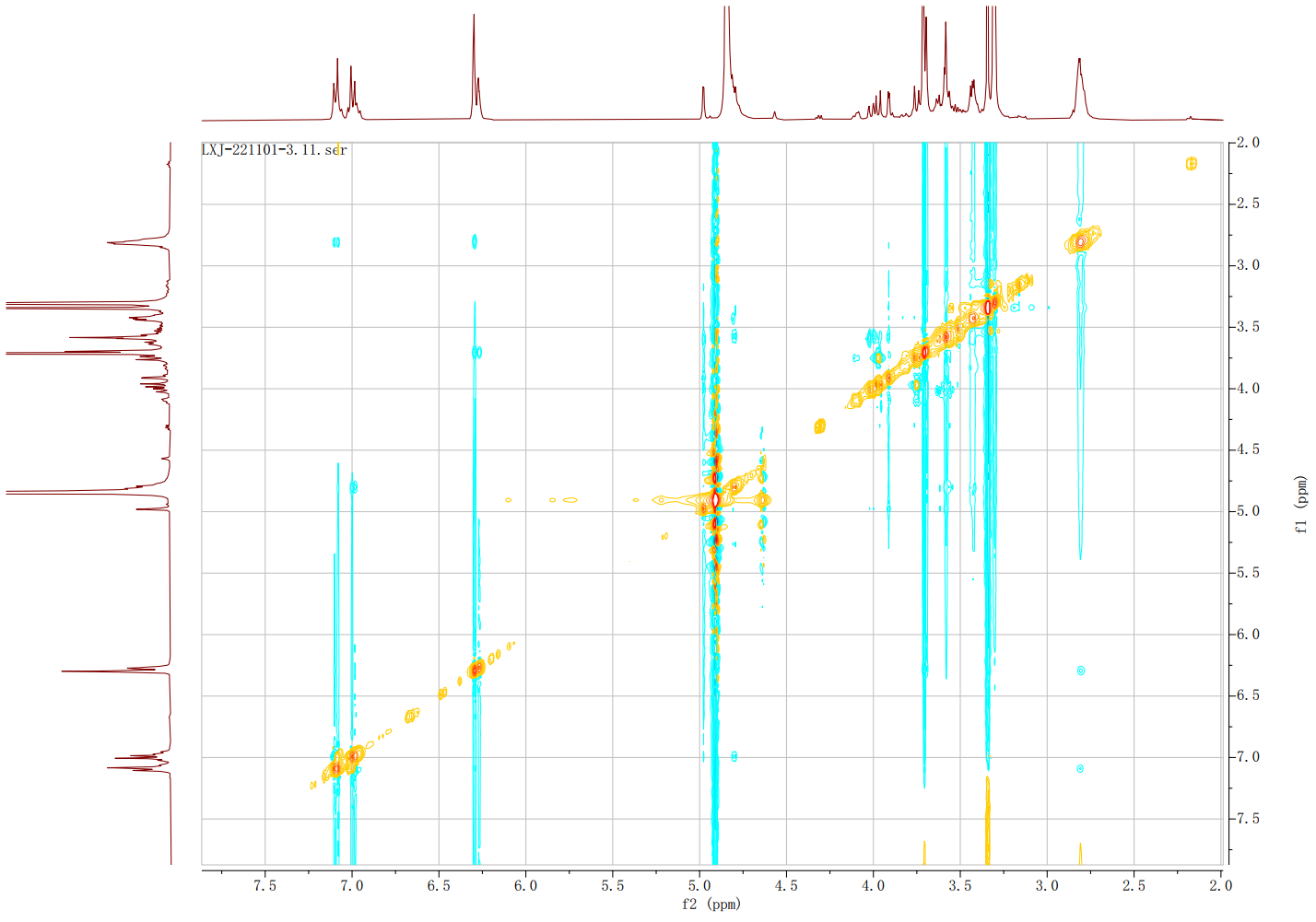


**Figure S43.** NOESY spectrum of **3** in methanol-*d_4_*


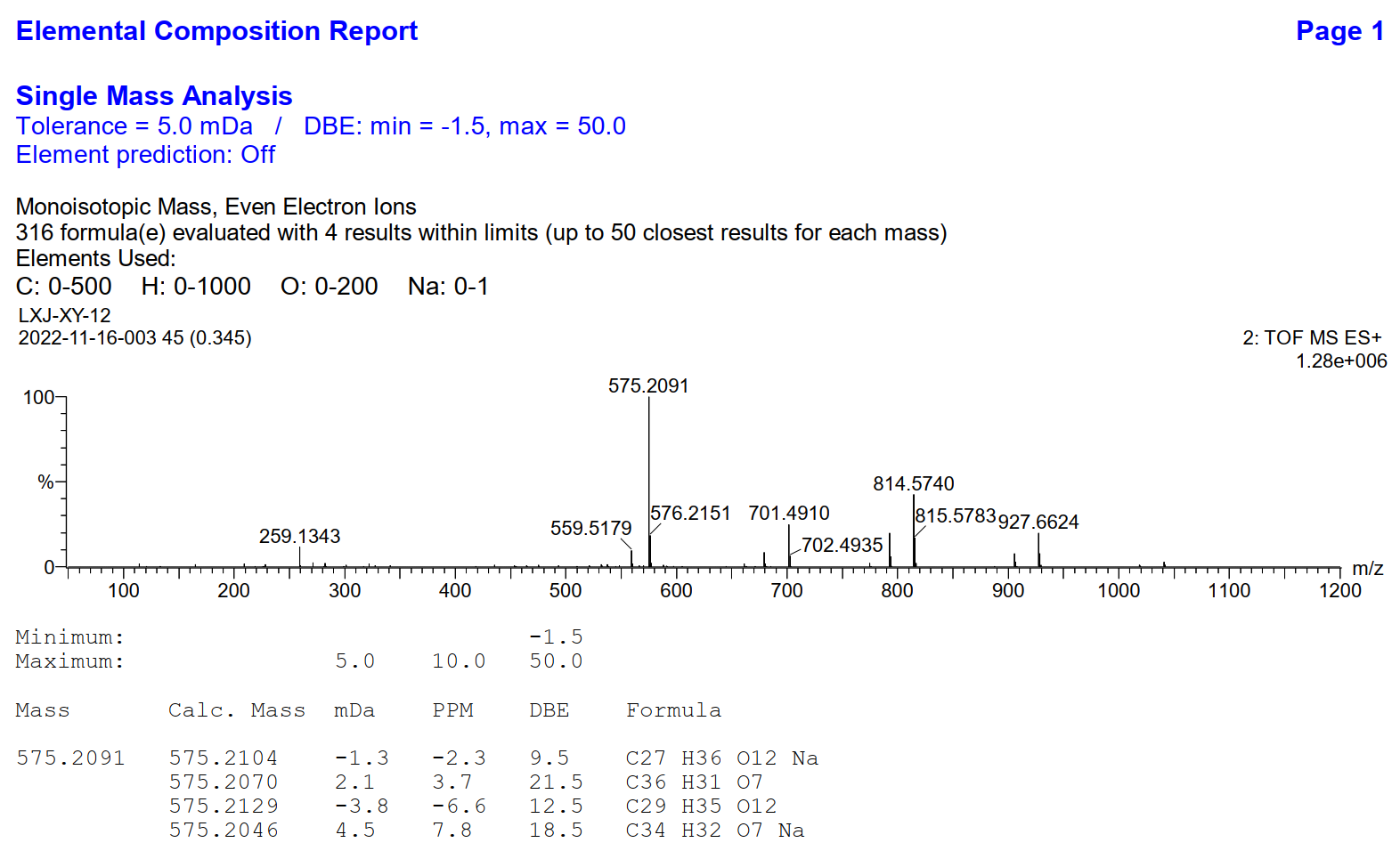


**Figure S44.** HRESIMS spectrum of **3**

**2.Acid hydrolysis and derivatization for the absolute configuration of sugar unit of undescribed compounds 1-3**

*2.1 Acid hydrolysis of* ***1-3***

Compounds **1-3** (each 1.5 mg) was dissolved in 500 μL of 2M HCl and heated at 90°C for 2 hrs, respectively. After hydrolysis, the reaction mixture was neutralized with 500 μL of 2M NH_4_OH, and dried with evaporator.

*2.2 Derivatization of the samples* ***1-3*** *(or sugar standards)*

Hydrolysed samples (**1-3**) or standard sugars (D-glucopyranose, L-arabinofuranose, L-arabinopyranose, and D-apiofuranose, each 5 mg) and L-cysteine methyl ester hydrochloride (5 mg) were dissolved in pyridine (1 mL) and heated 60°C for 1 h, and then 2-methylphenyl isothiocyanate (5 μL) was added to the mixture and heated further for 1h. The reaction mixture (20 μL) was analyzed by RP-HPLC and detected at 250 nm.

*2.3 HPLC analytical conditions*

Instrument: HPLC analysis system (Agilent 1200, CA, USA) with DAD detector

Column: YMC-Pack ODS-A column (250 mm × 4.6 mm, I.D., 5 µm)

Gradient of mobile phase: 15%-35% CH_3_CN-H_2_O in 0.1% HCOOH (0-50 min), 35%-90% CH_3_CN-H_2_O in 0.1% HCOOH (50-60 min), 90%-100% CH_3_CN-H_2_O in 0.1% HCOOH (60-70 min).

Detection wavelength: 250 nm

Injection: 20 µL

Flow rate: 0.8 mL/min

*2.4 Results*

The retention time (*t*_R_) values obtained were 35.087 min for D-glucopyranose (D-Glc), 37.319 min for L-arabinofuranose (L-Araf), 37.011 min for L-arabinopyranose (L-Arap), and 44.219 min for D-apiofuranose (D-Apif). The acid hydrolysis and derivatization of sugars, as well as the RP-HPLC analysis results, revealed the absolute configuration of monosaccharides in the new compounds **1-3**. The monosaccharides in **1-3** were identified as D-Glc and L-Araf, D-Glc and L-Arap, D-Glc and D-Apif, respectively, by comparison with authentic standards.

**Table S1**. The types of sugars and retention time of new compounds **1-3**.

| **New compounds** | **Types of sugars** | **Retention time (*t*_R_, min)** |
| --- | --- | --- |
| **1** | *β*-D-glucopyranose (D-Glc) | 35.087 |
|  | *α*-L-arabinofuranose (L-Araf) | 37.319 |
| **2** | *β*-D-glucopyranose (D-Glc) | 35.087 |
|  | *α*-L-arabinopyranose (L-Arap) | 37.011 |
| **3** | *β*-D-glucopyranose (D-Glc) | 35.087 |
|  | *β*-D-apiofuranose (D-Apif) | 44.219 |

**Reference**

1. T. Tanaka, T. Nakashima, T. Ueda, K. Tomii, I. Kouno, Facile discrimination of aldose enantiomers by reversed-phase HPLC, Chem. Pharm. Bull. 55 (2007) 899-901. doi: 10.1248/cpb.55.899. PMID: 17541189.
2. Y.H. Wang, B. Avula, X. Fu, M. Wang, I.A. Khan, Simultaneous determination of the absolute configuration of twelve monosaccharide enantiomers from natural products in a single injection by a UPLC-UV/MS method, Planta Med. 78 (2012) 834-837. doi: 10.1055/s-0031-1298432. Epub 2012 Apr 24. PMID: 22532020.

*2.5 HPLC chromatograms of samples and standard sugars after acid hydrolysis and derivatization*

**Compound** **1:**


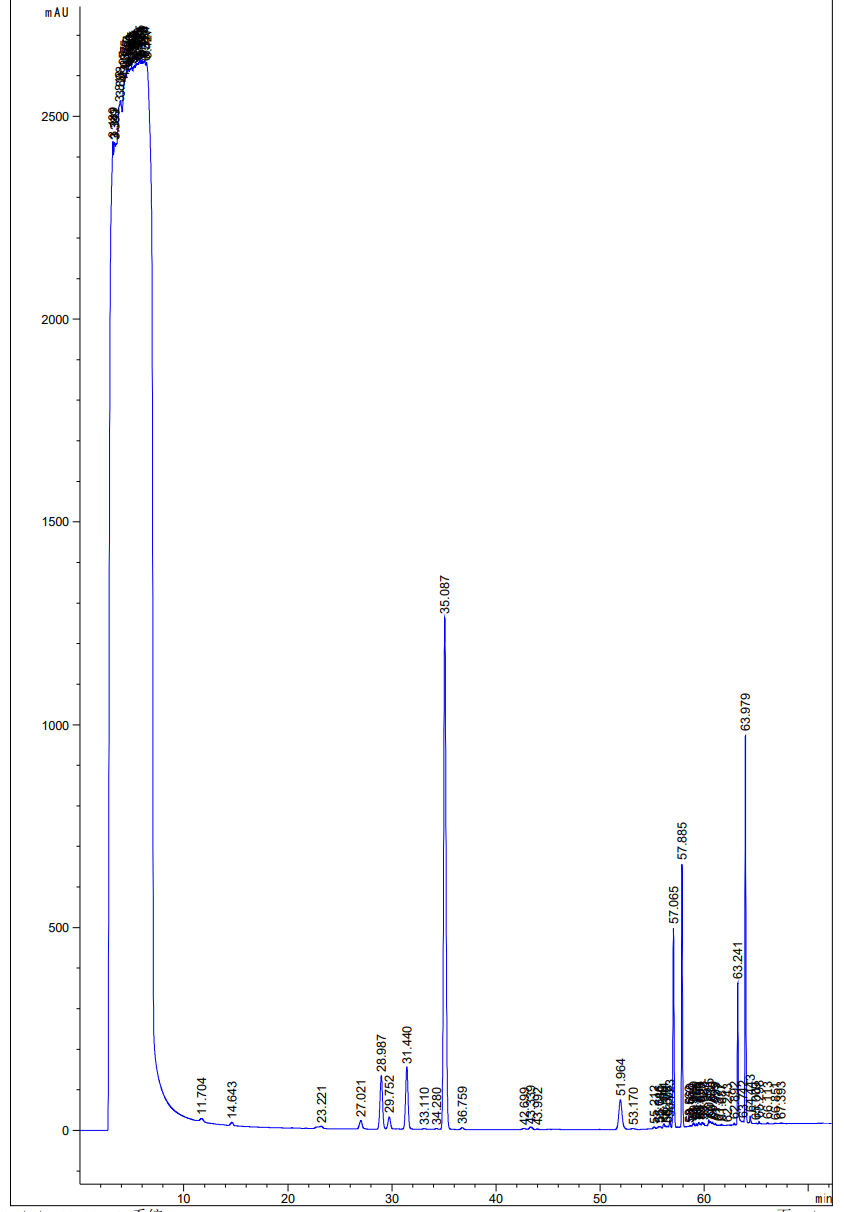

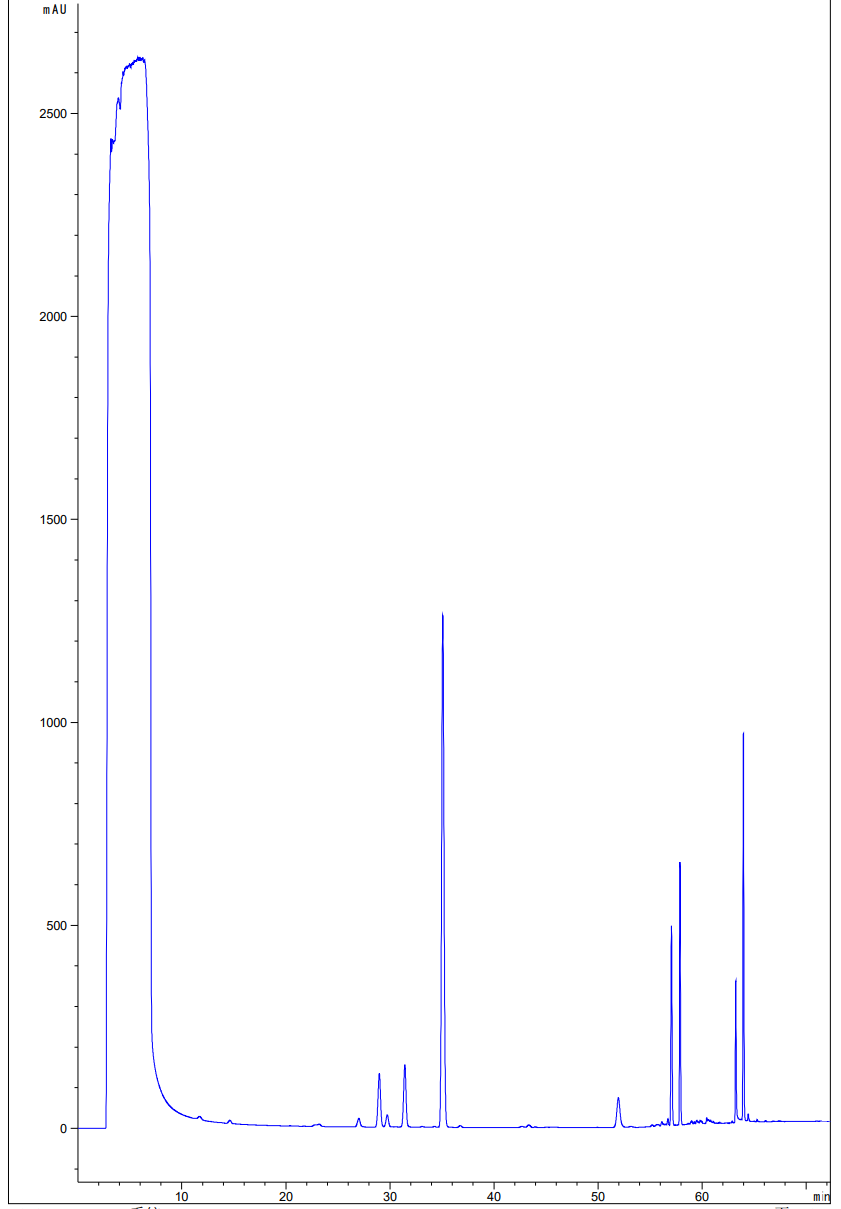


D-Glc

D-Glc

**Figure S45.** HPLC chromatogram of *D*-Glc standard derivative (Left: with *t*_R_; Right: without *t*_R_)


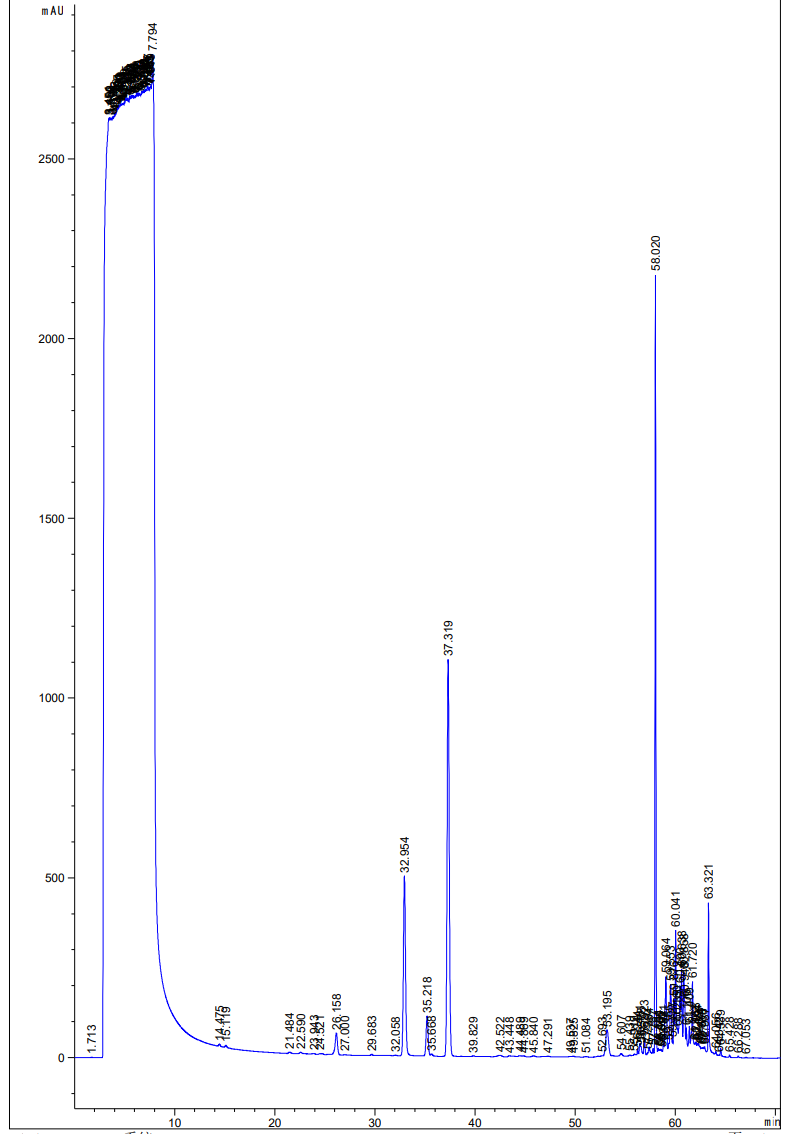

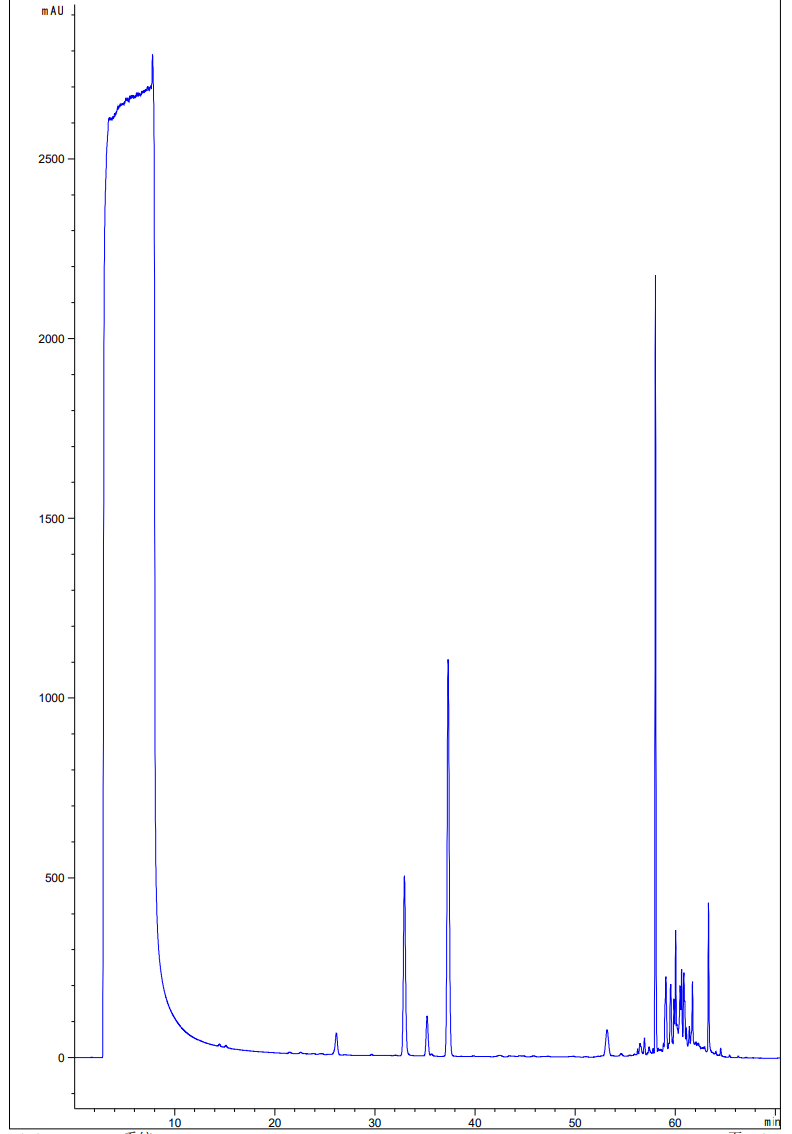


L-Araf

L-Araf

**Figure S46.** HPLC chromatogram of *L*-Araf standard derivative (Left: with *t*_R_; Right: without *t*_R_)


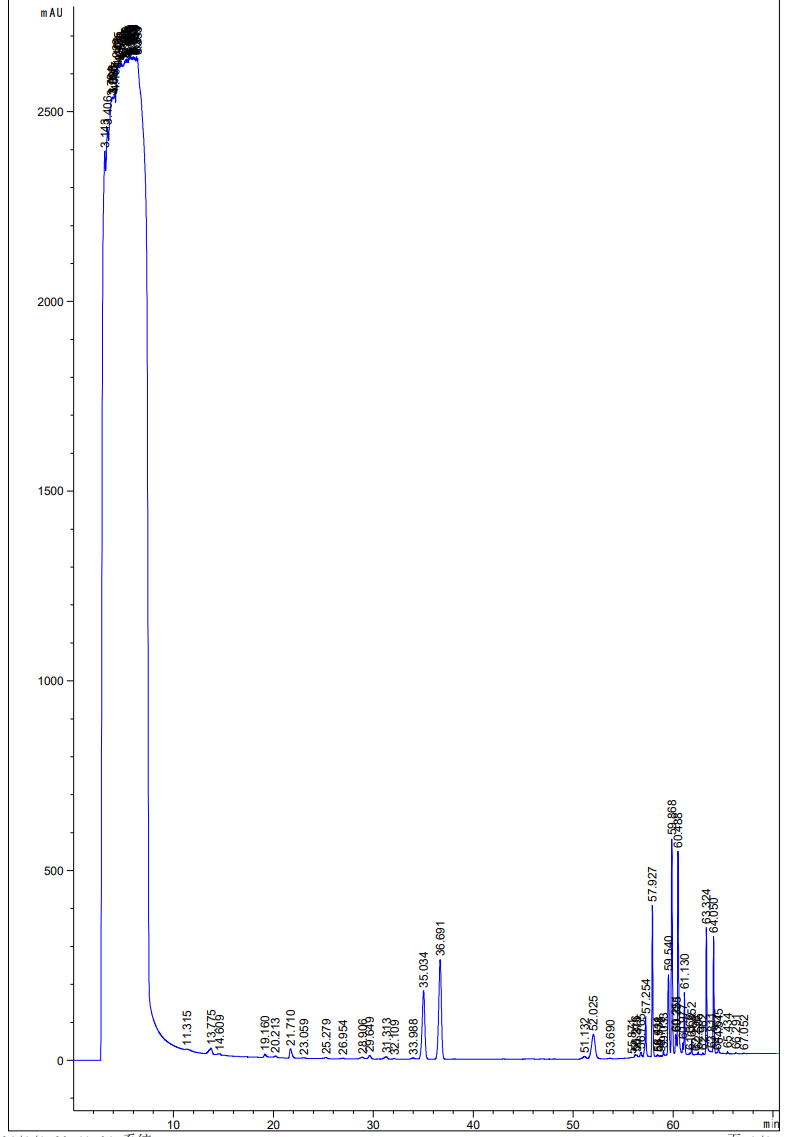

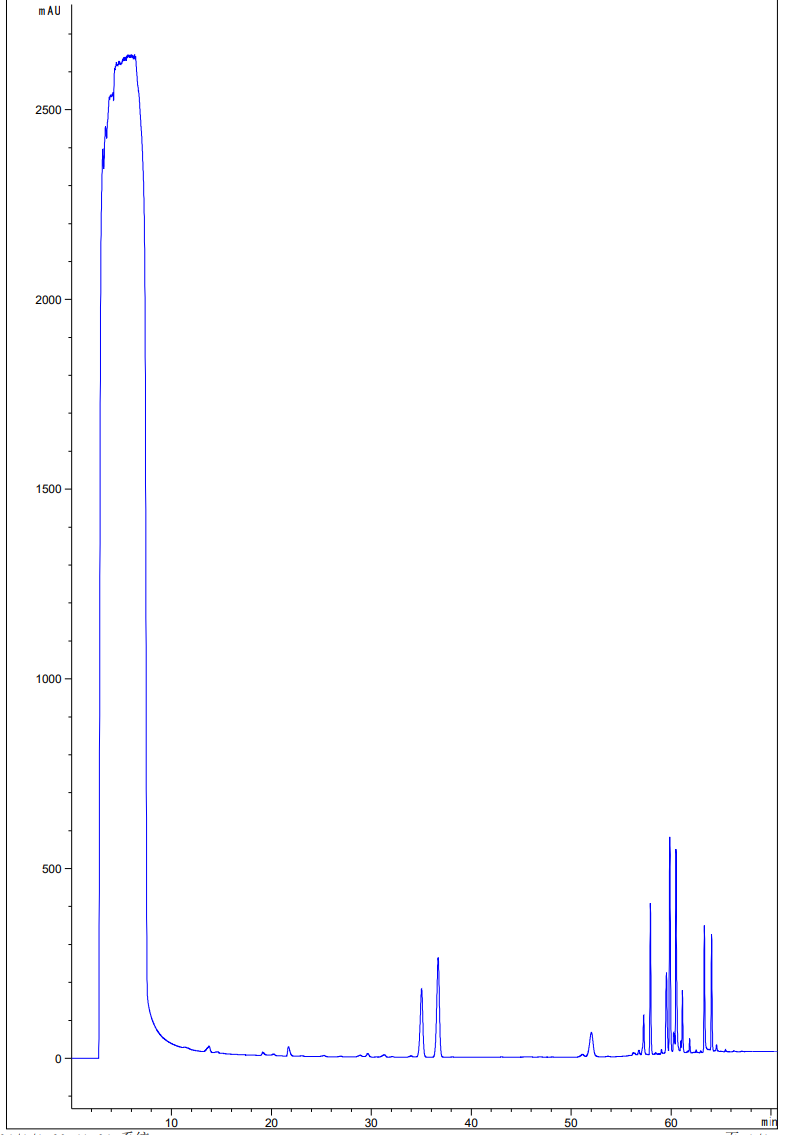


L-Araf

D-Glc

L-Araf

D-Glc

**Figure S47.** HPLC chromatogram of compound **1** after acid hydrolysis and sugar derivatization

(Left: with *t*_R_; Right: without *t*_R_)


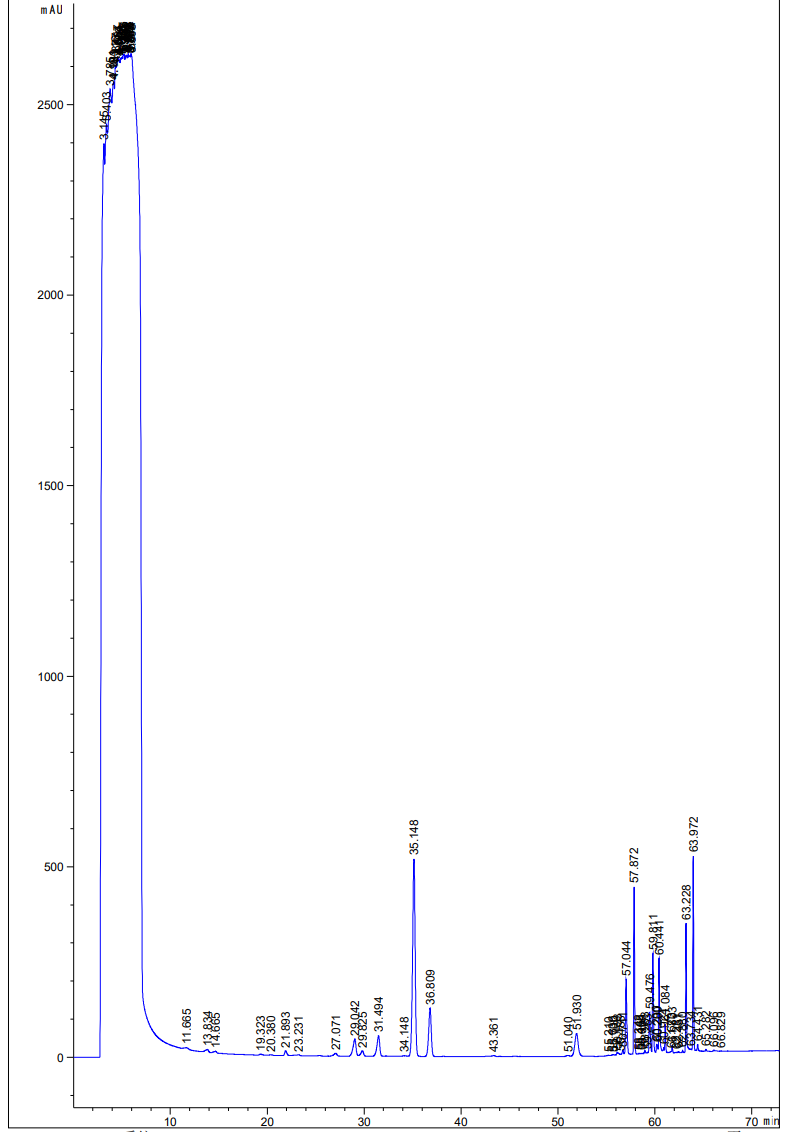

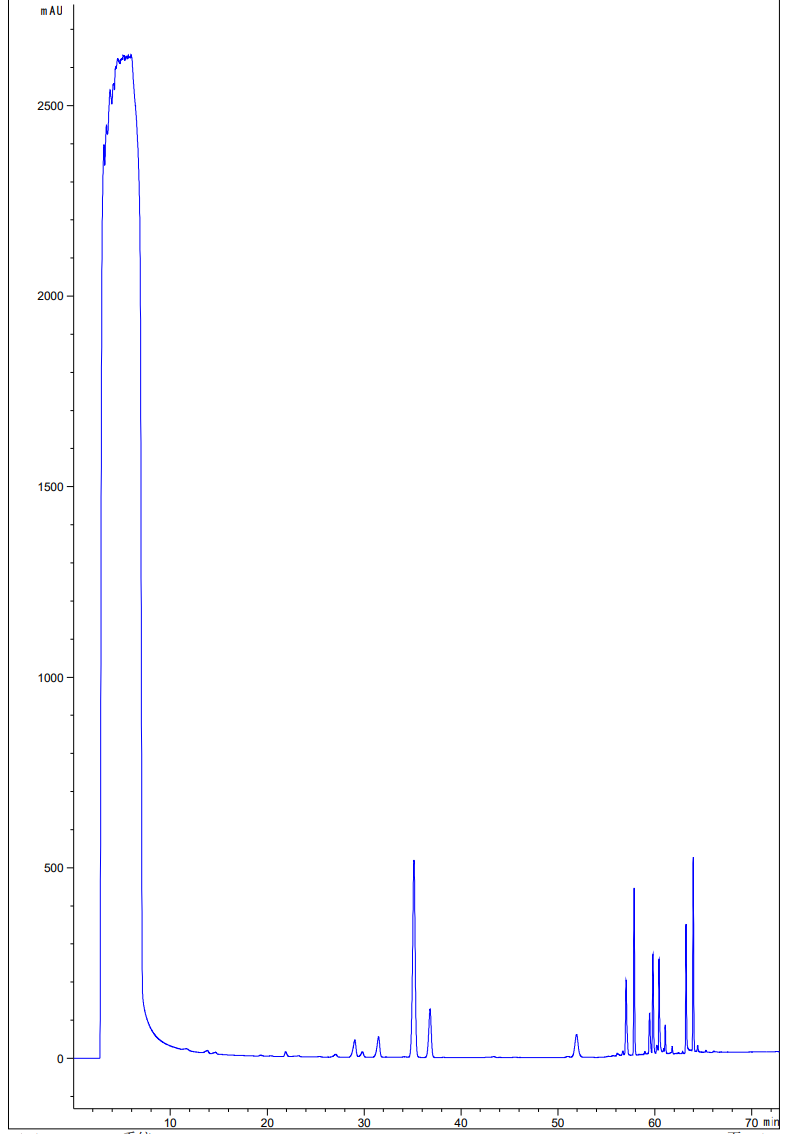


L-Araf

D-Glc

L-Araf

D-Glc

**Figure S48.** HPLC chromatogram of compound **1** after acid hydrolysis and sugar derivatization mixed with *D*-Glc standard derivative (Left: with *t*_R_; Right: without *t*_R_)


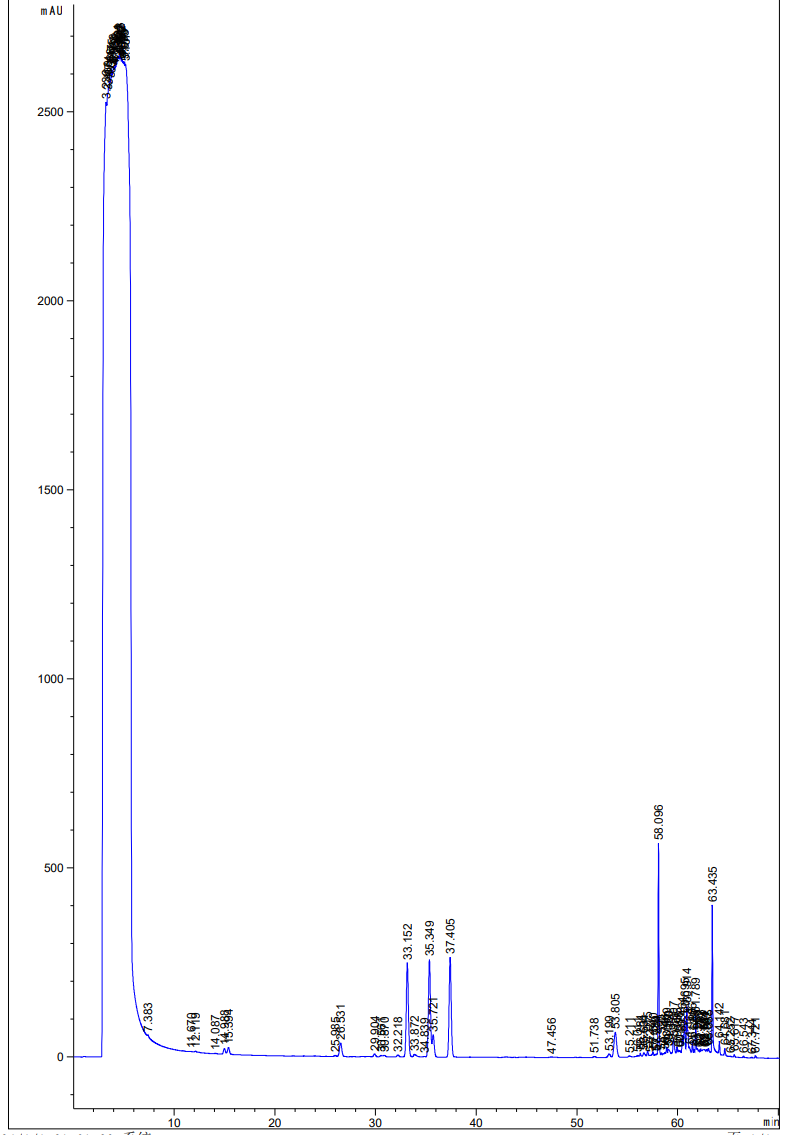

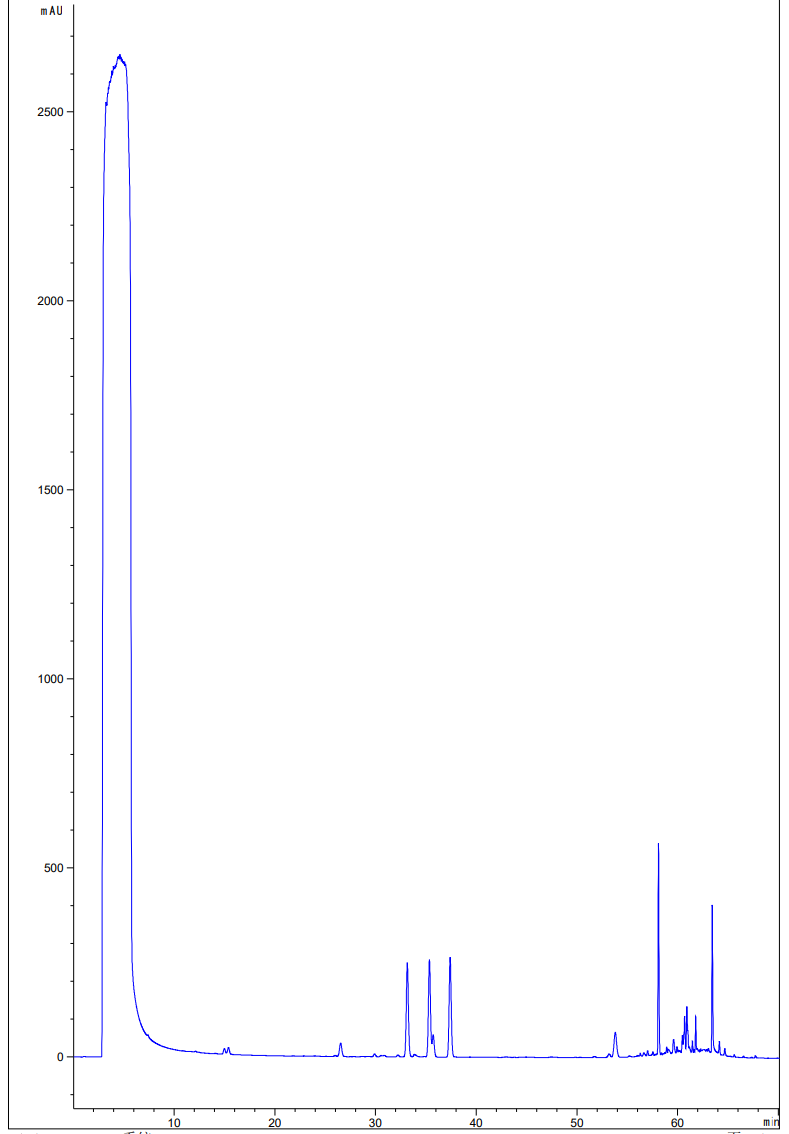


L-Araf

D-Glc

D-Glc

L-Araf

**Figure S49.** HPLC chromatogram of compound **1** after acid hydrolysis and sugar derivatization mixed with *L*-Araf standard derivative (Left: with *t*_R_; Right: without *t*_R_)

**Compound 2:**


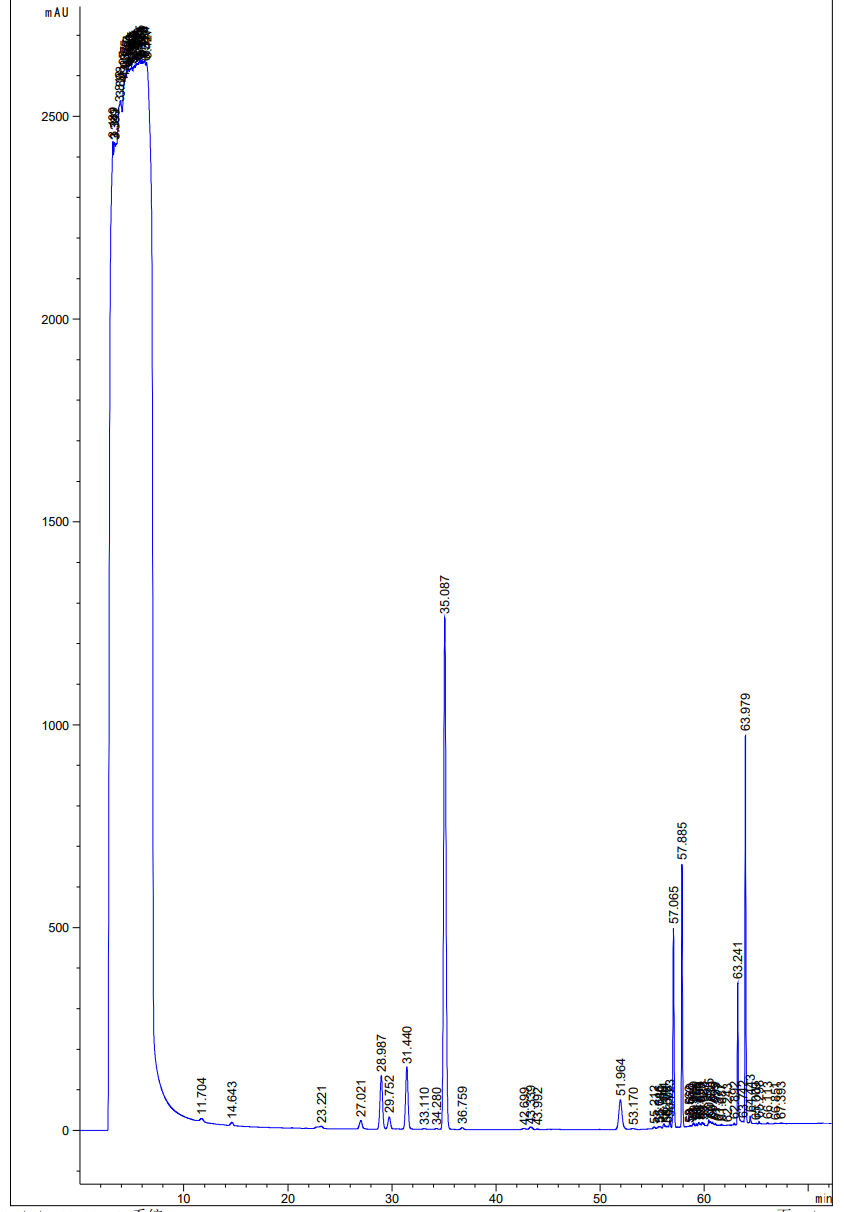

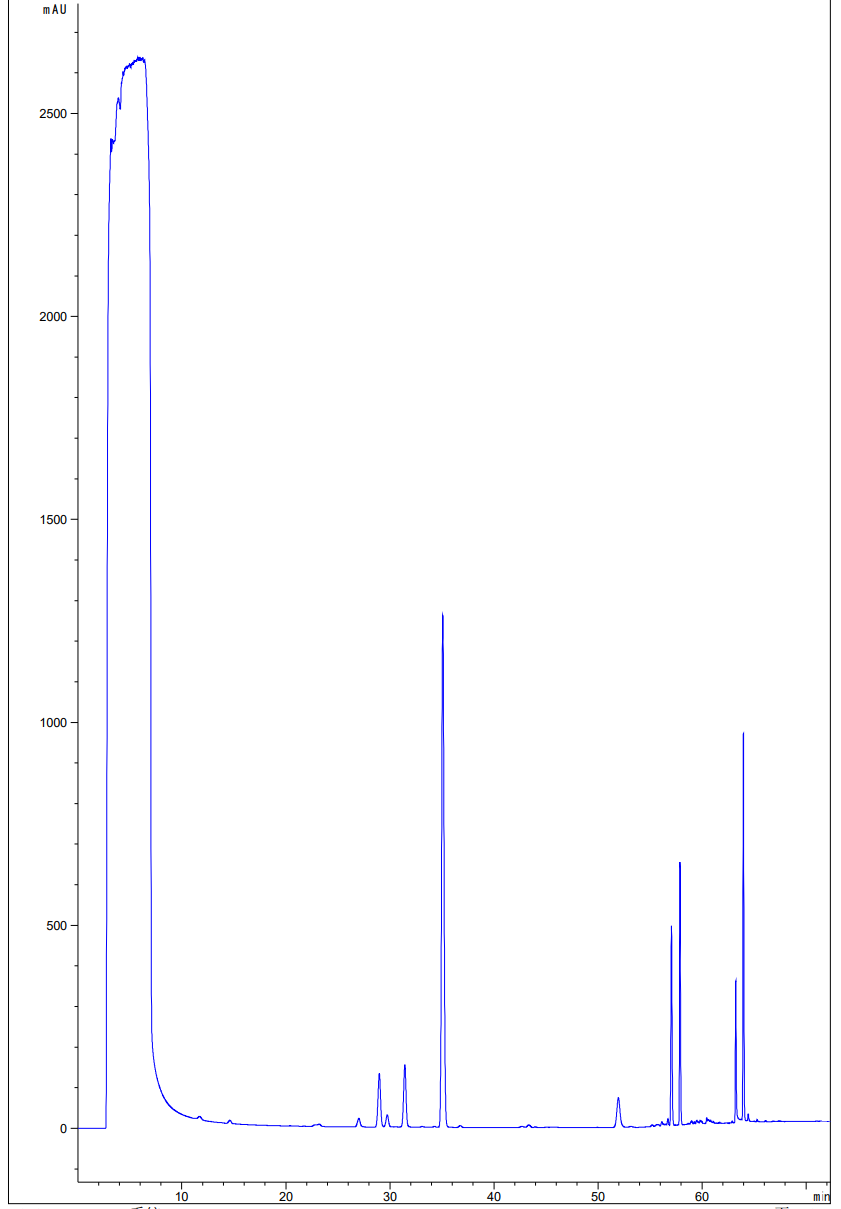


D-Glc

D-Glc

**Figure S50.** HPLC chromatogram of *D*-Glc standard derivative (Left: with *t*_R_; Right: without *t*_R_)


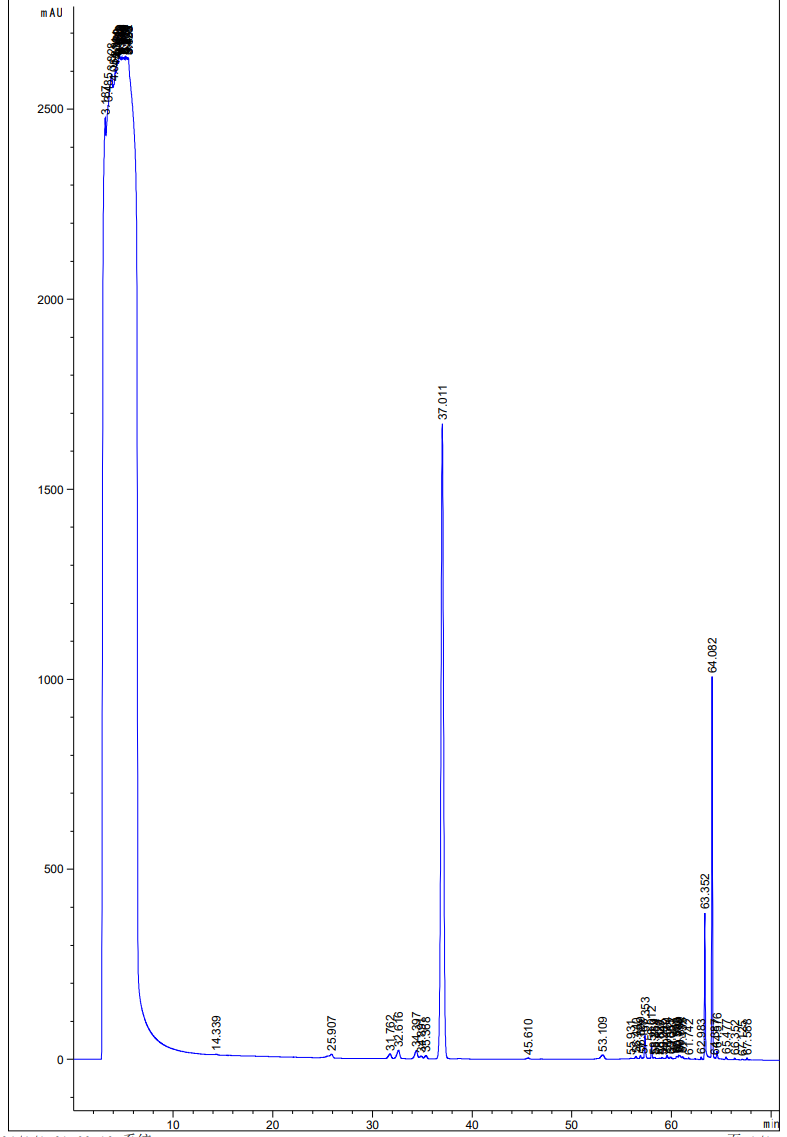

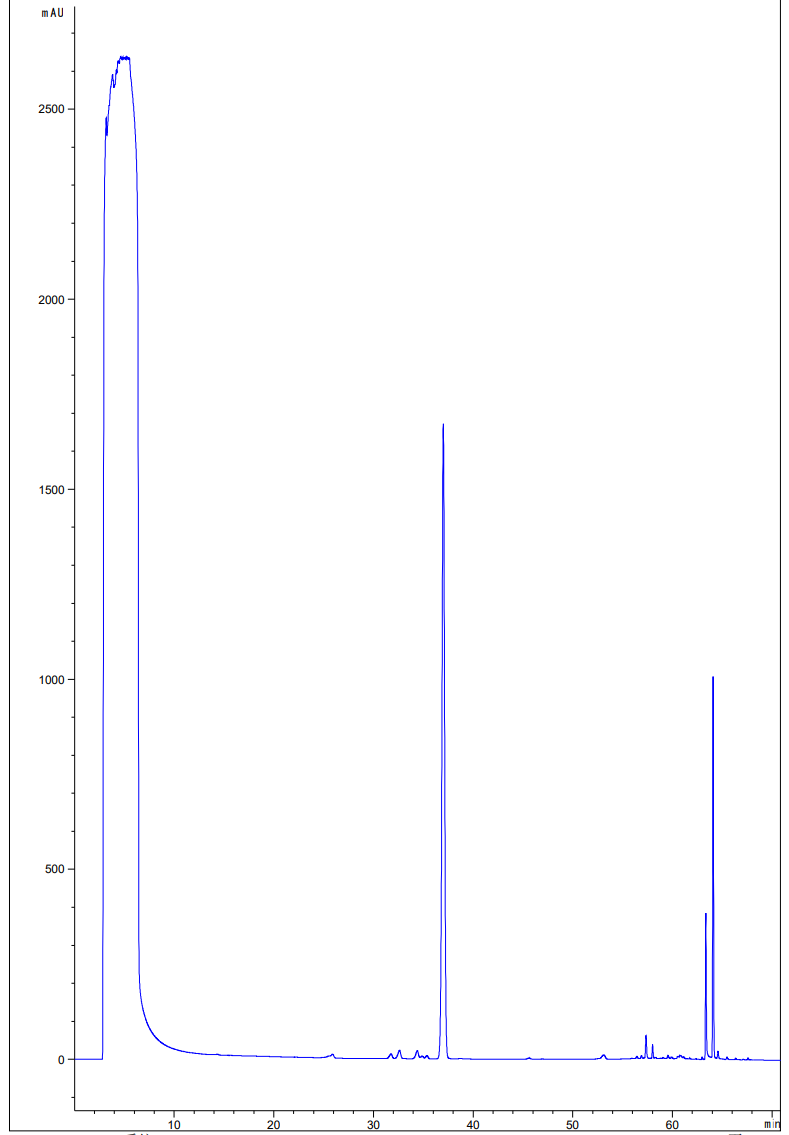


L-Arap

L-Arap

**Figure S51.** HPLC chromatogram of *L*-Arap standard derivative (Left: with *t*_R_; Right: without *t*_R_)


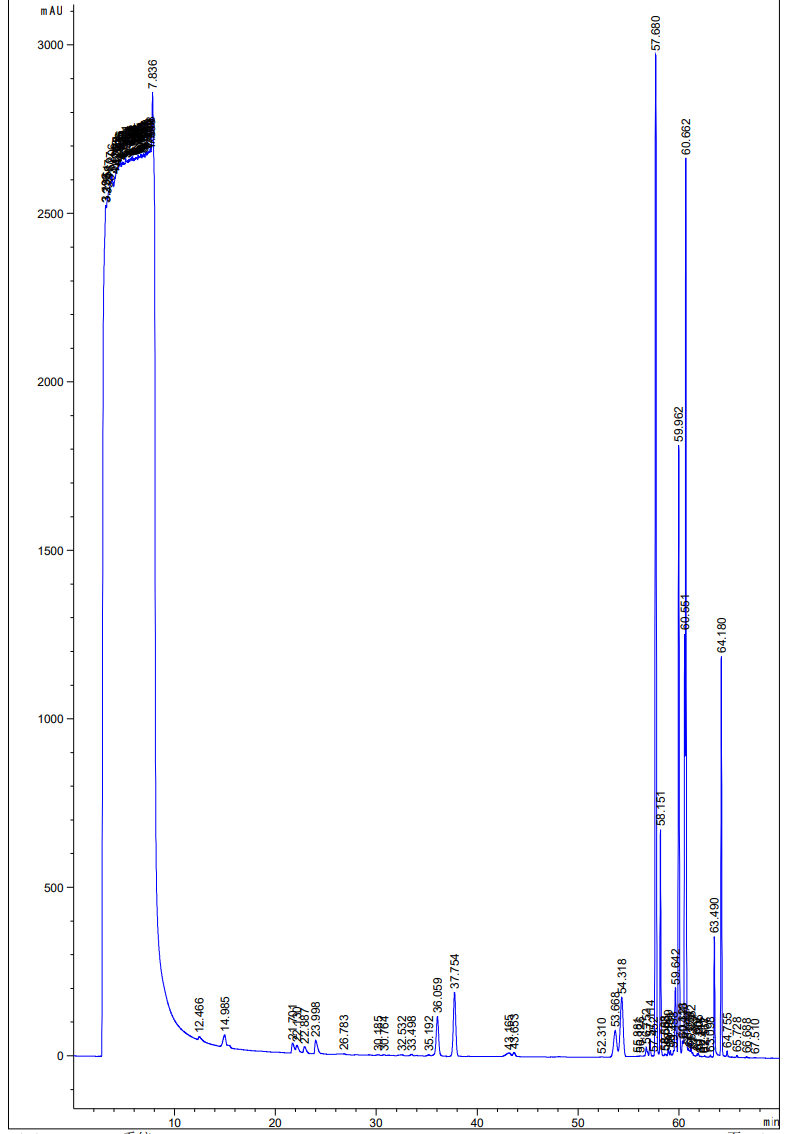

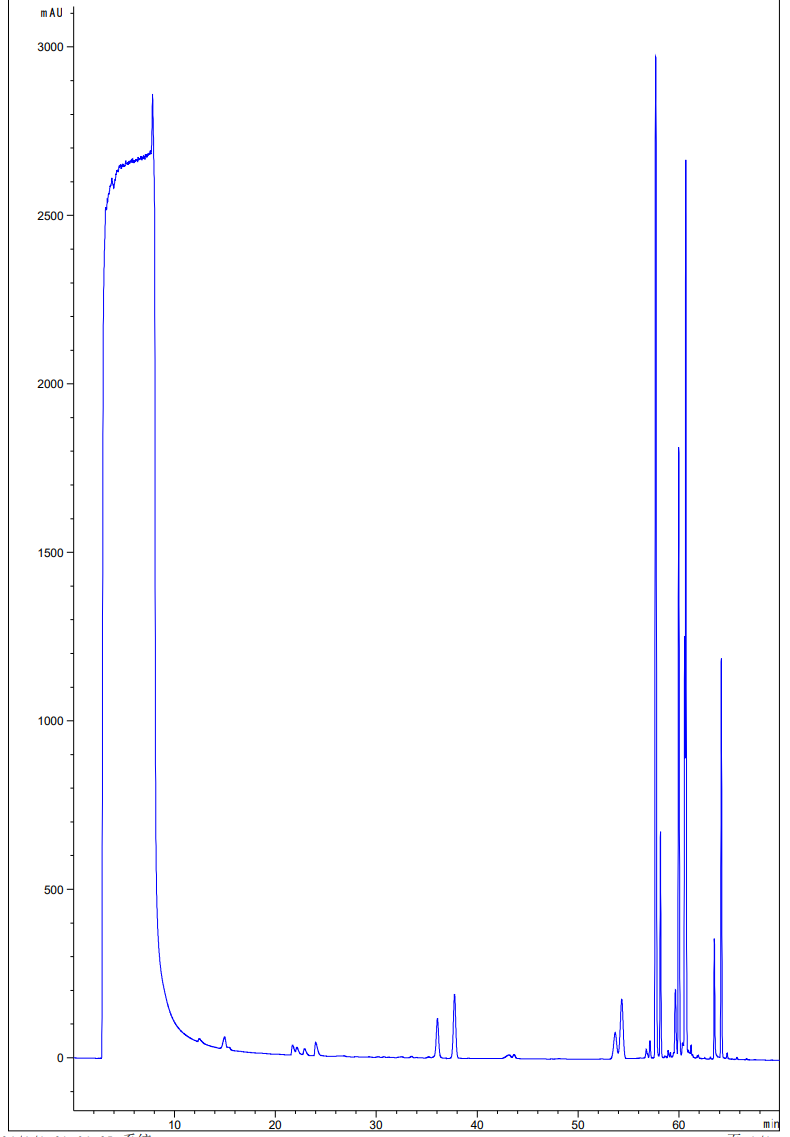


D-Glc

L-Arap

L-Arap

D-Glc

**Figure S52.** HPLC chromatogram of compound **2** after acid hydrolysis and sugar derivatization

(Left: with *t*_R_; Right: without *t*_R_)


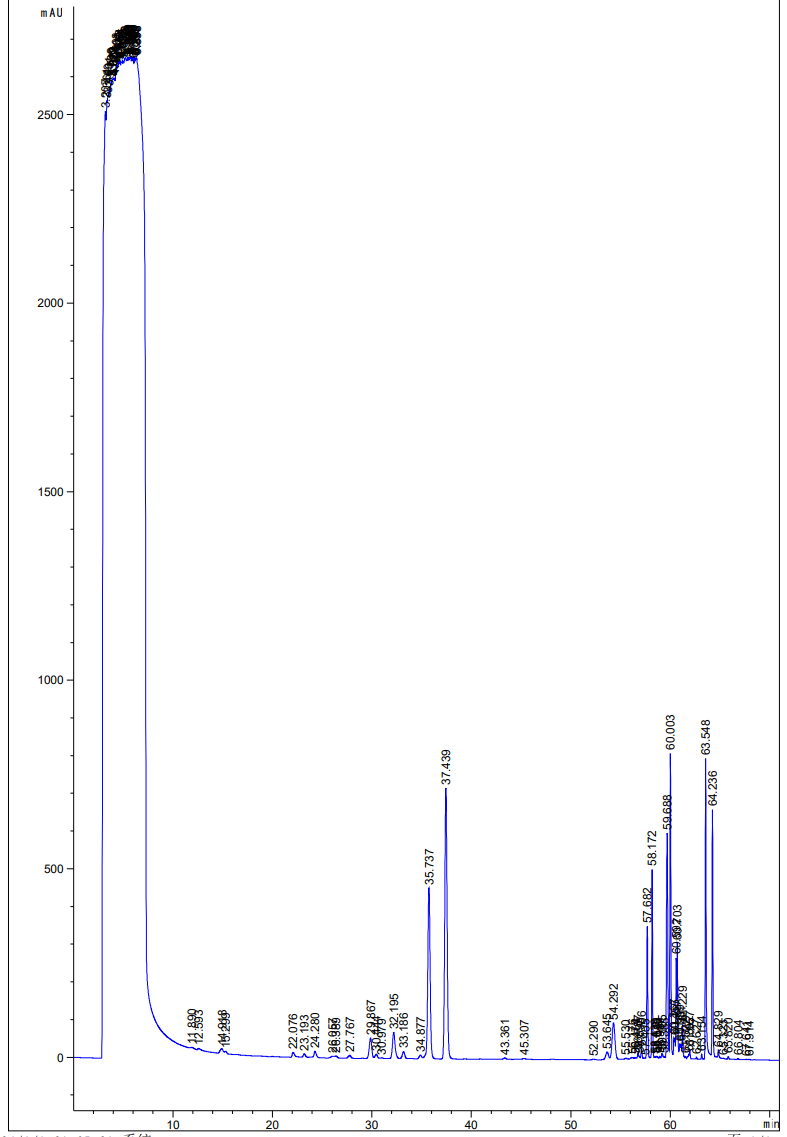

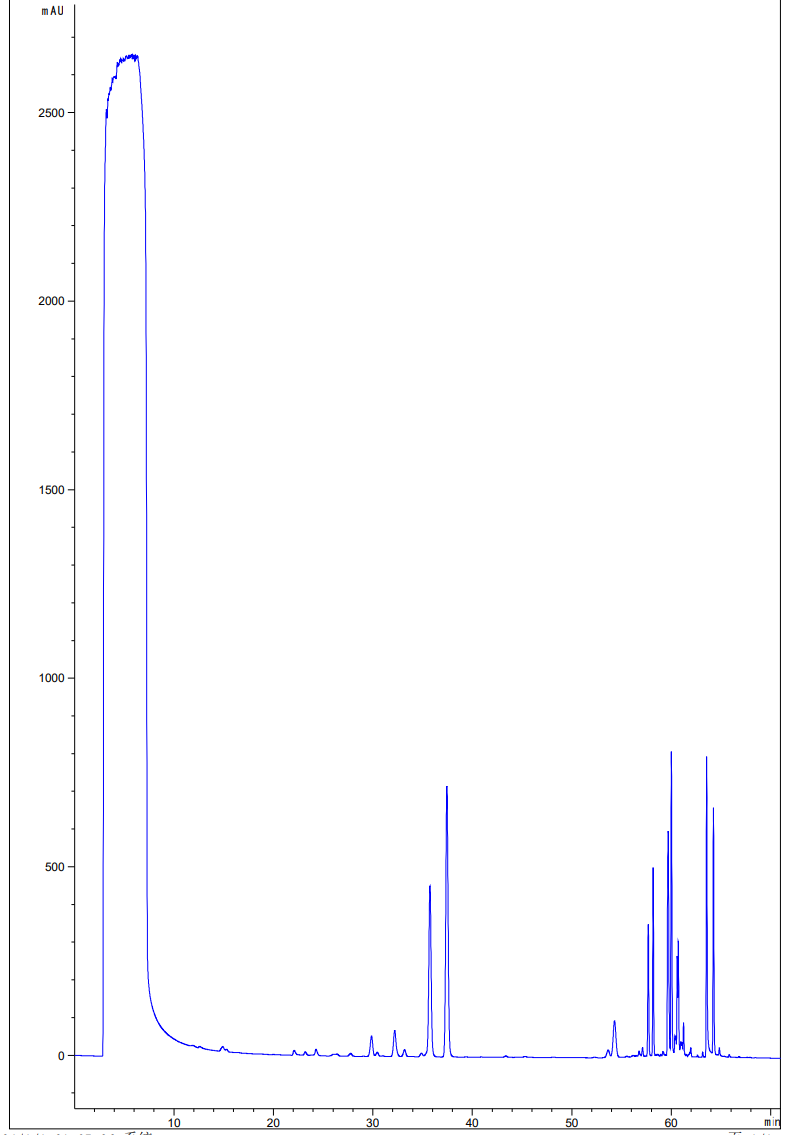


D-Glc

L-Arap

D-Glc

L-Arap

**Figure S53.** HPLC chromatogram of compound **2** after acid hydrolysis and sugar derivatization mixed with *D*-Glc and *L*-Arap standard derivatives (Left: with *t*_R_; Right: without *t*_R_)

**Compound 3:**


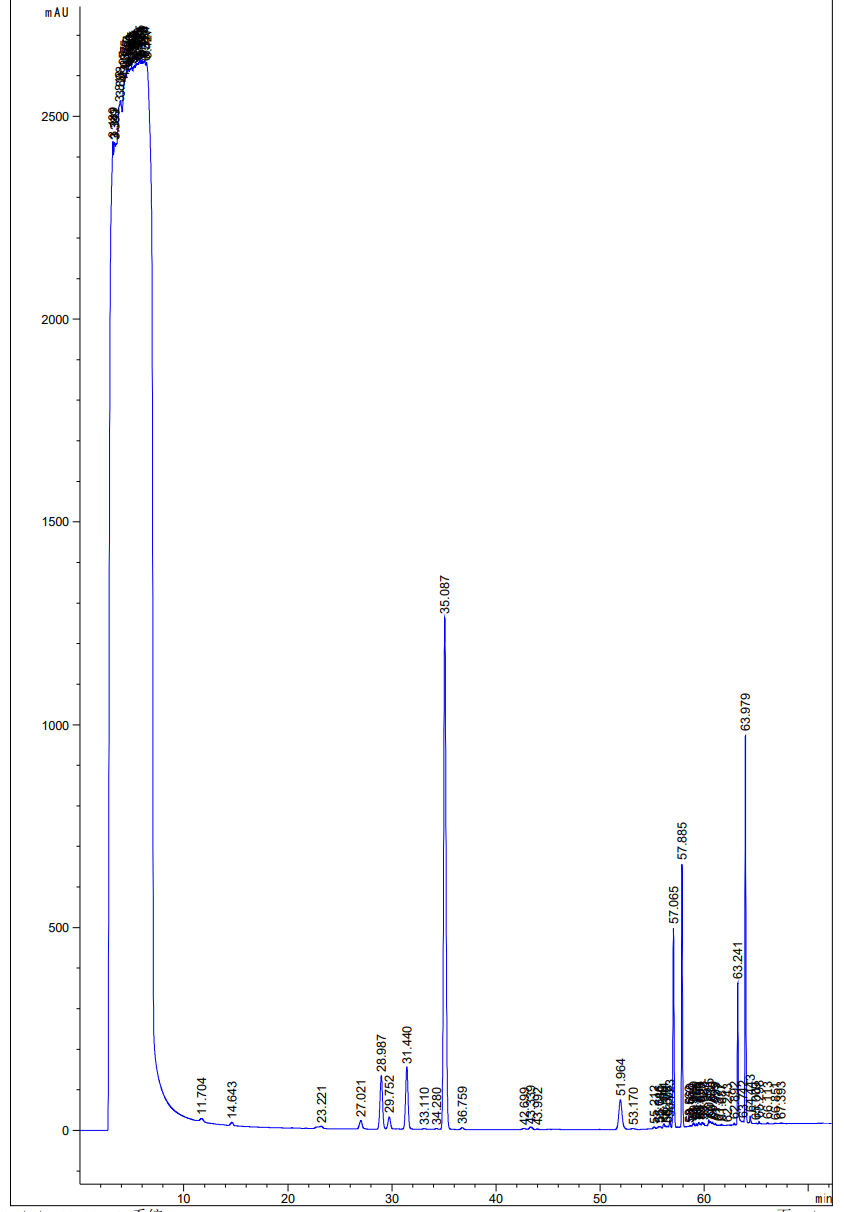

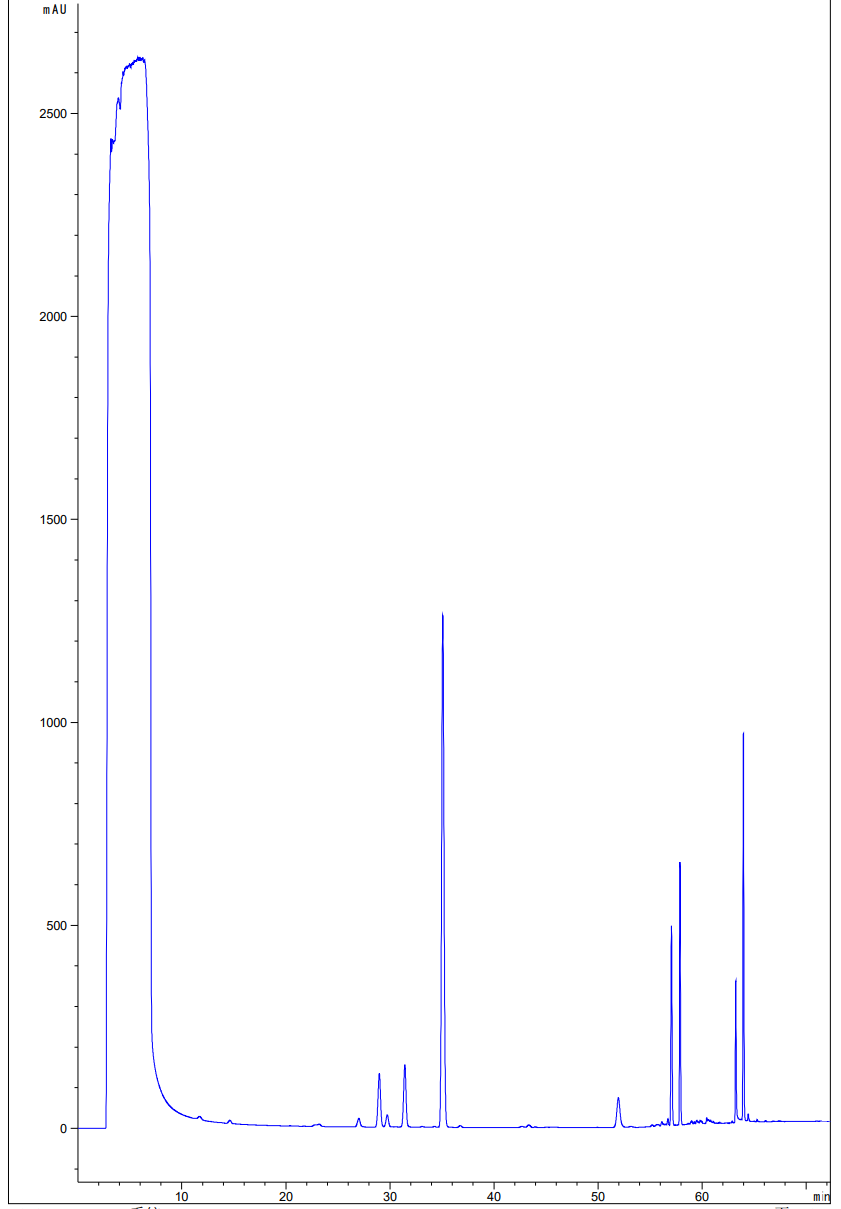


D-Glc

D-Glc

**Figure S54.** HPLC chromatogram of *D*-Glc standard derivative (Left: with *t*_R_; Right: without *t*_R_)


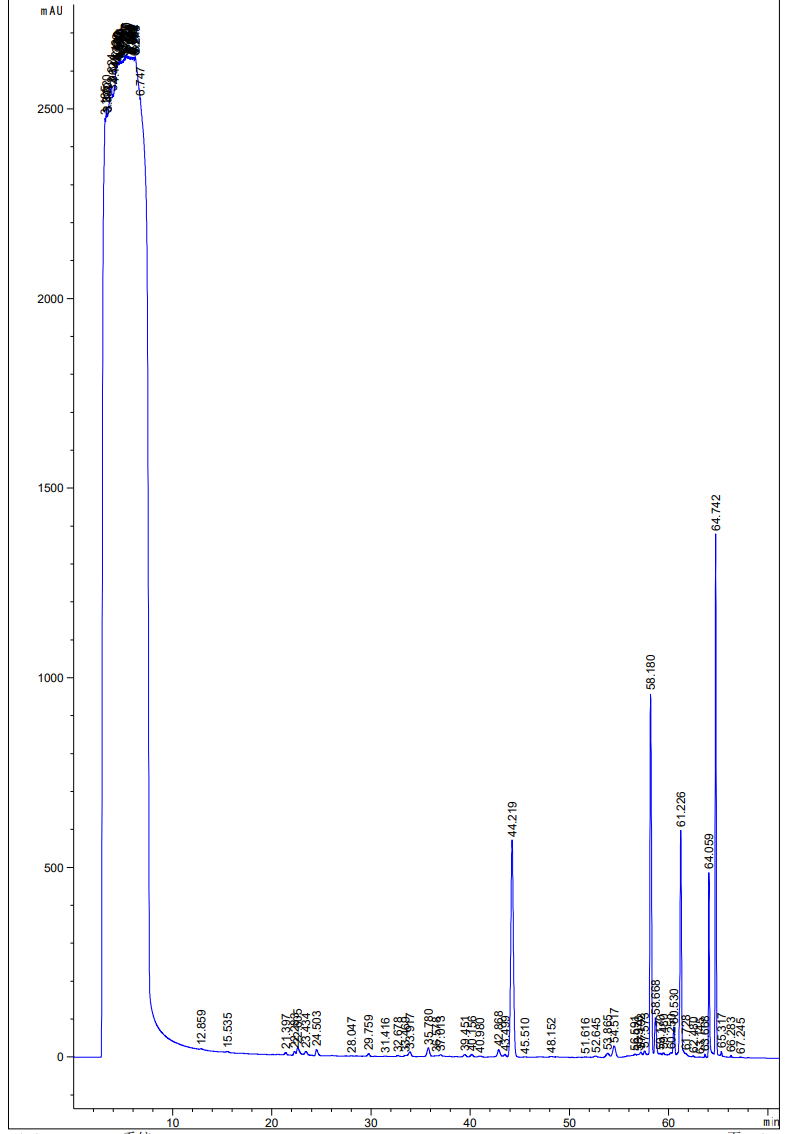

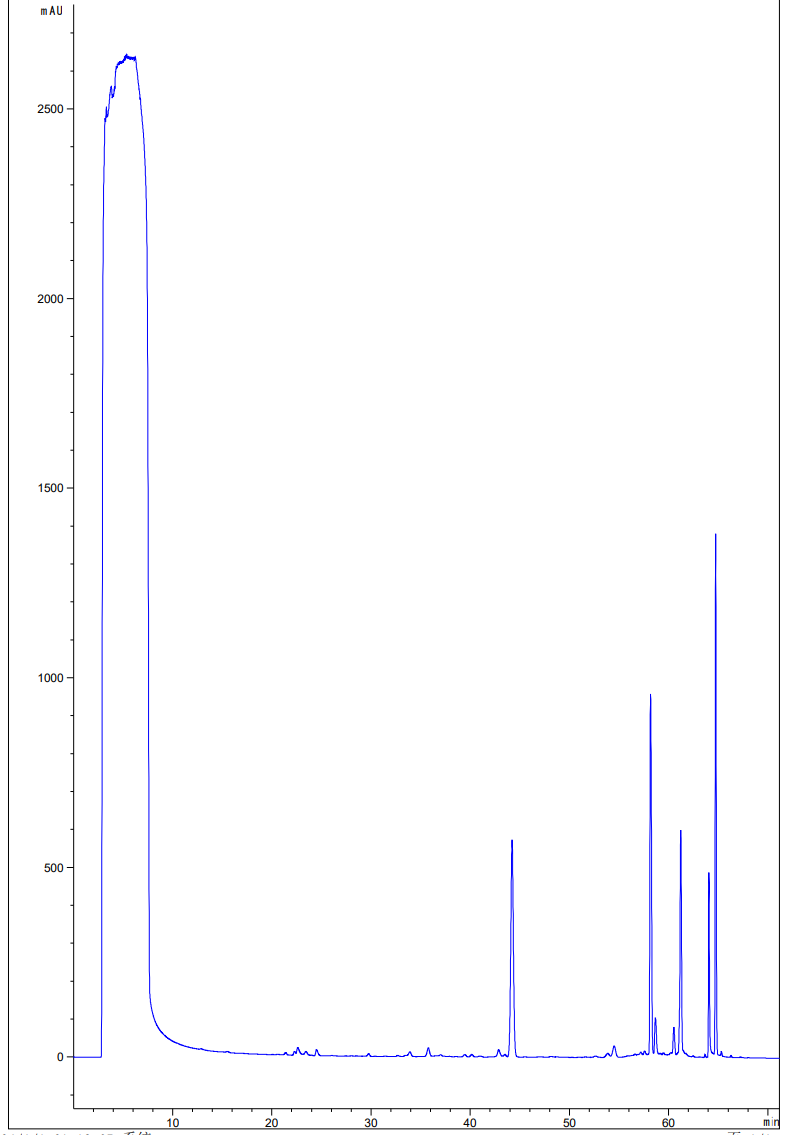


D-Api

D-Api

**Figure S55.** HPLC chromatogram of *D*-Api standard derivative (Left: with *t*_R_; Right: without *t*_R_)


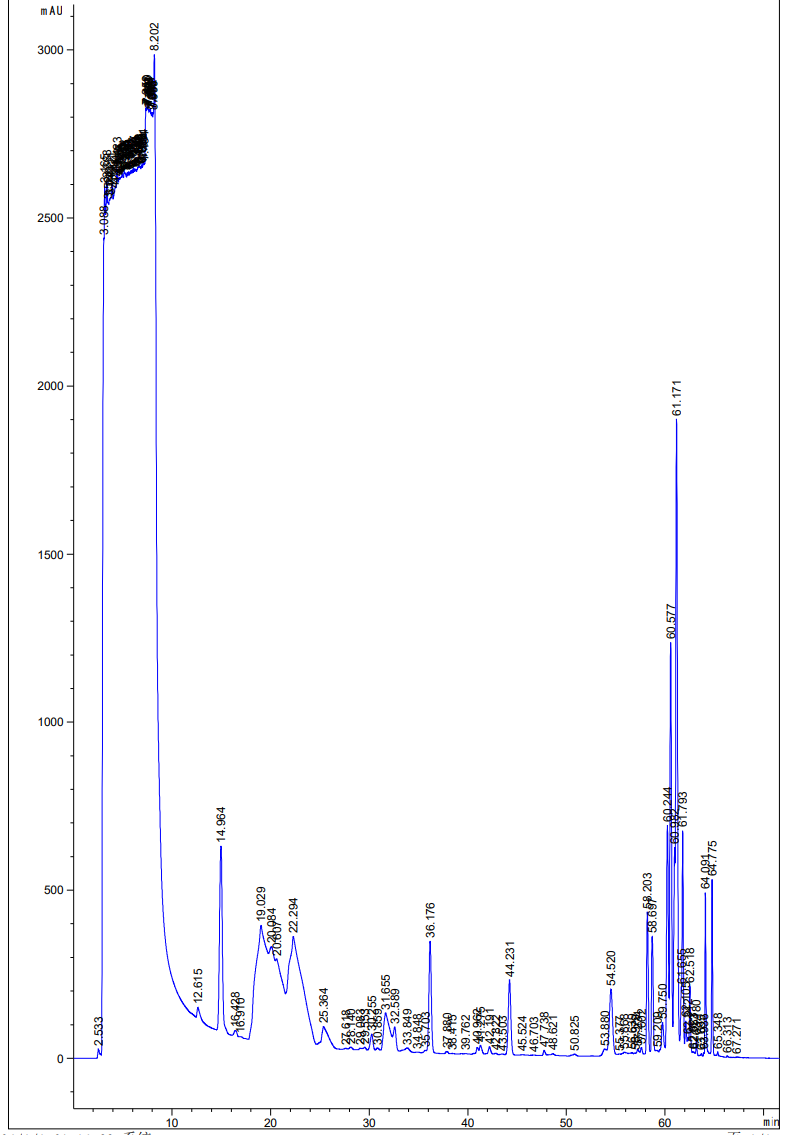

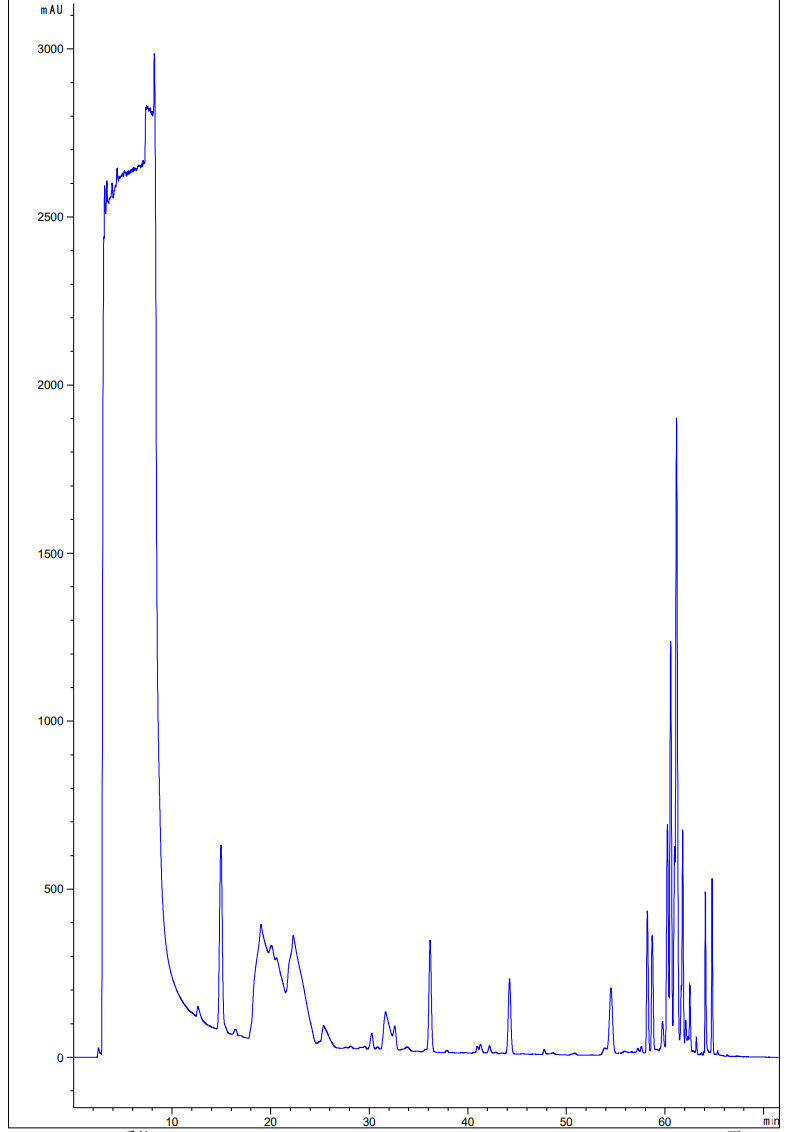


D-Api

D-Glc

D-Glc

D-Api

**Figure S56.** HPLC chromatogram of compound **3** after acid hydrolysis and sugar derivatization

(Left: with *t*_R_; Right: without *t*_R_)


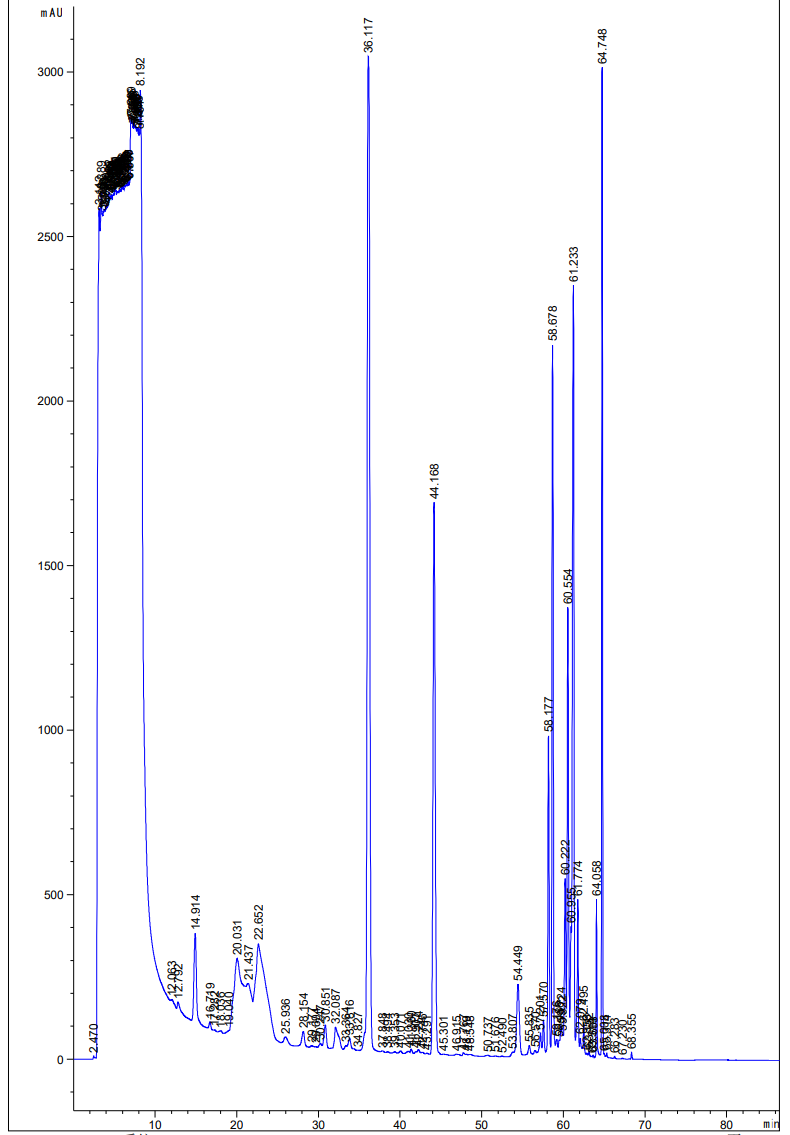

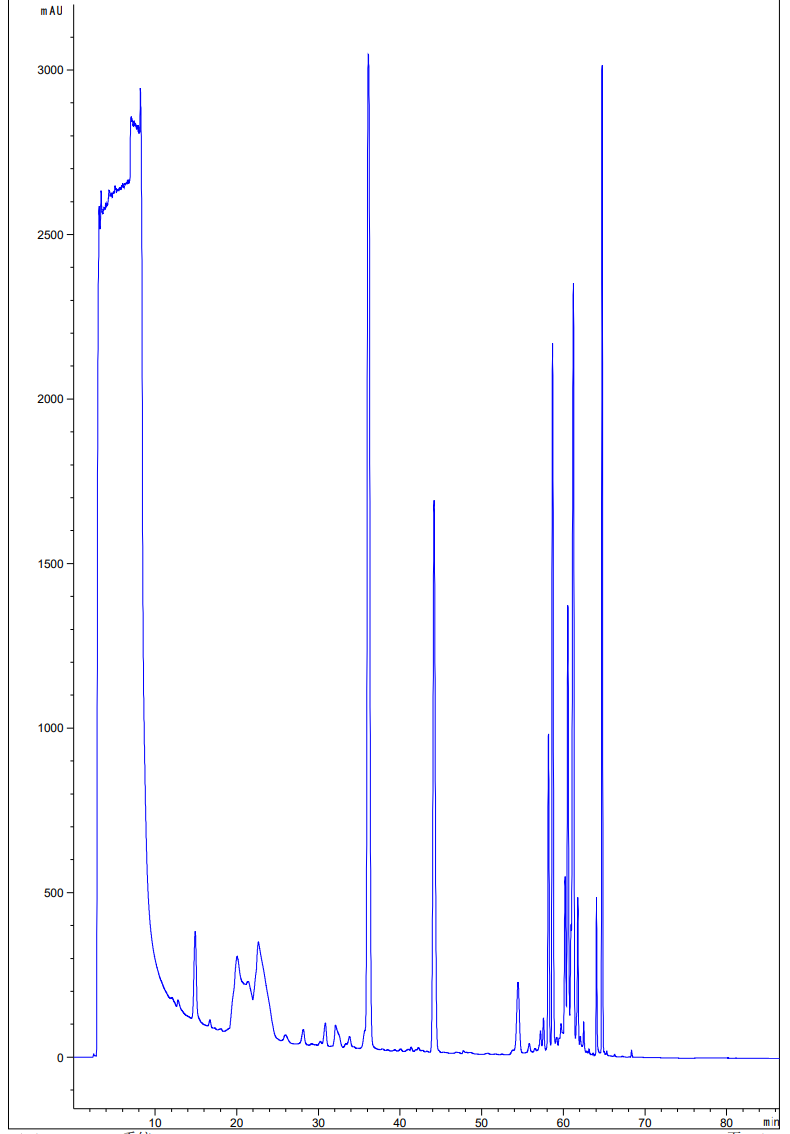


D-Api

D-Glc

D-Glc

D-Api

**Figure S57.** HPLC chromatogram of compound **3** after acid hydrolysis and sugar derivatization mixed with *D*-Glc and *D*-Api standard derivatives (Left: with *t*_R_; Right: without *t*_R_)

**3. Spectroscopic Data of Known Compounds 4-12.**

**1-(3′,5′-dimethoxy)phenyl-2-[4″-O-*β*-D-glucopyranosyl(6→1)-O-*α*-L-rhamnopyranosyl]phenylethane** **(4)** - Light yellow solid; Molecular formula: C_28_H_38_O_12_; ^1^H-NMR (CD_3_OD, 400 MHz) δ_H_: 2.80 (2H, t, *J* = 6.8 Hz, H-α), 2.81 (2H, t, *J* = 6.8 Hz, H-α′), 6.30 (2H, d, *J* = 2.3 Hz, H-2, 6), 6.27 (1H, d, *J* = 2.3 Hz, H-4), 7.10 (2H, d, *J* = 8.6 Hz, H-2′, 6′), 6.98 (2H, d, *J* = 8.6 Hz, H-3′, 5′), 3.70 (6H, s, 3, 5-OMe), 4.80 (1H, d, *J* = 7.4 Hz, Glc-H-1″), 3.45 (1H, m, Glc-H-2″), 3.43 (1H, m, Glc-H-3″), 3.37 (1H, m, Glc-H-4″), 3.63 (1H, m, Glc-H-5″), 4.02 (1H, dd, *J* = 10.8, 1.7 Hz, Glc-H-6a″), 3.84 (1H, dd, *J* = 3.5, 1.7 Hz, Glc-H-6b″), 4.71 (1H, d, *J* = 1.7 Hz, Rha-H-1′′′), 3.85 (1H, dd, *J* = 3.5, 1.7 Hz, Rha-H-2′′′), 3.66 (1H, m, Rha-H-3″′), 3.33 (1H, m, Rha-H-4″′), 3.53 (1H, m, Rha-H-5″′), 1.21 (3H, d, *J* = 6.2 Hz, Rha-H-6′′′); ^13^C-NMR (CD_3_OD, 100 MHz) δ_C_: 145.38 (C-1), 107.59 (C-2), 162.16 (C-3), 97.53 (C-4), 162.16 (C-5), 107.59 (C-6), 55.60 (C-3, 5-OMe), 39.47 (C-α), 38.00 (C-α′), 137.13 (C-1′), 130.46 (C-2′), 117.75 (C-3′), 157.34 (C-4′), 117.75 (C-5′), 130.46 (C-6′), 102.63 (Glc-C-1′′), 74.94 (Glc-C-2′′), 78.03 (Glc-C-3′′), 71.57 (Glc-C-4′′), 76.88 (Glc-C-5′′), 67.88 (Glc-C-6′′), 102.16 (Rha-C-1′′′), 72.18 (Rha-C-2′′′), 72.41 (Rha-C-3′′′), 74.03 (Rha-C-4′′′), 69.84 (Rha-C-5′′′), 17.95 (Rha-C-6′′′).

**Sasastilboside A (5)** - White powder; Molecular formula: C_21_H_26_O_8_; ^1^H-NMR (CD_3_OD, 400 MHz) δ_H_: 6.20 (2H, m, H-2, 6), 6.16 (1H, t, *J* = 2.2 Hz, H-4), 3.69 (3H, s, 3- OCH_3_), 7.08 (2H, d, *J* = 8.0 Hz, H-2′, 6′), 6.98 (2H, d, *J* = 8.0 Hz, H-3′, 5′), 2.78 (4H, m, H-α*,* H-α′) , 4.86 (1H, d, *J* = 6.4 Hz, Glc-H-1′′) , 3.39-3.45 (4H, m, Glc-H-2′′, 3′′, 4′′, 5′′), 3.88 (1H, dd, *J* = 11.9, 1.8, Glc-H-6′′a), 3.68 (1H, m, Glc-H-6′′b); ^13^C-NMR (CD_3_OD, 100 MHz) δ_C_: 145.33 (C-1), 109.07 (C-2), 162.20 (C-3), 99.87 (C-4), 159.38 (C-5), 106.57 (C-6), 39.43 (C-α), 38.00 (C-α′), 55.50 (C-3-OCH_3_), 137.06 (C-1′), 130.41 (C-2′), 117.60 (C-3′), 157.39 (C-4′), 117.60 (C-5′), 130.41 (C-6′), 102.50 (Glc-C-1′′), 74.93 (Glc-C-2′′), 77.98 (Glc-C-3′′), 71.37 (Glc-C-4′′), 78.09 (Glc-C-5′′), 62.50 (Glc-C-6′′).

**3,5-dihydroxydihydrostilbene 4′-O-*β*-D-glucopyranoside (6)** - White powder; Molecular formula: C_20_H_24_O_8_; ^1^H-NMR (CD_3_OD, 400 MHz) δ_H_ : 2.71 (2H, t, *J* = 6.3 Hz, H-α), 2.82 (2H, t, *J* = 6.3 Hz, H-α′), 6.38 (2H, brs, H-2, 6), 6.30 (1H, brs, H-4), 7.07 (2H, d, *J* = 8.6 Hz, H-2′, 6′), 6.98 (2H, d, *J* = 8.6 Hz, H-3′, 5′), 4.85 (1H, d, *J* = 7.1 Hz, Glc-H-1″), 3.45 (1H, m, Glc-H-2″), 3.40 (1H, m, Glc-H-3″), 3.39 (1H, m, Glc-H-4″), 3.70 (1H, m, Glc-H-5″), 3.90 (1H, m, Glc-H-6a″), 3.73 (1H, m, Glc-H-6b″); ^13^C-NMR (CD_3_OD, 100 MHz) δ_C_: 145.34 (C-1), 108.01 (C-2), 159.32 (C-3), 101.13 (C-4), 159.32 (C-5), 108.01 (C-6), 39.37 (C-α), 38.01 (C-α′), 137.12 (C-1′), 130.36 (C-2′), 117.57 (C-3′), 157.37 (C-4′), 117.57 (C-5′), 130.36 (C-6′), 102.48 (Glc-C-1′′), 74.93 (Glc-C-2′′), 77.97 (Glc-C-3′′), 71.37 (Glc-C-4′′), 78.08 (Glc-C-5′′), 62.49 (Glc-C-6′′).

**5,4′-dihydroxydihydrostilbene 3-O-*β*-D-glucopyranoside (7) -** White amorphous powder; Molecular formula: C_20_H_24_O_8_; ^1^H-NMR (CD_3_OD, 400 MHz) δ_H_: 6.67 (2H, d, *J* = 8.5 Hz, H-2′, 6′), 6.40 (2H, d, *J* = 8.5 Hz, H-3′, 5′), 6.37 (2H, m, H-2, 6), 6.28 (1H, m, H-4), 4.79 (1H, d, *J* = 7.2 Hz, Glc-H-1″), 3.87 (1H, d, *J* = 11.8 Hz, Glc-H-6a″), 3.69 (1H, m, Glc-H-6b″), 3.46 (4H, m, Glc-H-2′′, 3′′, 4′′, 5″), 2.77 (2H, brs, H-α), 2.72 (2H, brs, H-α′); ^13^C-NMR (CD_3_OD, 100 MHz) δ_C_: 145.61 (C-1), 109.23 (C-2), 160.08 (C-3), 102.61 (C-4), 159.21 (C-5), 110.71 (C-6), 133.94 (C-1′), 130.45 (C-2′, 6′), 116.01 (C-3′, 5′), 156.41 (C-4′), 39.55 (C-α), 37.97 (C-α′), 102.24 (Glc-C-1′′), 74.89 (Glc-C-2′′), 77.99 (Glc-C-3′′), 71.35 (Glc-C-4′′), 78.05 (Glc-C-5′′), 62.47 (Glc-C-6′′).

**3,5-dihydroxyldihydrostilbene 4′-O-[6″-O-(4″′-methoxylgalloyl)]-*β*-D- glucopyranoside** **(8)** - White amorphous powder; Molecular formula: C_28_H_30_O_12_; ^1^H-NMR (CD_3_OD, 400 MHz) δ_H_: 6.10 (2H, d, *J* = 2.2 Hz, H-2, H-6), 6.06 (1H, t, *J* = 2.2 Hz, H-4), 2.67 (2H, m, H-α), 2.76 (2H, m, H-α′), 6.98 (d, *J* = 8.7 Hz, H-2′, H-6′), 6.95 (d, *J* = 8.7 Hz, H-3′, H-5′), 4.83 (1H, d, *J* = 7.4 Hz, Glc-H-1′′), 3.49 (1H, m, Glc-H-2′′), 3.52 (1H, m, Glc-H-3′′), 3.45 (1H, m, Glc-H-4′′), 3.77 (1H, m, Glc-H-5′′), 4.43 (1H, dd, *J* = 7.7, 11.9 Hz, Glc-H-6′′a), 4.62 (1H, dd, *J* = 2.2, 11.8 Hz, Glc-H-6′′b), 7.10 (2H, s, H-2′′′, H-6′′′), 3.87 (3H, s, 4′′′-OMe); ^13^C-NMR (CD_3_OD, 100 MHz) δ_C_: 145.5 (C-1), 108.0 (C-2, 6), 159.3 (C-3, 5), 101.1 (C-4), 39.3 (C-α), 38.1 (C-α′), 137.3 (C-1′), 130.3 (C-2′, 6′), 117.6 (C-3′, 5′), 157.1 (C-4′), 102.5 (Glc-C-1′′), 74.9 (Glc-C-2′′), 78.0 (Glc-C-3′′), 72.0 (Glc-C-4′′), 75.5 (Glc-C-5′′), 65.2 (Glc-C-6′′), 126.5 (C-1′′′), 110.4 (C-2′′′, 6′′′), 145.5 (C-3′′′, 5′′′), 141.3 (C-4′′′), 60.86 (4′′′-OMe), 167.7 (C=O, C-7′′′).

**Quercitrin (9) -** Yellow powder; Molecular formula: C_21_H_20_O_11_; ^1^H-NMR (CD_3_OD, 400 MHz) δ_H_: 7.33 (1H, s, H-2′), 7.29 (1H, d, *J* = 8.2 Hz, H-6′), 6.90 (1H, d, *J* = 8.2 Hz, H-5′), 6.36 (1H, s, H-8), 6.19 (1H, s, H-6), 5.34 (1H, s, Rha-H-1′′), 3.39-3.73 (4H, m, Rha-H-2''~H-5''), 0.93 (3H, d, *J* = 6.0 Hz, Rha-H-6′′); ^13^C-NMR (CD_3_OD, 100 MHz) δ_C_: 158.53 (C-2), 136.23 (C-3), 179.65 (C-4, C=O), 159.32 (C-5), 99.81 (C-6), 165.87 (C-7), 94.70 (C-8), 163.22 (C-9), 105.90 (C-10), 122.97 (C-1′), 116.37 (C-2′), 146.42 (C-3′), 149.80 (C-4′), 116.93 (C-5′), 122.86 (C-6′), 103.54 (Rha-C-1′′), 72.03 (Rha-C-2′′), 72.11 (Rha-C-3′′), 73.25 (Rha-C-4′′), 71.90 (Rha-C-5′′), 17.65 (Rha-C-6′′).

**Phlorizin (10) -** Light yellow powder; Molecular formula: C_21_H_24_O_10_; ^1^H-NMR (CD_3_OD, 400 MHz) δ_H_: 7.06 (2H, d, *J* = 8.4 Hz, H-2, 6), 6.68 (2H, d, *J* = 8.4 Hz, H-3, 5), 5.95 (1H, d, *J* = 2.2 Hz, H-3'), 6.17 (1H, d, *J* = 2.2 Hz, H-5'), 3.45 (2H, t, *J* = 7.6 Hz, H-α), 2.87 (2H, t, *J* = 7.6 Hz, H-β), 5.03 (1H, d, *J* = 7.2 Hz, Glc-H-1''), 3.48-3.58 (4H, m, Glc-H-2''~H-5''), 3.89 (1H, dd, *J* = 12.1, 2.1 Hz, Glc-H-6''a), 3.72 (1H, m, Glc-H-6''b); ^13^C-NMR (CD_3_OD, 100 MHz) δ_C_: 133.9 (C-1), 130.4 (C-2, 6), 116.1 (C-3, 5), 156.4 (C-4), 57.0 (C-α), 30.9 (C-β), 206.6 (C=O, C-γ), 106.8 (C-1′), 162.3 (C-2′), 98.3 (C-3′), 167.6 (C-4′), 95.4 (C-5′), 165.9 (C-6′), 102.1 (Glc-C-1''), 74.7 (Glc-C-2''), 78.4 (Glc-C-3''), 71.1 (Glc-C-4''), 78.5 (Glc-C-5''), 62.4 (Glc-C-6'').

**Quercetin-3-O-*β*-D-galactopyranoside (11) -** Yellow powder; Molecular formula: C_21_H_20_O_12_; ^1^H-NMR (CD_3_OD, 400 MHz) δ_H_: 6.20 (1H, d, *J* = 2.0 Hz, H-6), 6.39 (1H, d, *J* = 2.0 Hz, H-8), 7.84 (1H, d, *J* = 2.1 Hz, H-2′), 6.86 (1H, d, *J* = 8.5 Hz, H-5′), 7.58 (1H, dd, *J* = 2.1, 8.5 Hz, H-6′), 5.16 (1H, d, *J* = 7.8 Hz, Gal-H-1''), 3.46-3.84 (6H, m, Gal-H-2''~H-6''); ^13^C-NMR (CD_3_OD, 100 MHz) δ_C_: 158.46 (C-2), 135.74 (C-3), 179.53 (C-4), 163.03 (C-5), 99.92 (C-6), 166.20 (C-7), 94.72 (C-8), 158.75 (C-9), 105.58 (C-10), 122.85 (C-1′), 116.07 (C-2′), 145.82 (C-3′), 149.96 (C-4′), 117.75 (C-5′), 122.90 (C-6′), 105.35 (Gal-C-1′′), 73.16 (Gal-C-2′′), 75.08 (Gal-C-3′′), 70.01 (Gal-C-4′′), 77.18 (Gal-C-5′′), 61.91 (Gal-C-6′′).

**Quercetin-3-O-*β*-D-glucopyranoside (12) -** Yellow powder; Molecular formula: C_21_H_20_O_12_; ^1^H-NMR (CD_3_OD, 400 MHz) δ_H_: 6.19 (1H, d, *J* = 1.7 Hz, H-6), 6.39 (1H, s, H-8), 7.70 (1H, d, *J* = 2.0 Hz, H-2′), 6.86 (1H, d, *J* = 8.5 Hz, H-5′), 7.58 (1H, dd, *J* = 2.0, 8.5 Hz, H-6′), 5.25 (1H, d, *J* = 7.4 Hz, Glc-H-1''), 3.20-3.84 (6H, m, Glc-H-2''~H-6''); ^13^C-NMR (CD_3_OD, 100 MHz) δ_C_: 158.48 (C-2), 135.59 (C-3), 179.48 (C-4), 163.07 (C-5), 99.90 (C-6), 166.12 (C-7), 94.70 (C-8), 158.98 (C-9), 105.66 (C-10), 123.06 (C-1′), 115.99 (C-2′), 145.92 (C-3′), 149.86 (C-4′), 117.52 (C-5′), 123.18 (C-6′), 104.23 (Glc-C-1′′), 75.71 (Glc-C-2′′), 78.10 (Glc-C-3′′), 71.19 (Glc-C-4′′), 78.40 (Glc-C-5′′), 62.52 (Glc-C-6′′).

**Figure S58.** Chemical structures of the known compounds **4-12**.

**4.Bioassay**

*4.1. MTT assay for cell viability*

RAW264.7 macrophages were maintained at 5 × 10^5^ cells/mL in DMEM medium supplemented with 10% heat-inactivated FBS, penicillin G (100 U/mL), streptomycin (100 mg/L), and L-glutamine (2 mM) and incubated at 37 °C in a humidified atmosphere containing 5% CO_2_. Cell viability was determined by adding 100 mg/mL of MTT to 1 mL of a cell suspension (1 × 10^5^ cells/mL in 96-well plates) and incubated for 30 min. The formazan formed was dissolved in acidic 2-propanol, and the optical density was measured at 540 nm.

*4.2. Nitrite assay*

The concentration of nitric oxide (NO) in the conditioned media was determined by a method based on the Griess reaction (Titheradge. 1998). An aliquot of each supernatant (100 mL) was mixed with the same volume of Griess reagent (0.1% (w/v) N-(1-naphathyl)-ethylenediamine and 1% (w/v) sulfanilamide in 5% (v/v) phosphoric acid) for 10 min at room temperature. The absorbance of the final product was measured spectrophotometrically at 540 nm using an ELISA plate reader. The nitrite concentration in the samples was determined from a standard curve of sodium nitrite prepared in phenol red-free DMEM.

*4.3. Statistical analysis*

The results are presented as the mean ± standard deviation (S.D.). Statistical analysis of normally distributed data was conducted using one-way ANOVA (Newman Keuls t-test) to evaluate differences between mean values. Statistical significance was considered at *p* < 0.05.

**Reference**

1. M.A. Titheradge, The enzymatic measurement of nitrate and nitrite, Methods Mol. Biol. 100 (1998) 83-91.
